# Supplementary figures and images for: Quinine Esters with 1,2-Azole, Pyridine and Adamantane Fragments
Source: Molecules. 2022 May 27;27(11):3476. doi: 10.3390/molecules27113476 (PMC9182173; doi:10.3390/molecules27113476)

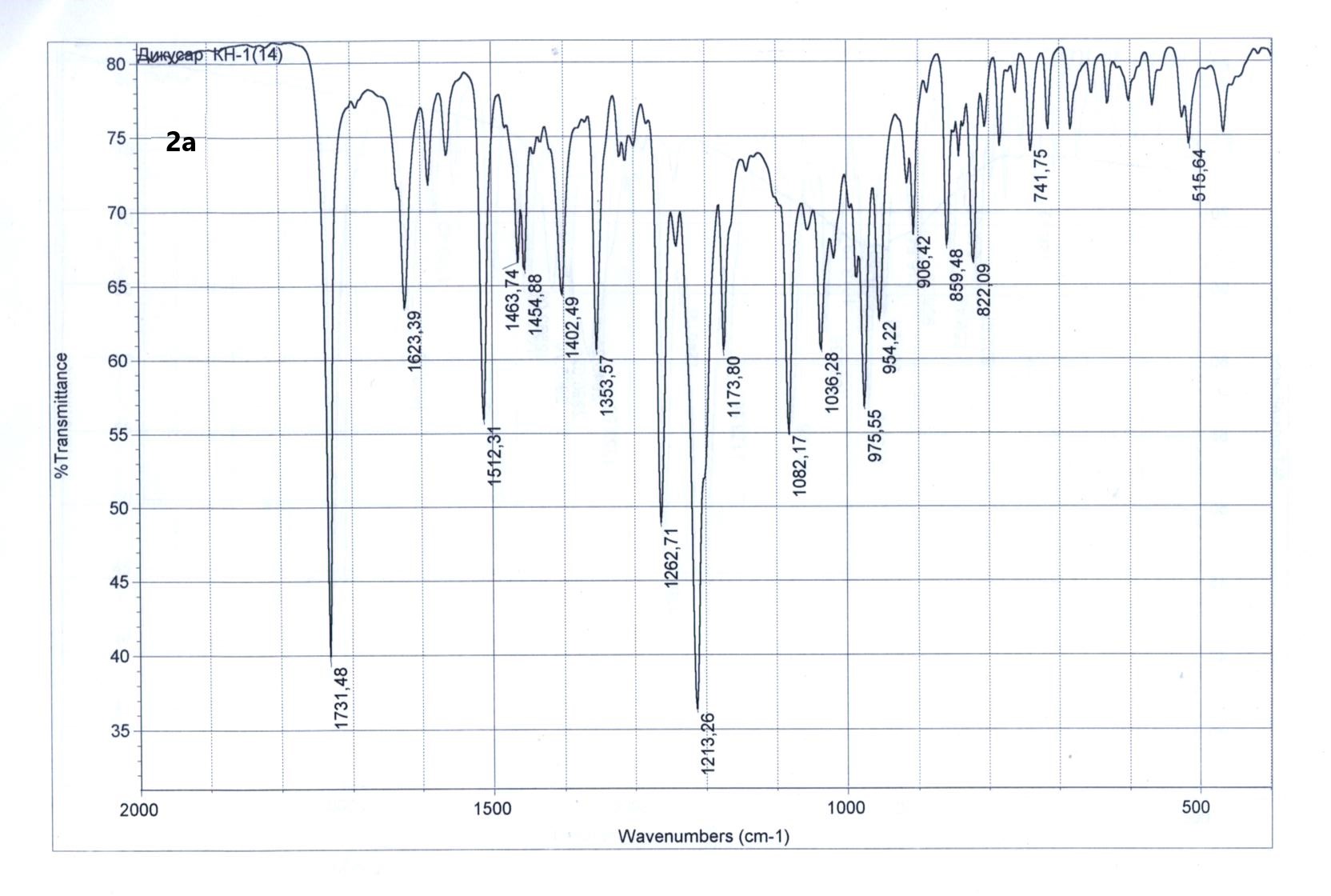

Supplement: Supplementary file 1 [file molecules-27-03476-s001.zip › IR/2a (IR1).jpg]

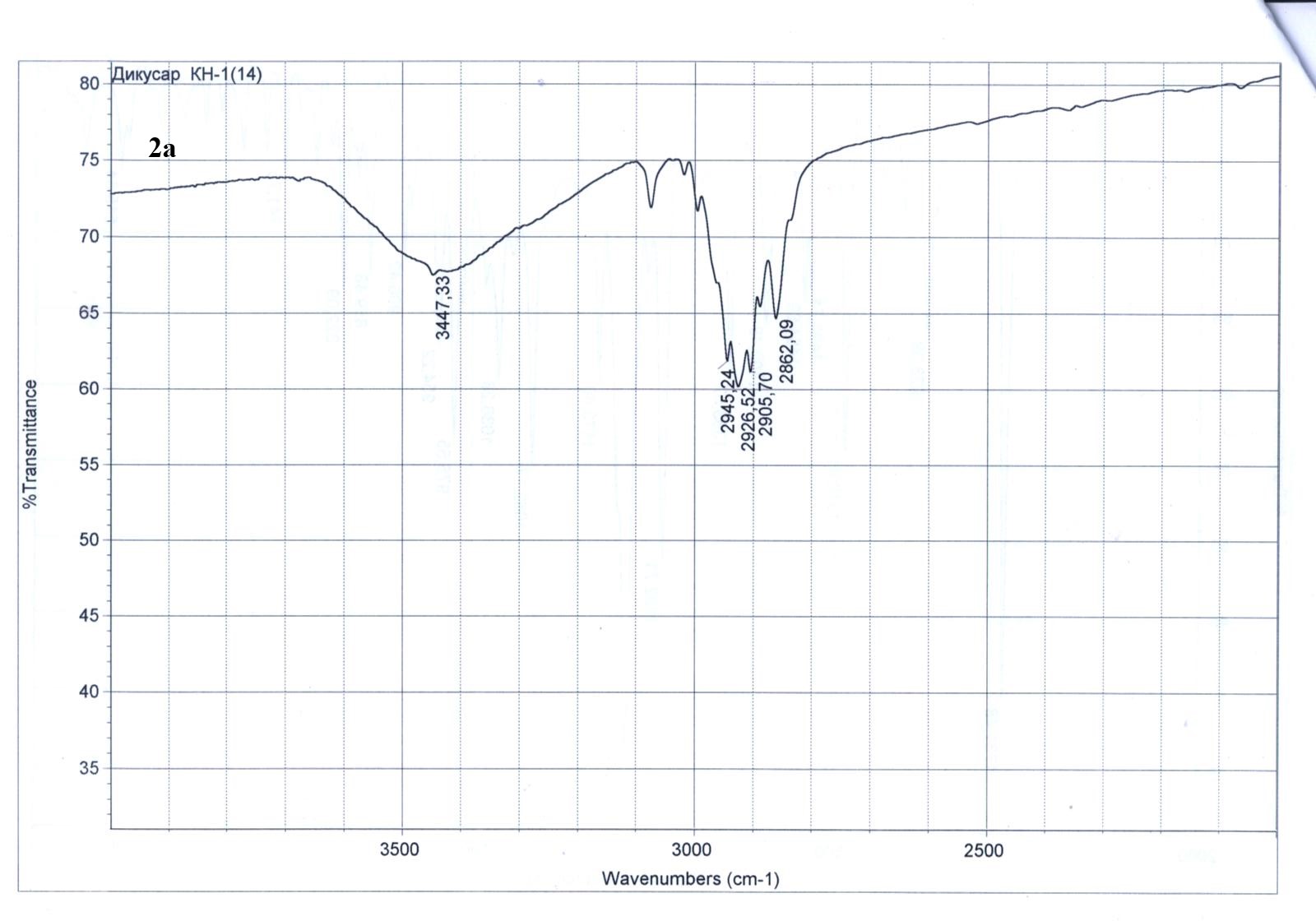

Supplement: Supplementary file 1 [file molecules-27-03476-s001.zip › IR/2a (IR2).jpg]

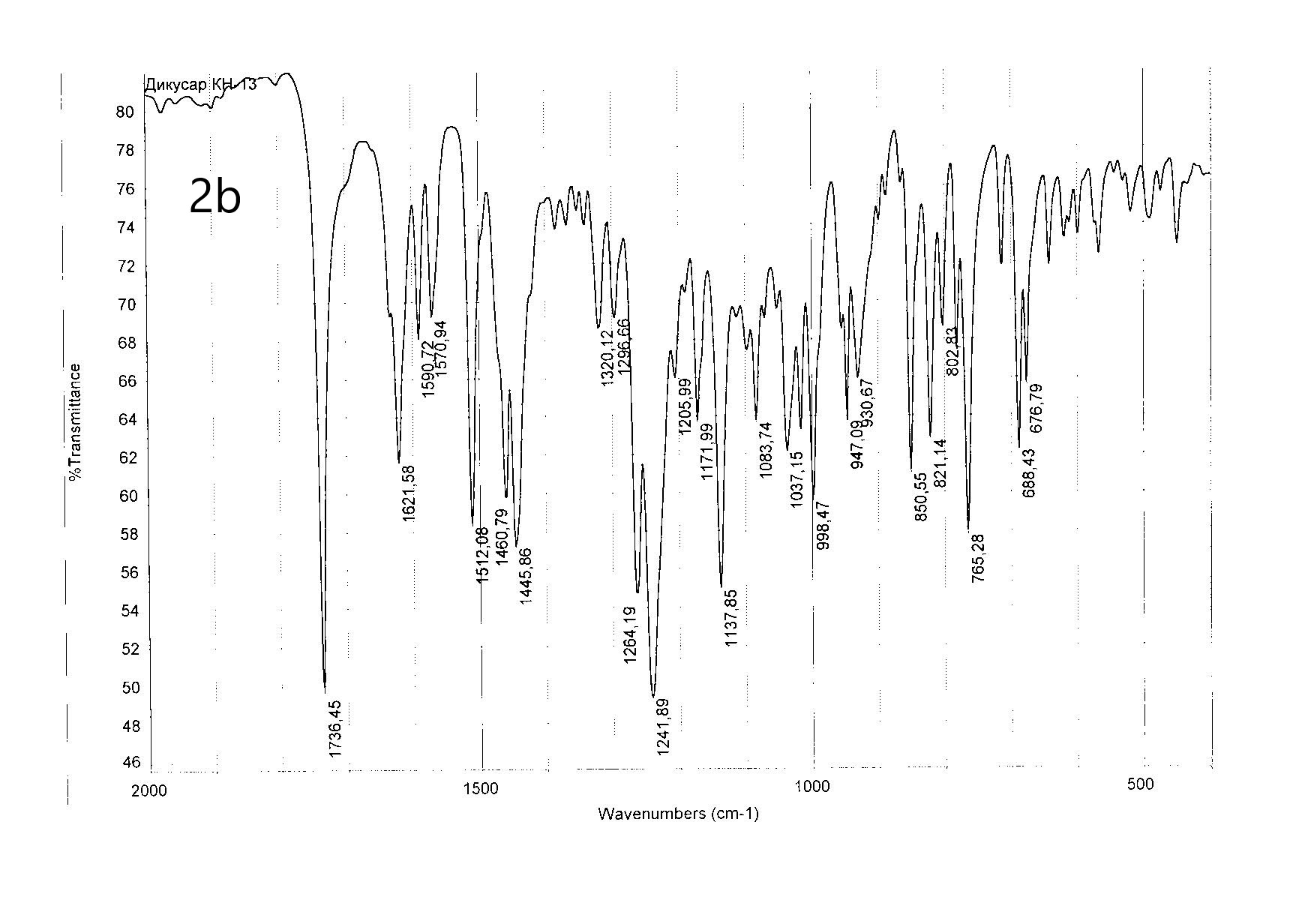

Supplement: Supplementary file 1 [file molecules-27-03476-s001.zip › IR/2b_o¬o1⁄4_1.jpg]

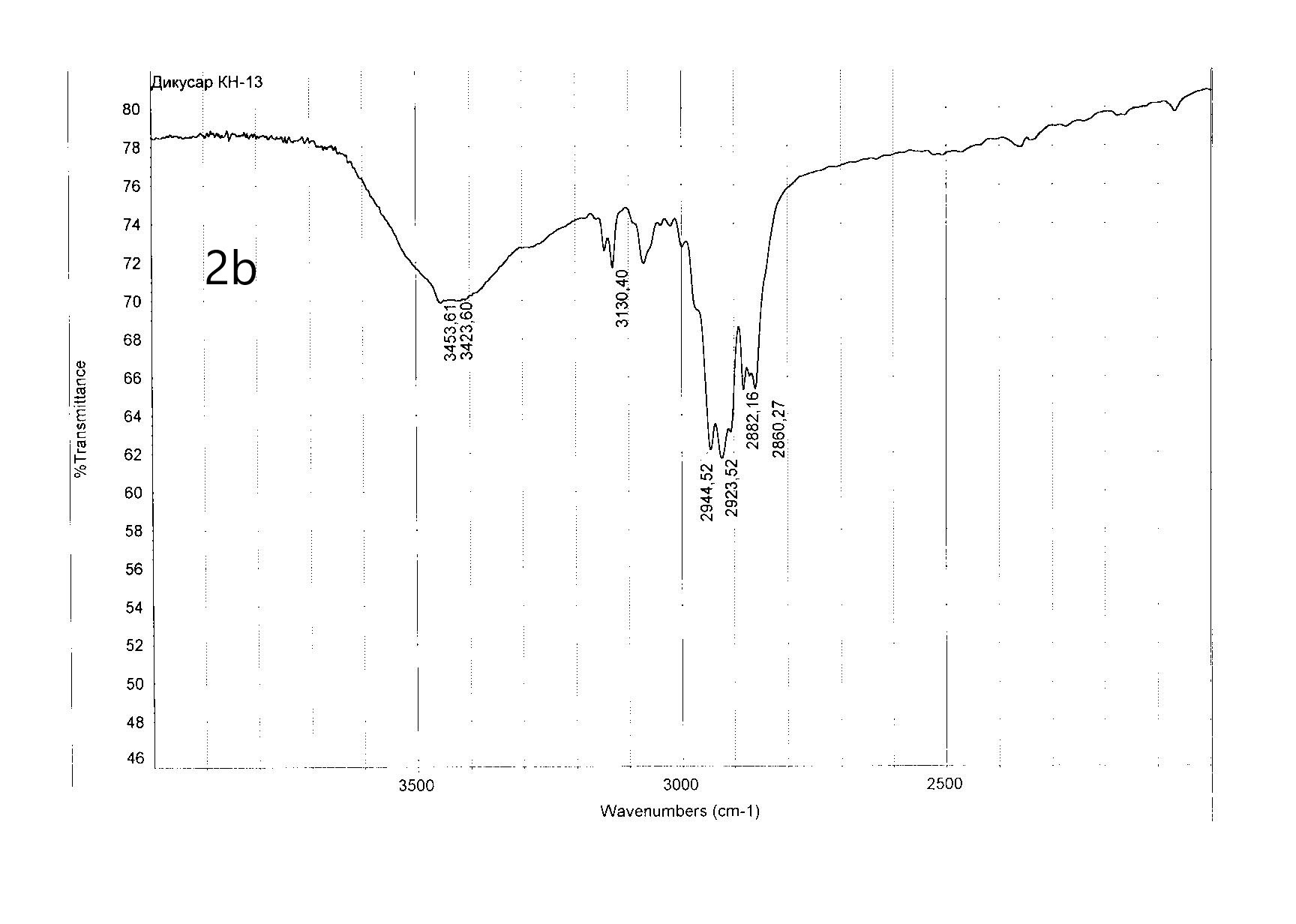

Supplement: Supplementary file 1 [file molecules-27-03476-s001.zip › IR/2b_o¬o1⁄4_2.jpg]

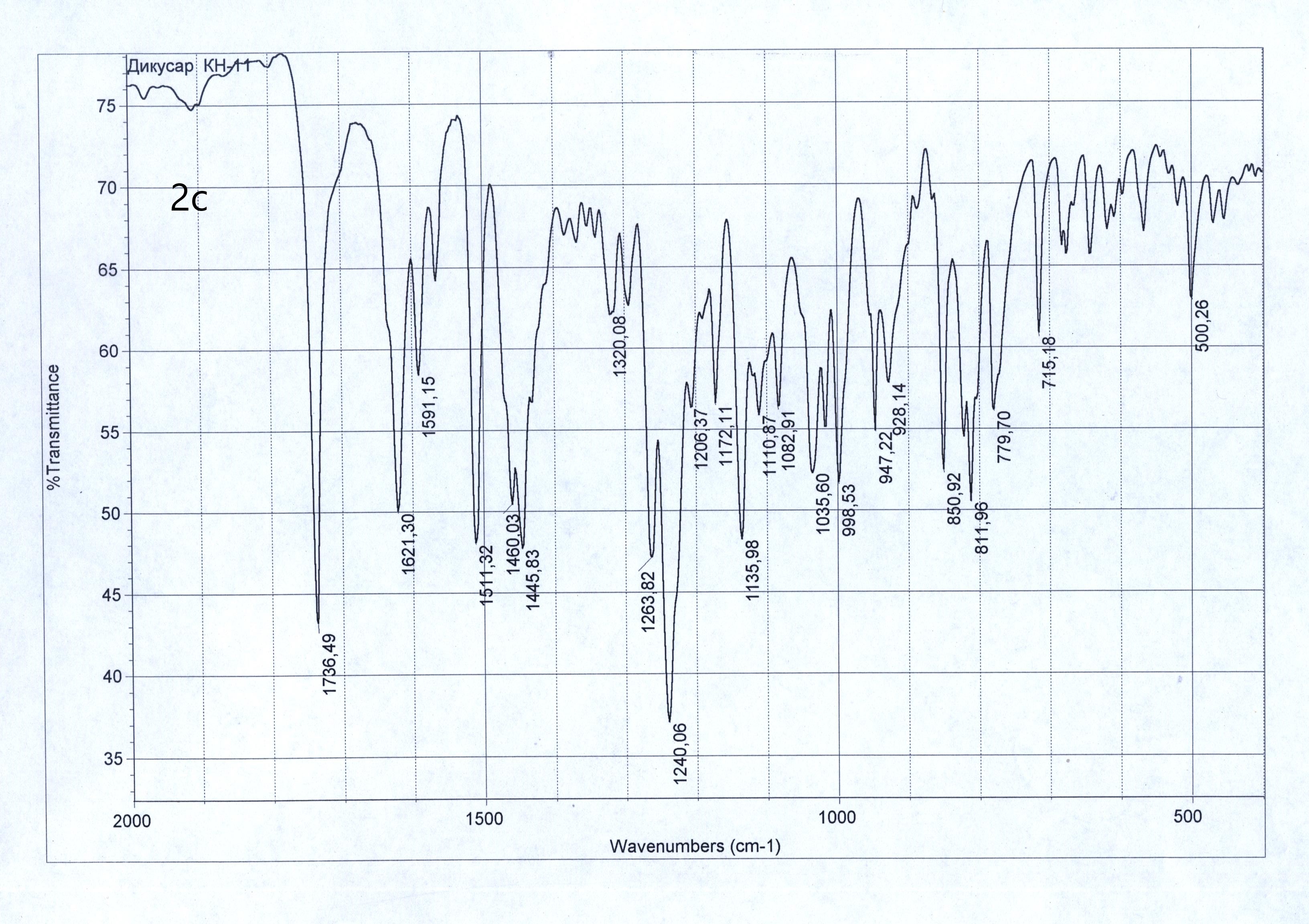

Supplement: Supplementary file 1 [file molecules-27-03476-s001.zip › IR/2c (o¬o1⁄4) 001.jpg]

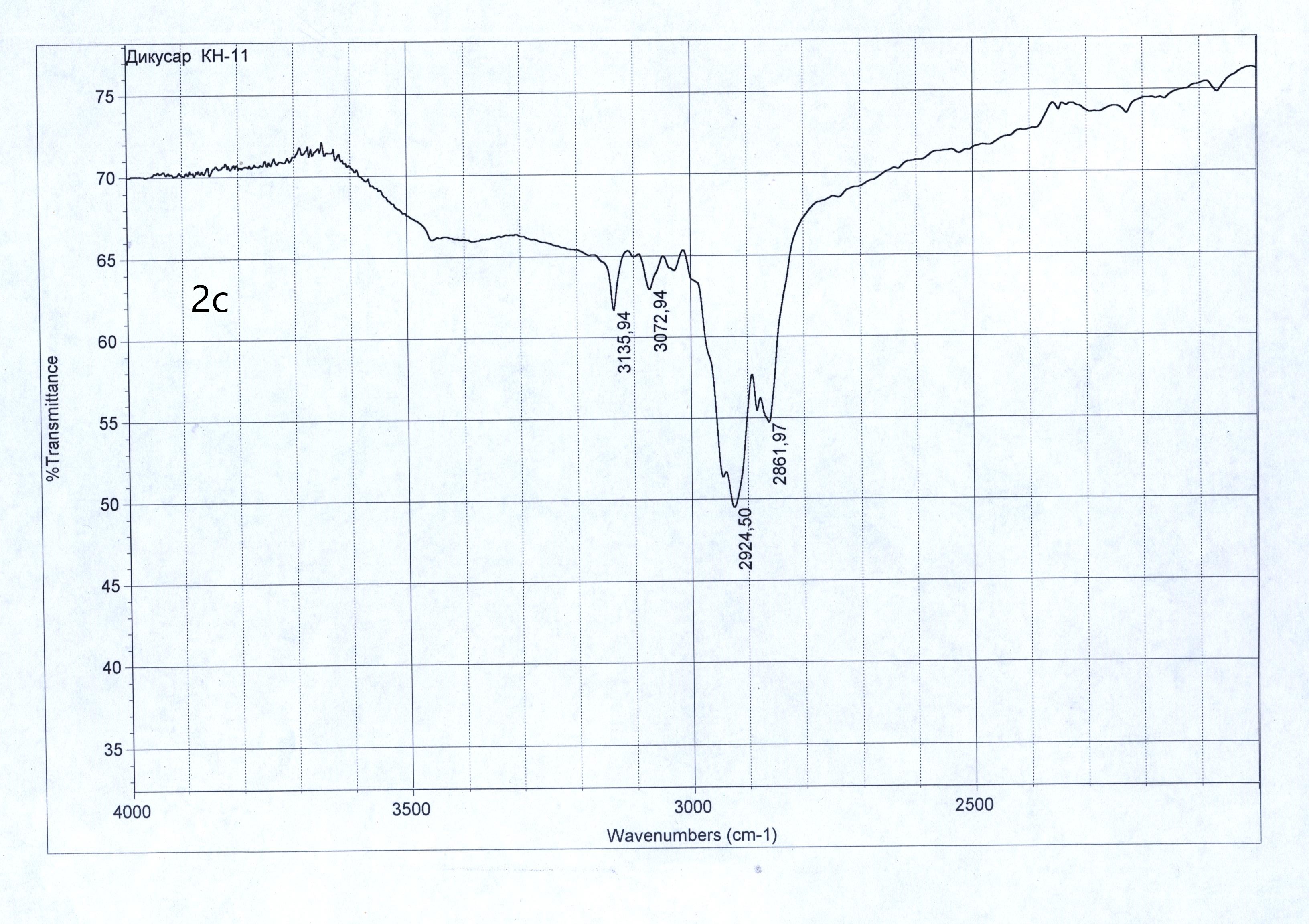

Supplement: Supplementary file 1 [file molecules-27-03476-s001.zip › IR/2c (o¬o1⁄4).jpg]

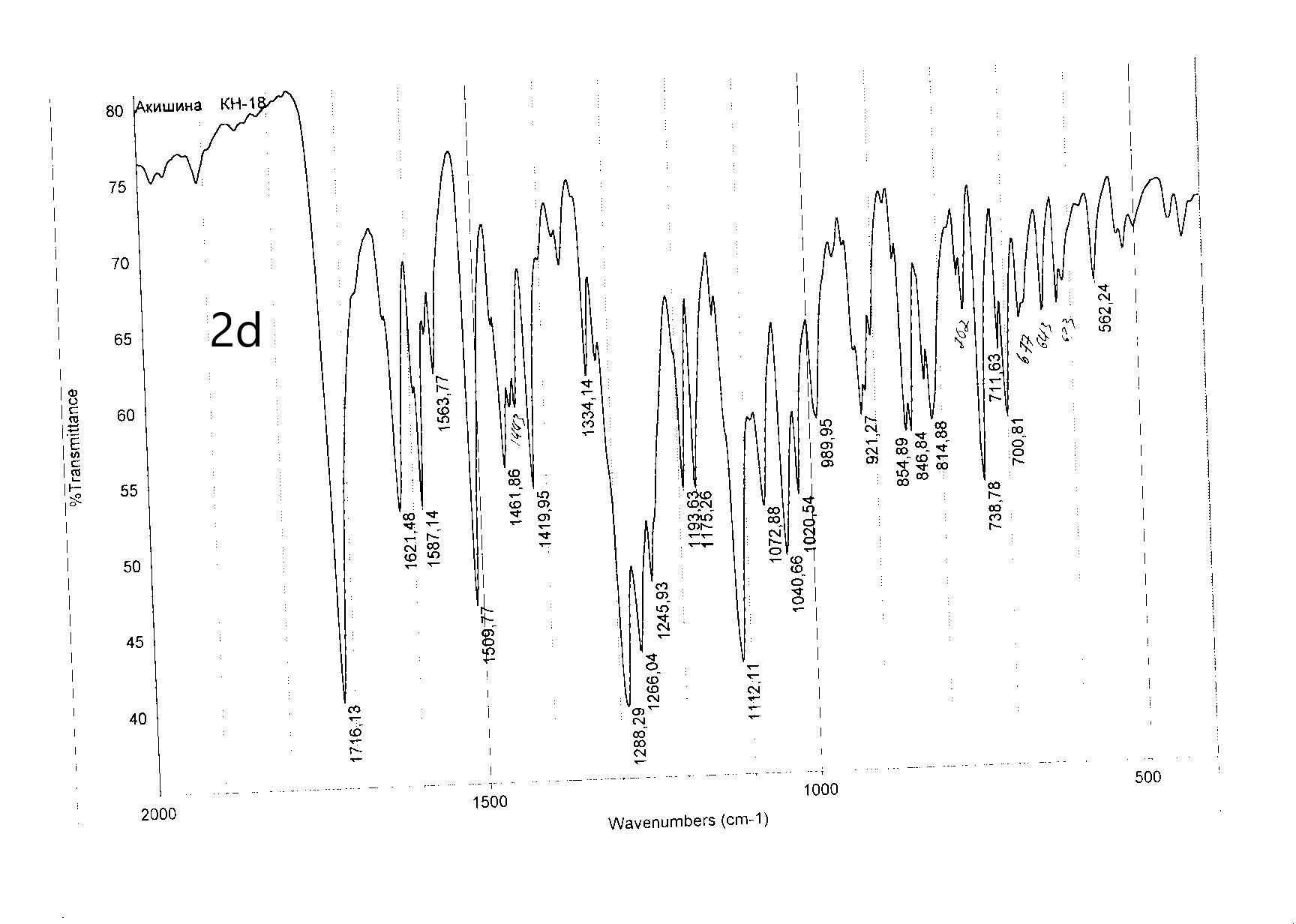

Supplement: Supplementary file 1 [file molecules-27-03476-s001.zip › IR/2d_o¬o1⁄4_1.jpg]

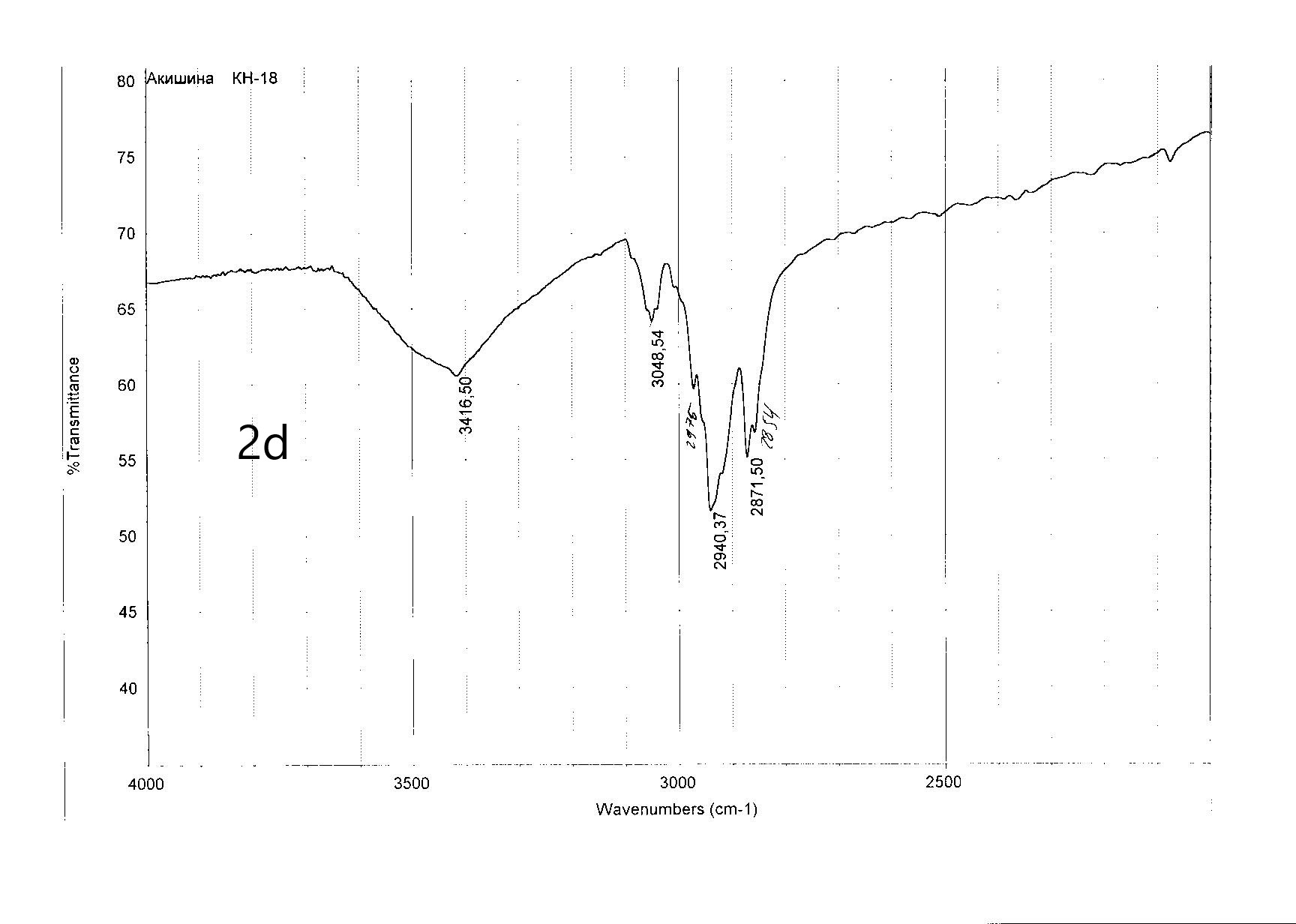

Supplement: Supplementary file 1 [file molecules-27-03476-s001.zip › IR/2d-_o¬o1⁄4_2.jpg]

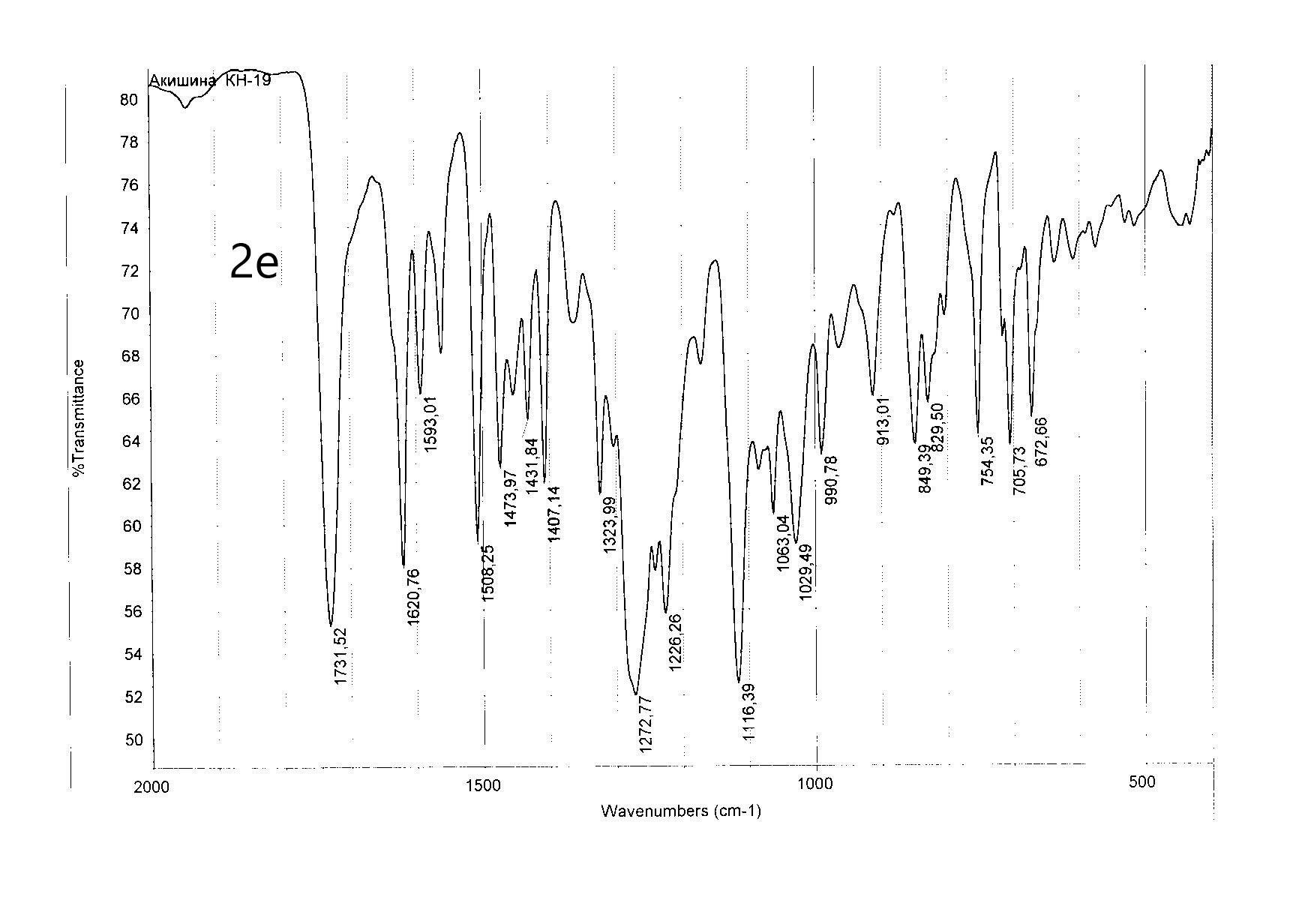

Supplement: Supplementary file 1 [file molecules-27-03476-s001.zip › IR/2e_o¬o1⁄4_1.jpg]

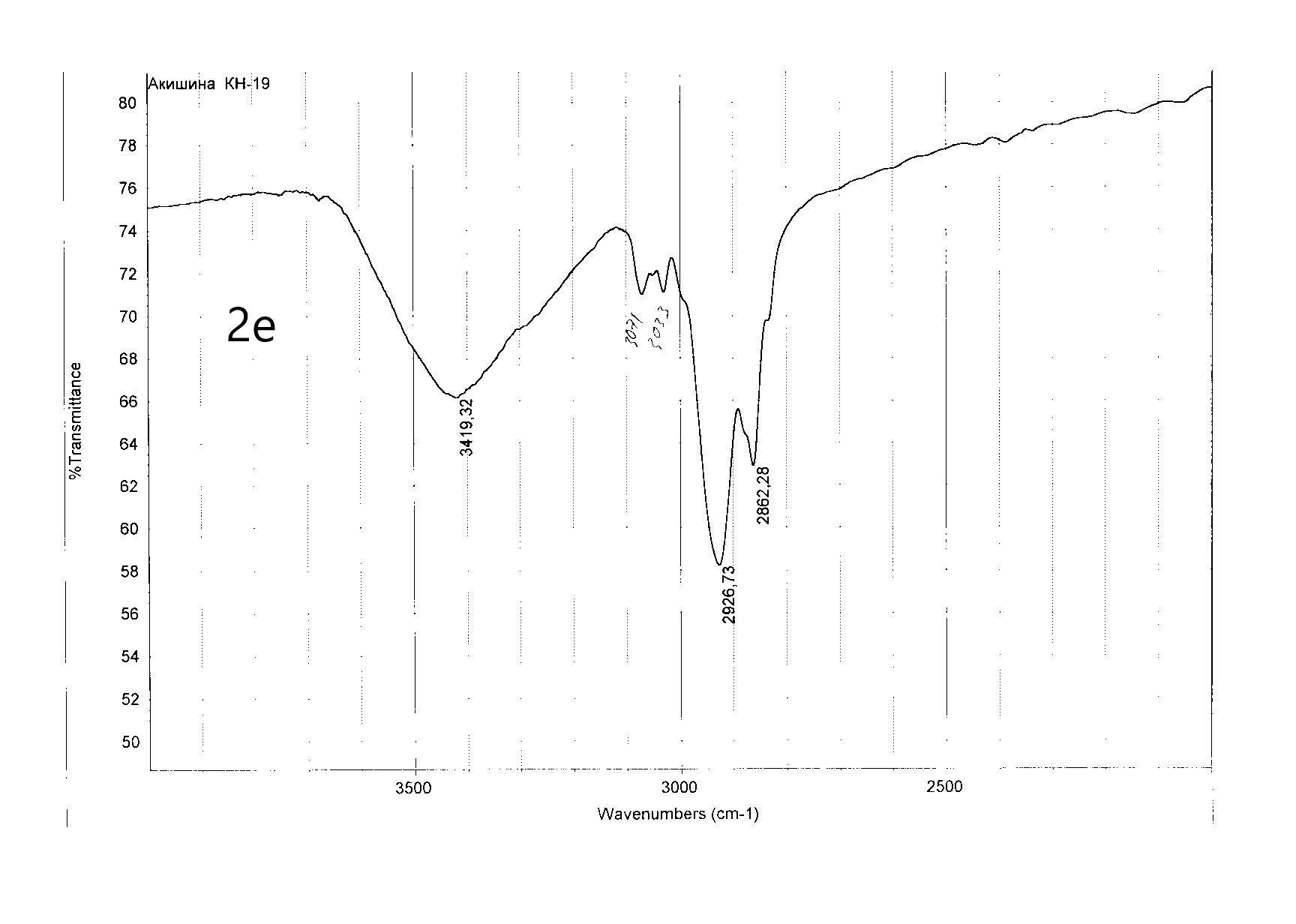

Supplement: Supplementary file 1 [file molecules-27-03476-s001.zip › IR/2e_o¬o1⁄4_2.jpg]

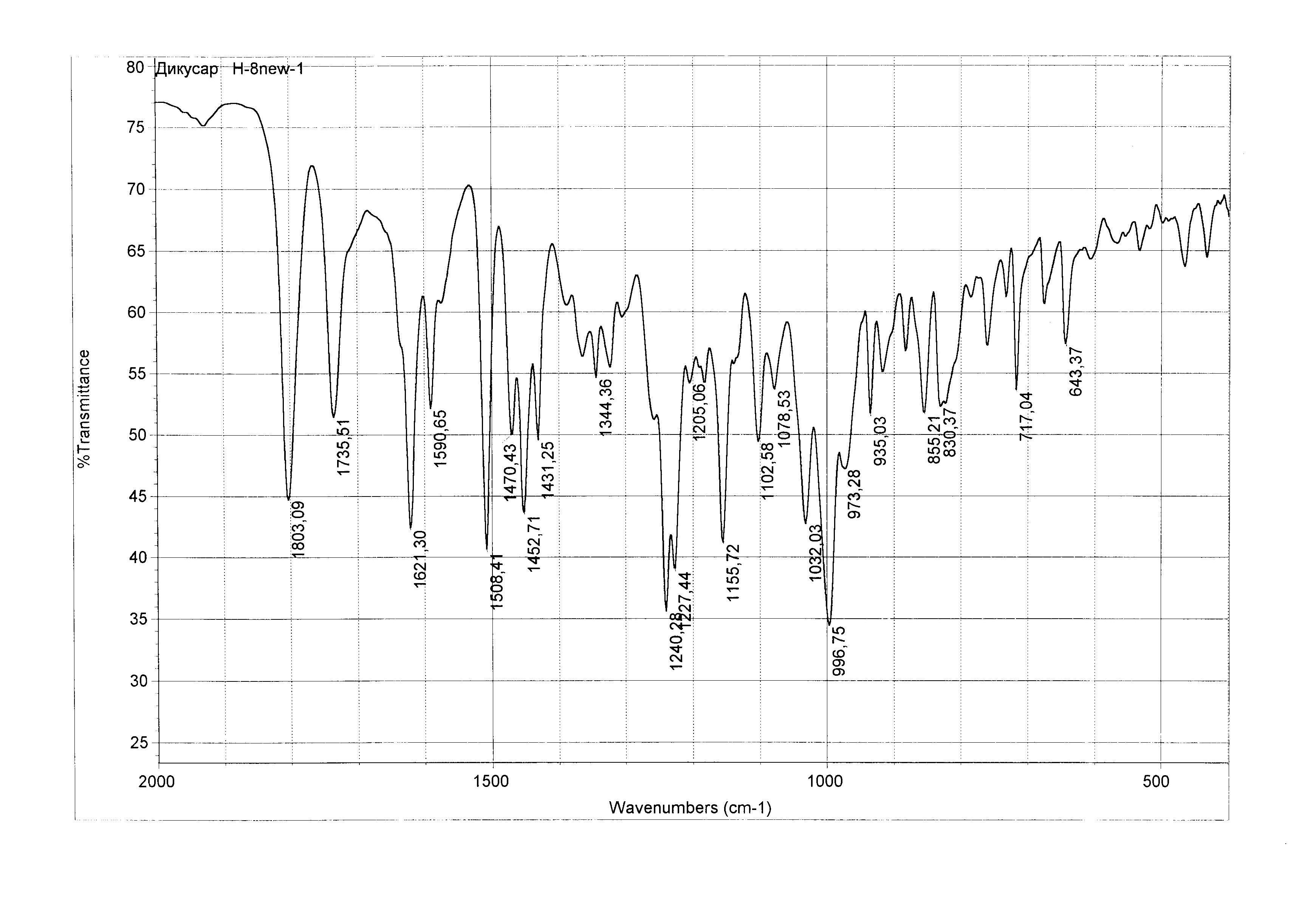

Supplement: Supplementary file 1 [file molecules-27-03476-s001.zip › IR/2f_o¬o1⁄4_1.jpg]

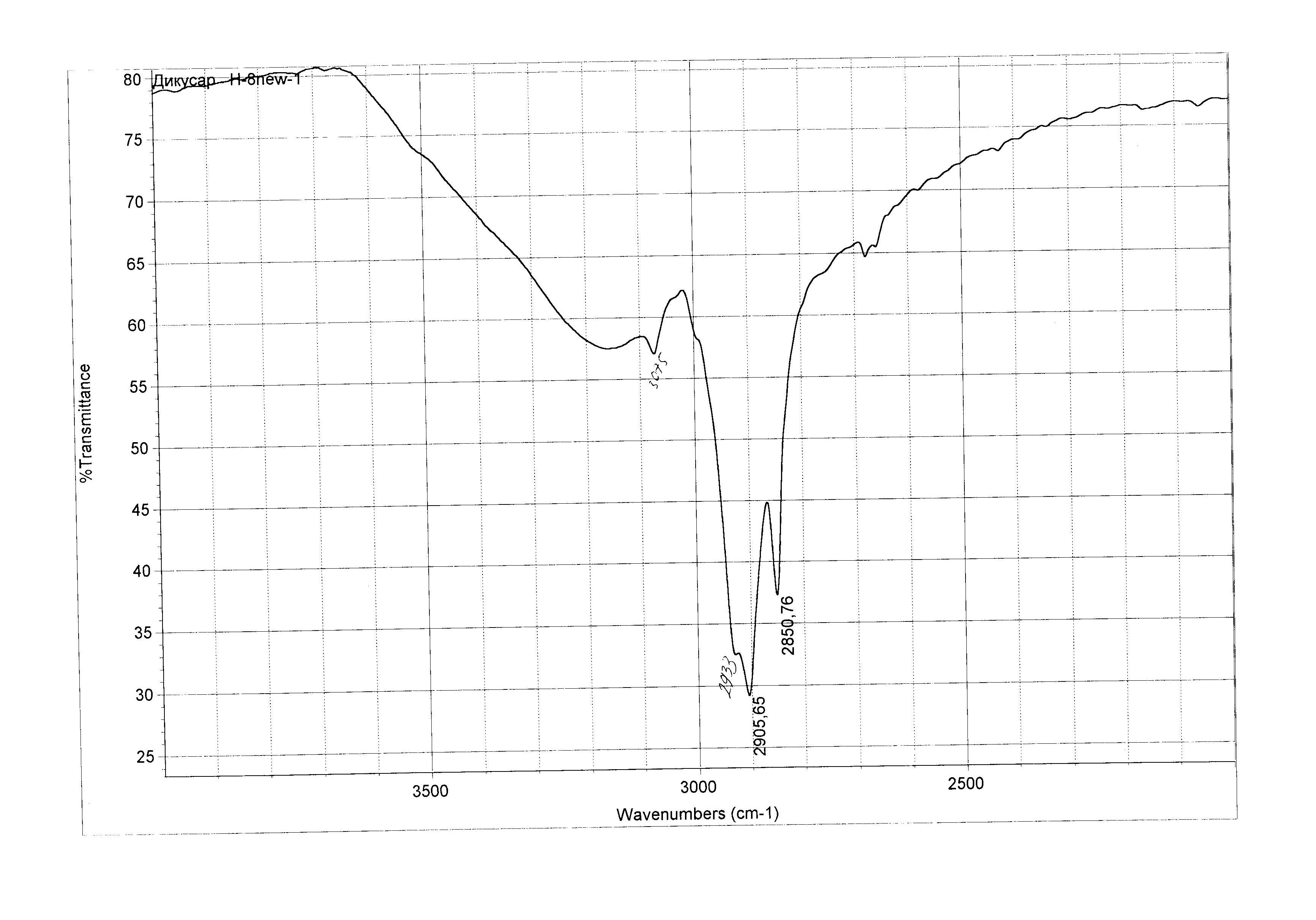

Supplement: Supplementary file 1 [file molecules-27-03476-s001.zip › IR/2f_o¬o1⁄4_2.jpg]

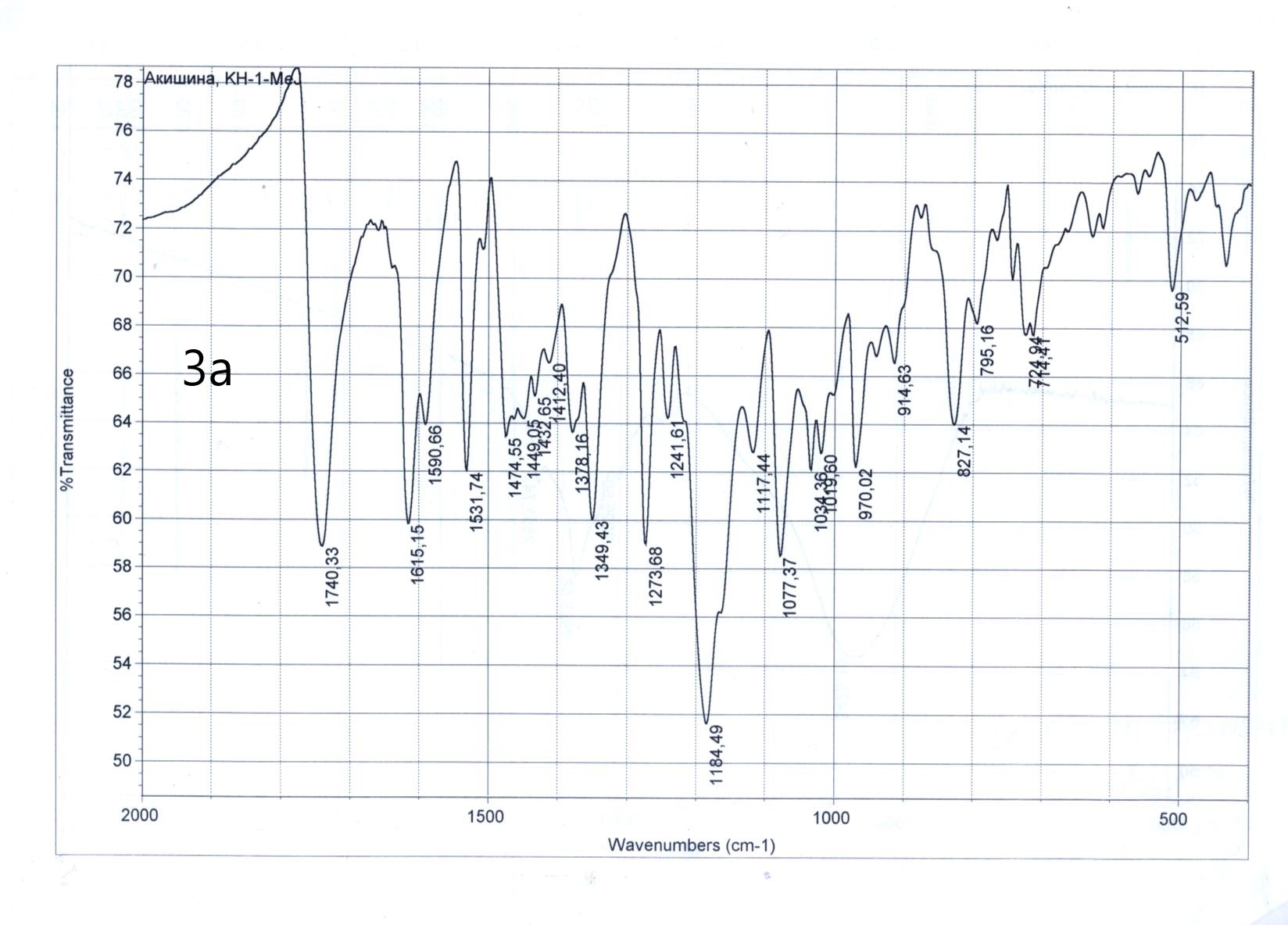

Supplement: Supplementary file 1 [file molecules-27-03476-s001.zip › IR/3a (o¬o1⁄4-1).jpg]

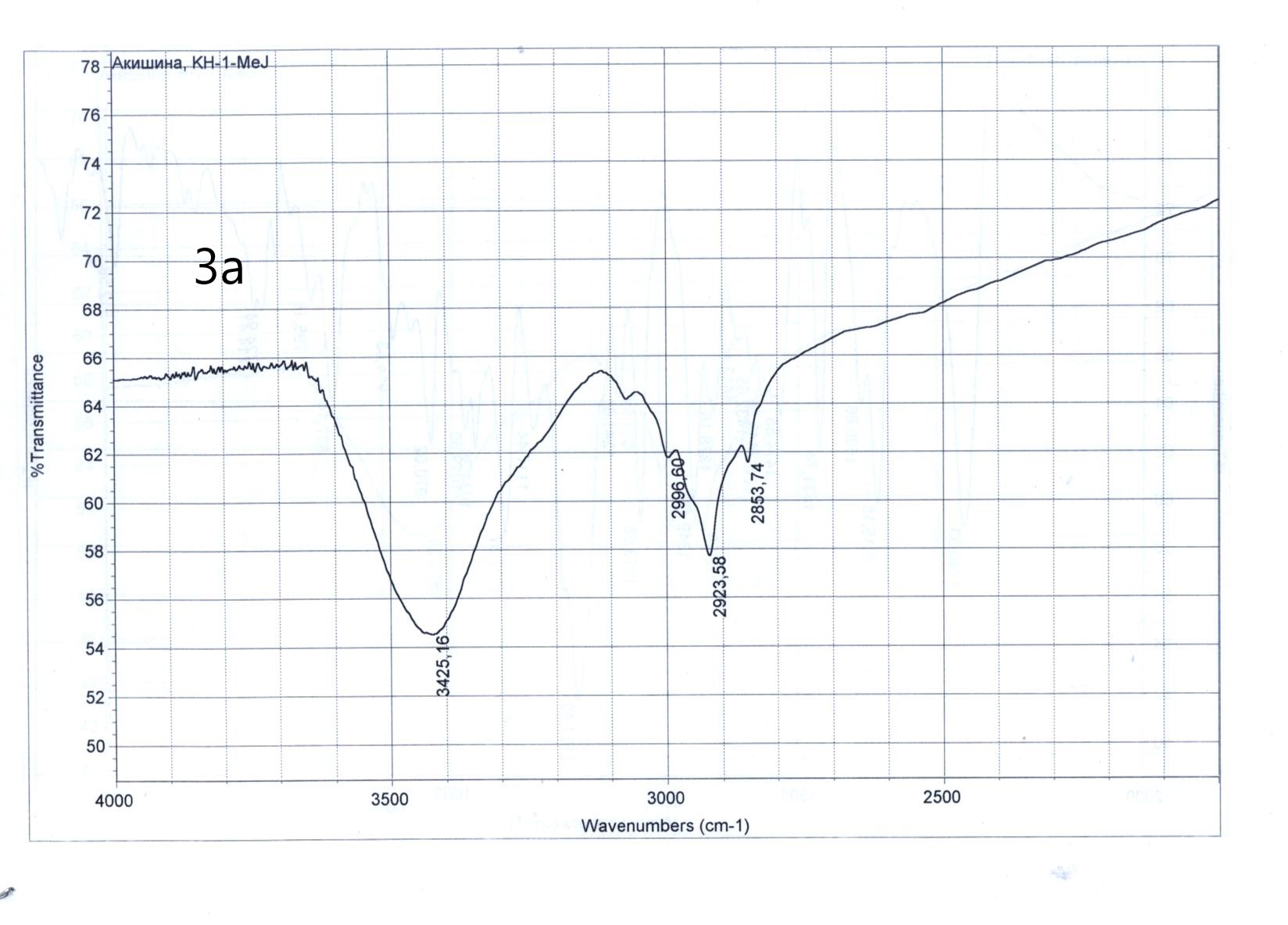

Supplement: Supplementary file 1 [file molecules-27-03476-s001.zip › IR/3a (o¬o1⁄4-2).jpg]

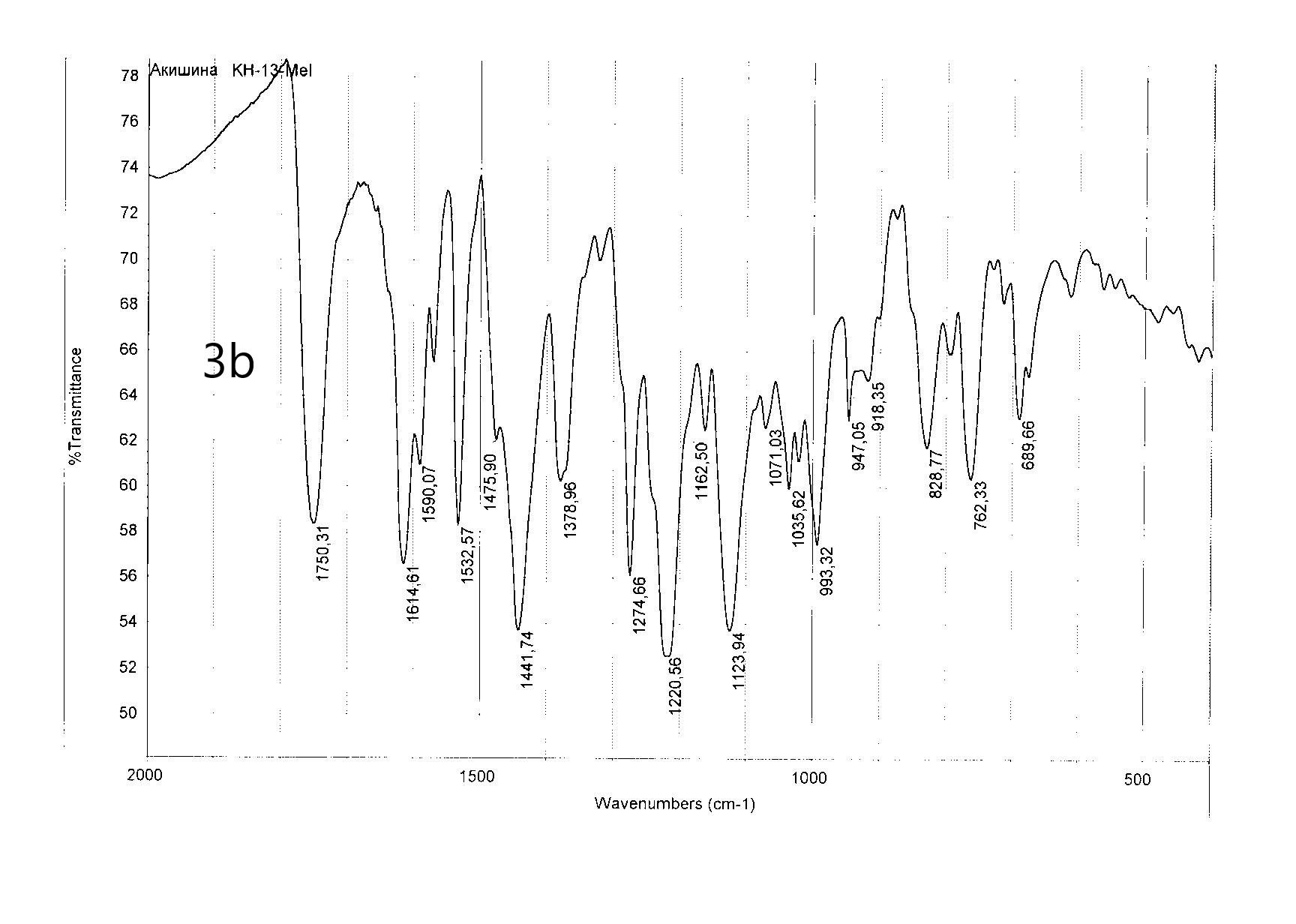

Supplement: Supplementary file 1 [file molecules-27-03476-s001.zip › IR/3b_o¬o1⁄4_1.jpg]

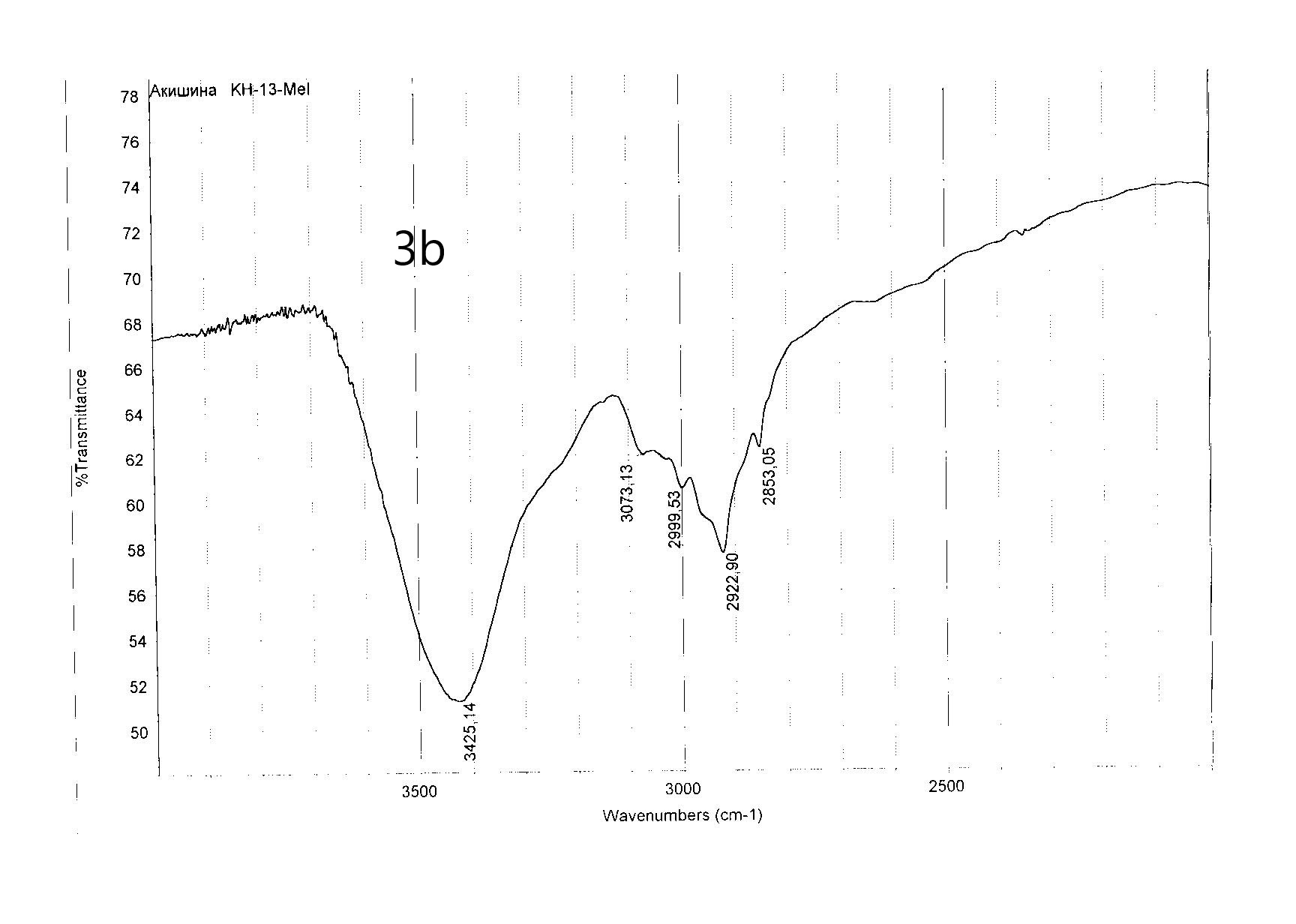

Supplement: Supplementary file 1 [file molecules-27-03476-s001.zip › IR/3b_o¬o1⁄4_2.jpg]

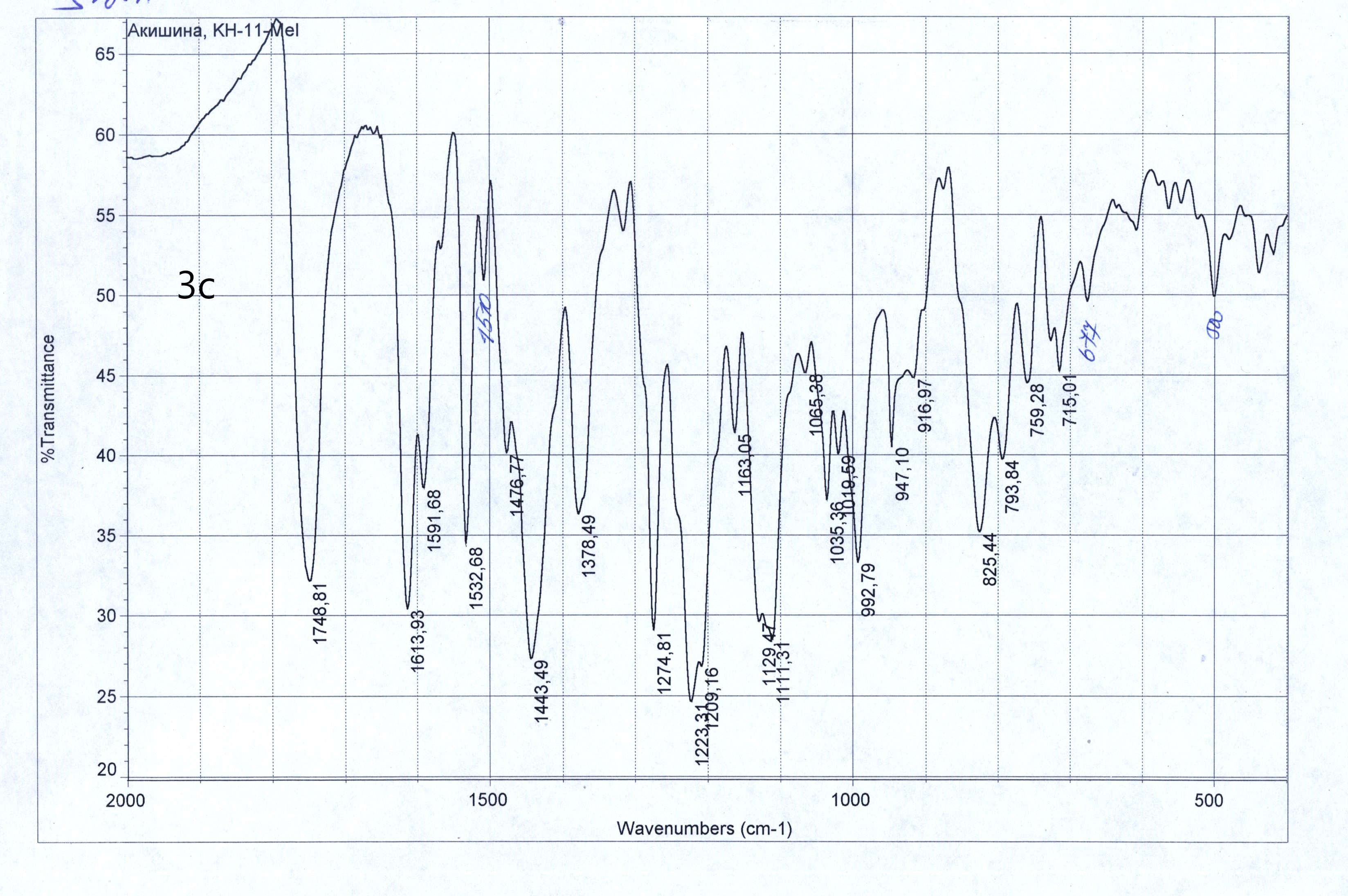

Supplement: Supplementary file 1 [file molecules-27-03476-s001.zip › IR/3c (o¬o1⁄4) 001.jpg]

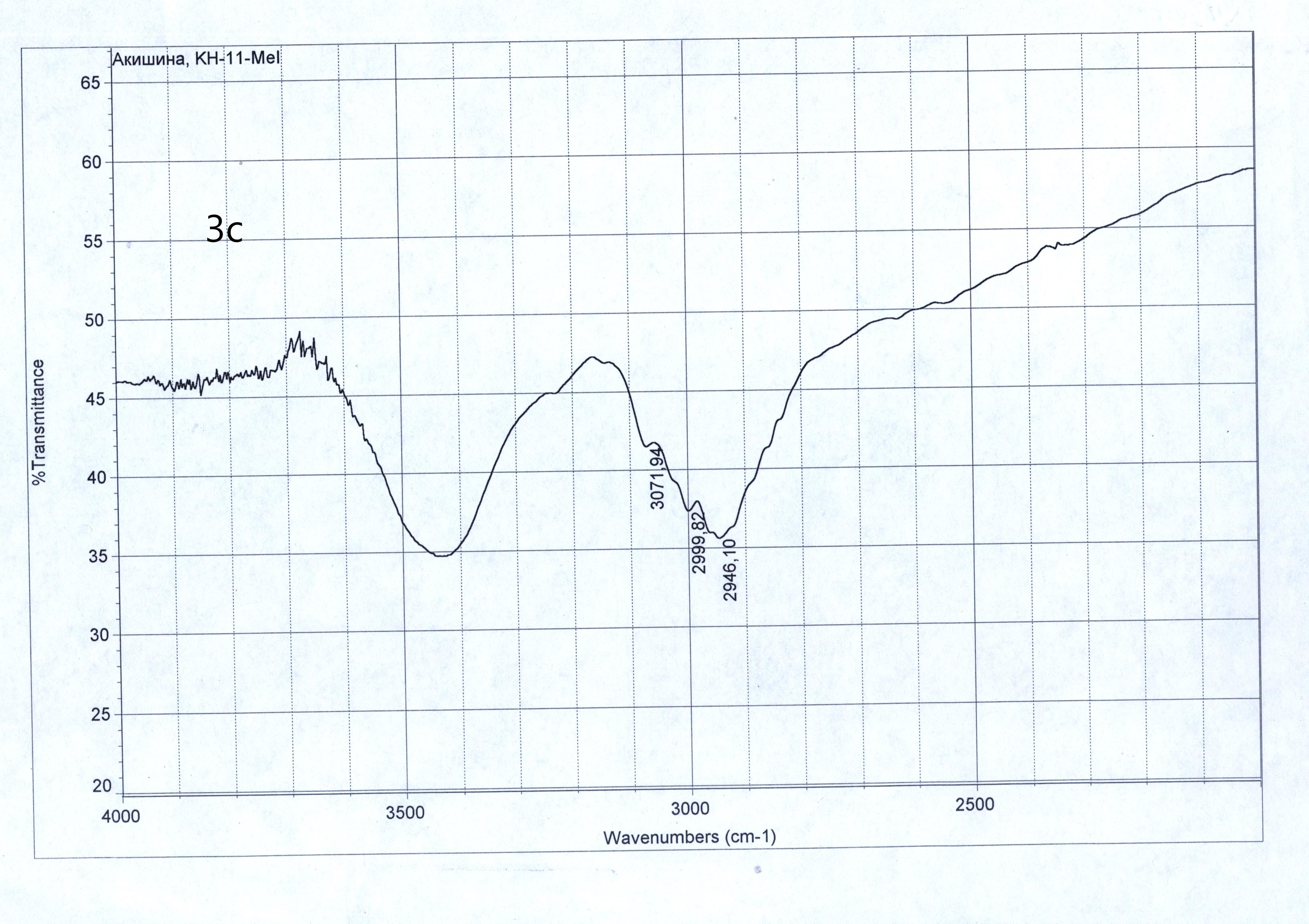

Supplement: Supplementary file 1 [file molecules-27-03476-s001.zip › IR/3c (o¬o1⁄4).jpg]

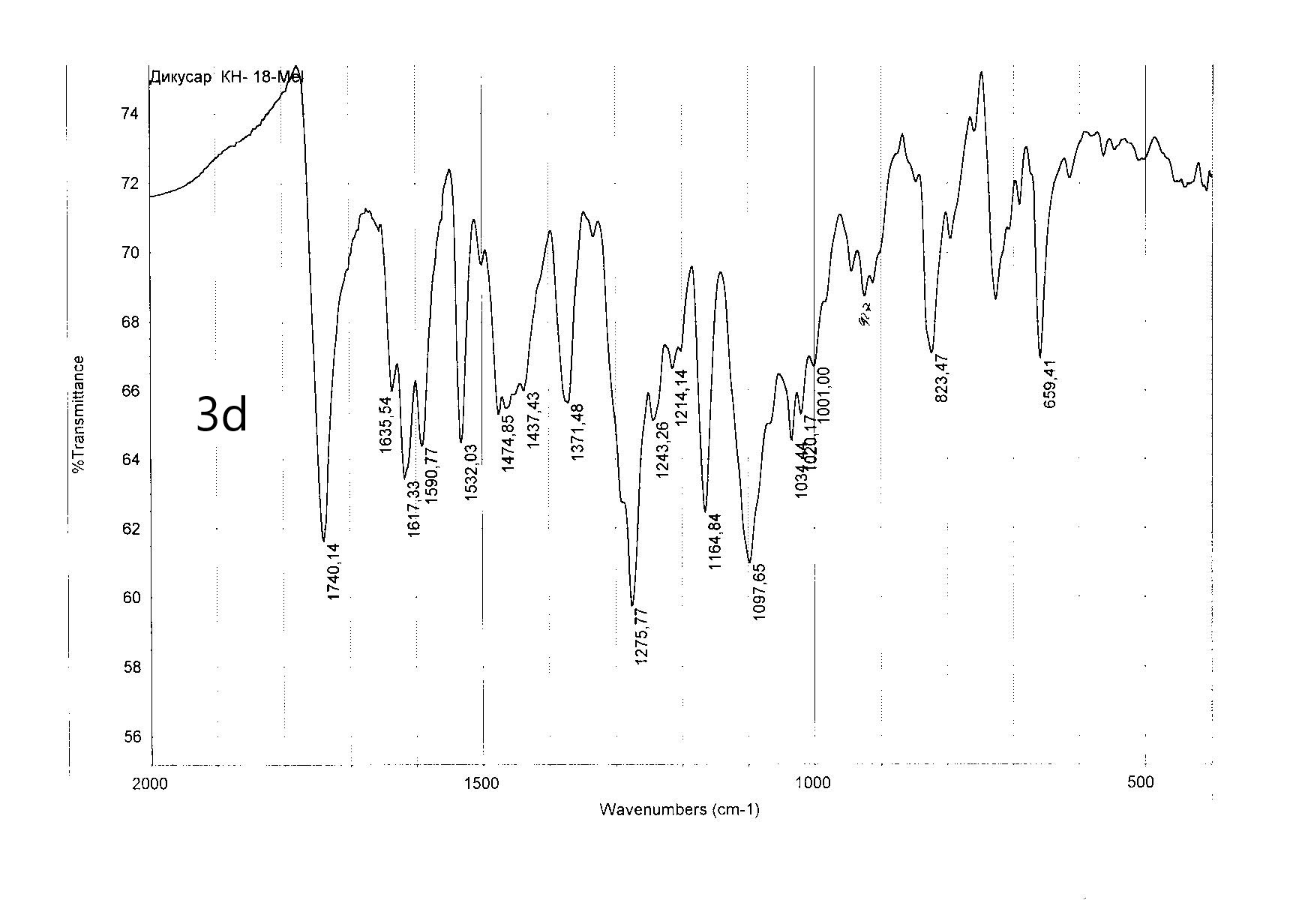

Supplement: Supplementary file 1 [file molecules-27-03476-s001.zip › IR/3d_o¬o1⁄4_1.jpg]

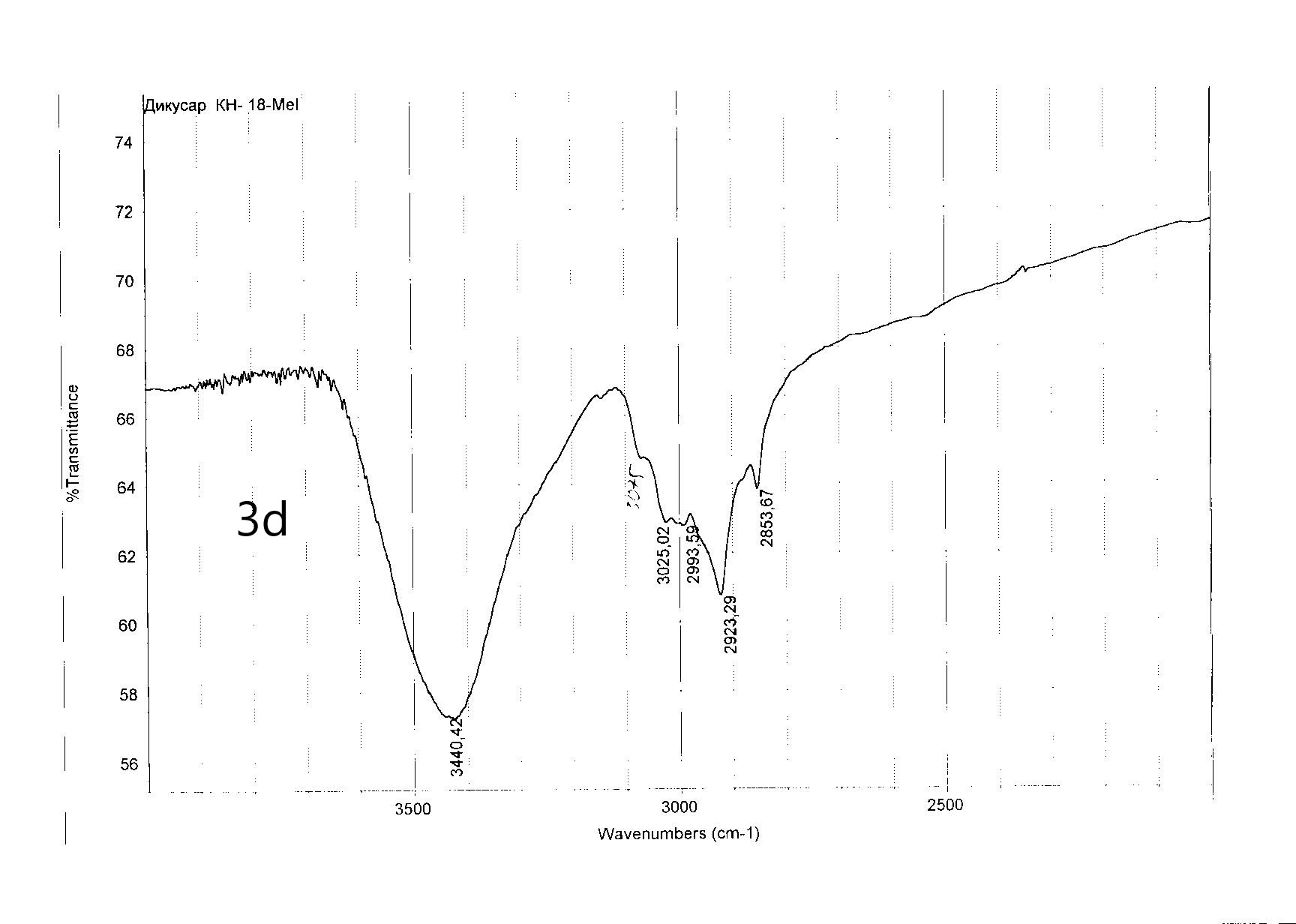

Supplement: Supplementary file 1 [file molecules-27-03476-s001.zip › IR/3d_o¬o1⁄4_2.jpg]

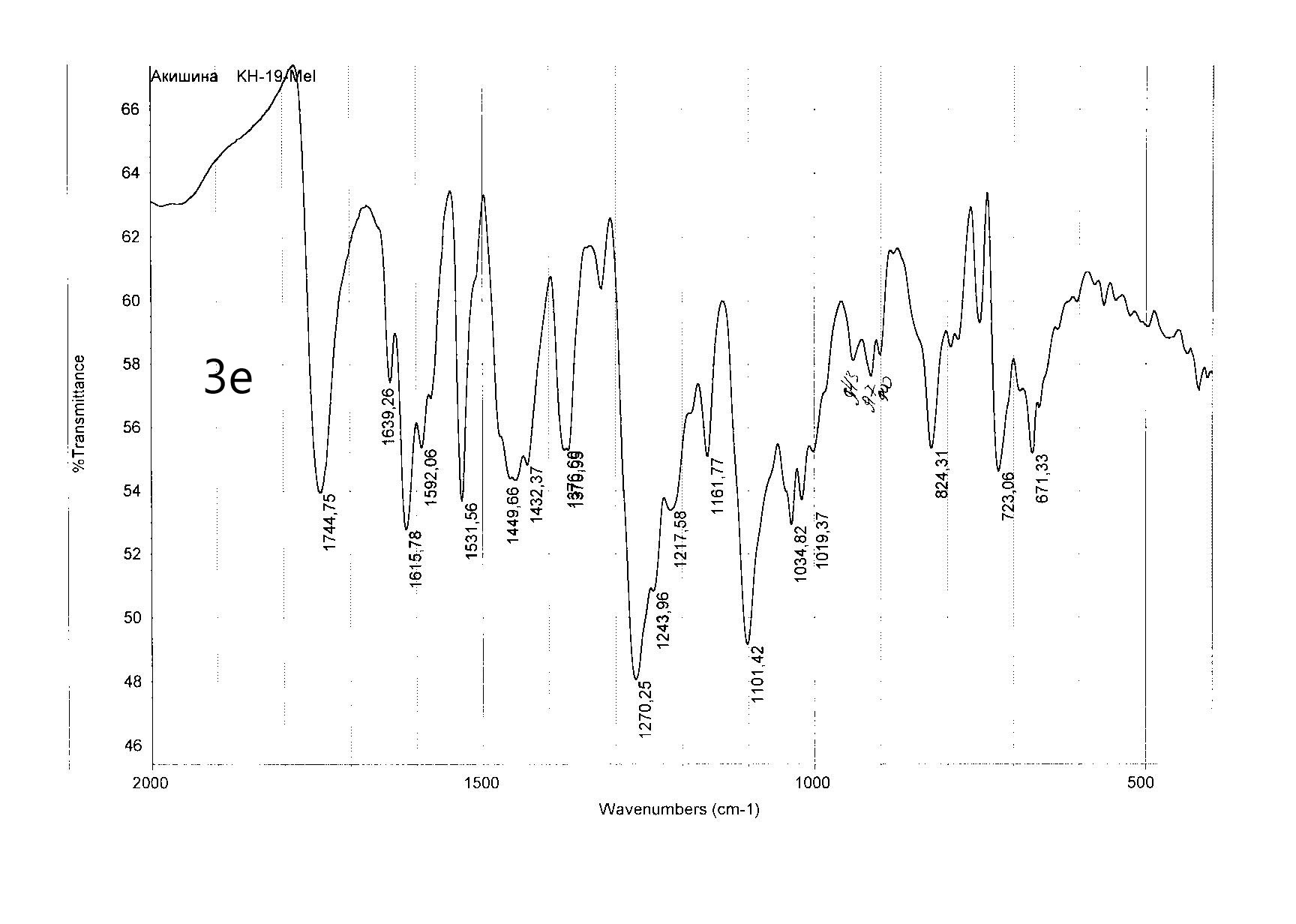

Supplement: Supplementary file 1 [file molecules-27-03476-s001.zip › IR/3e_o¬o1⁄4_1.jpg]

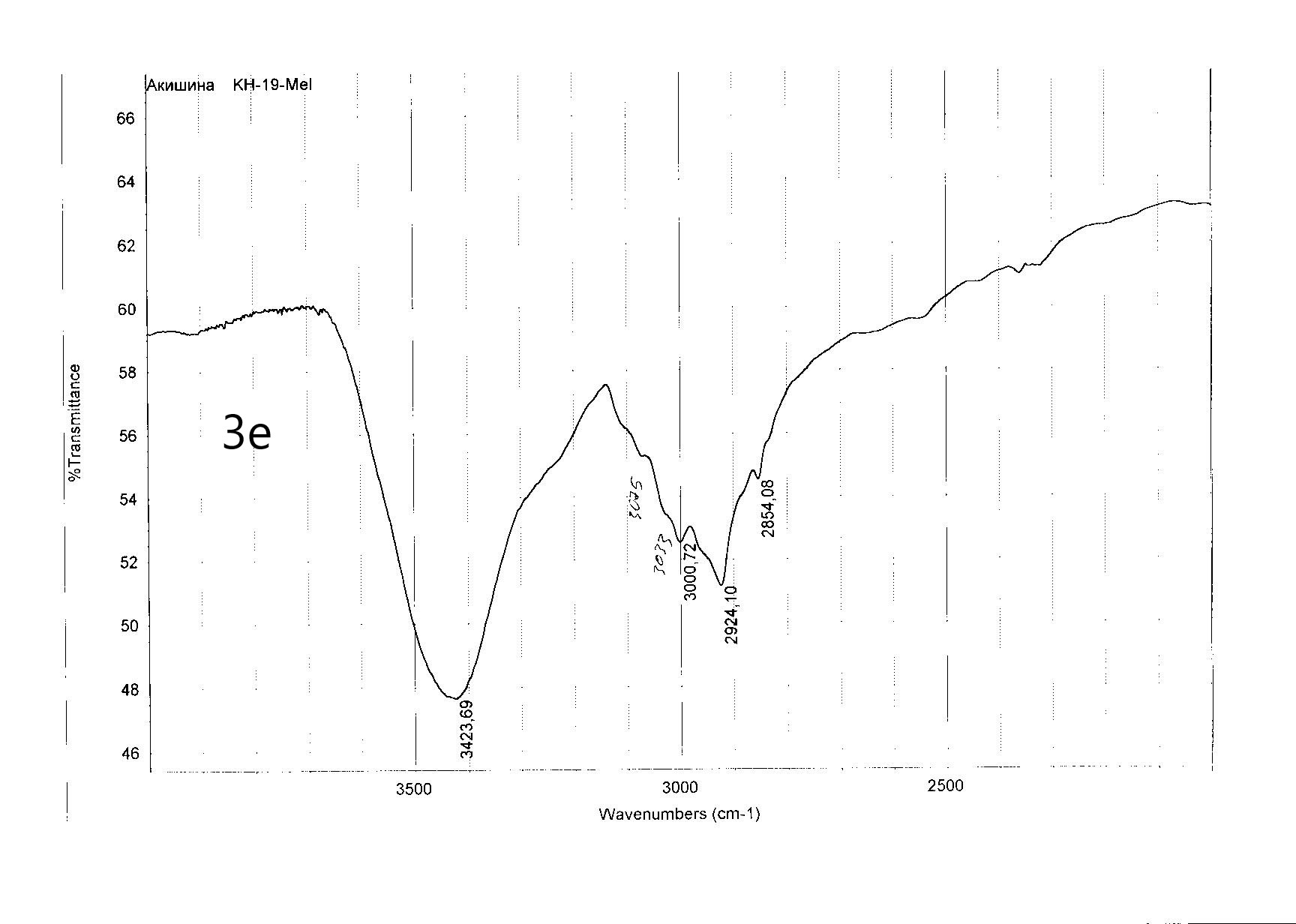

Supplement: Supplementary file 1 [file molecules-27-03476-s001.zip › IR/3e_o¬o1⁄4_2.jpg]

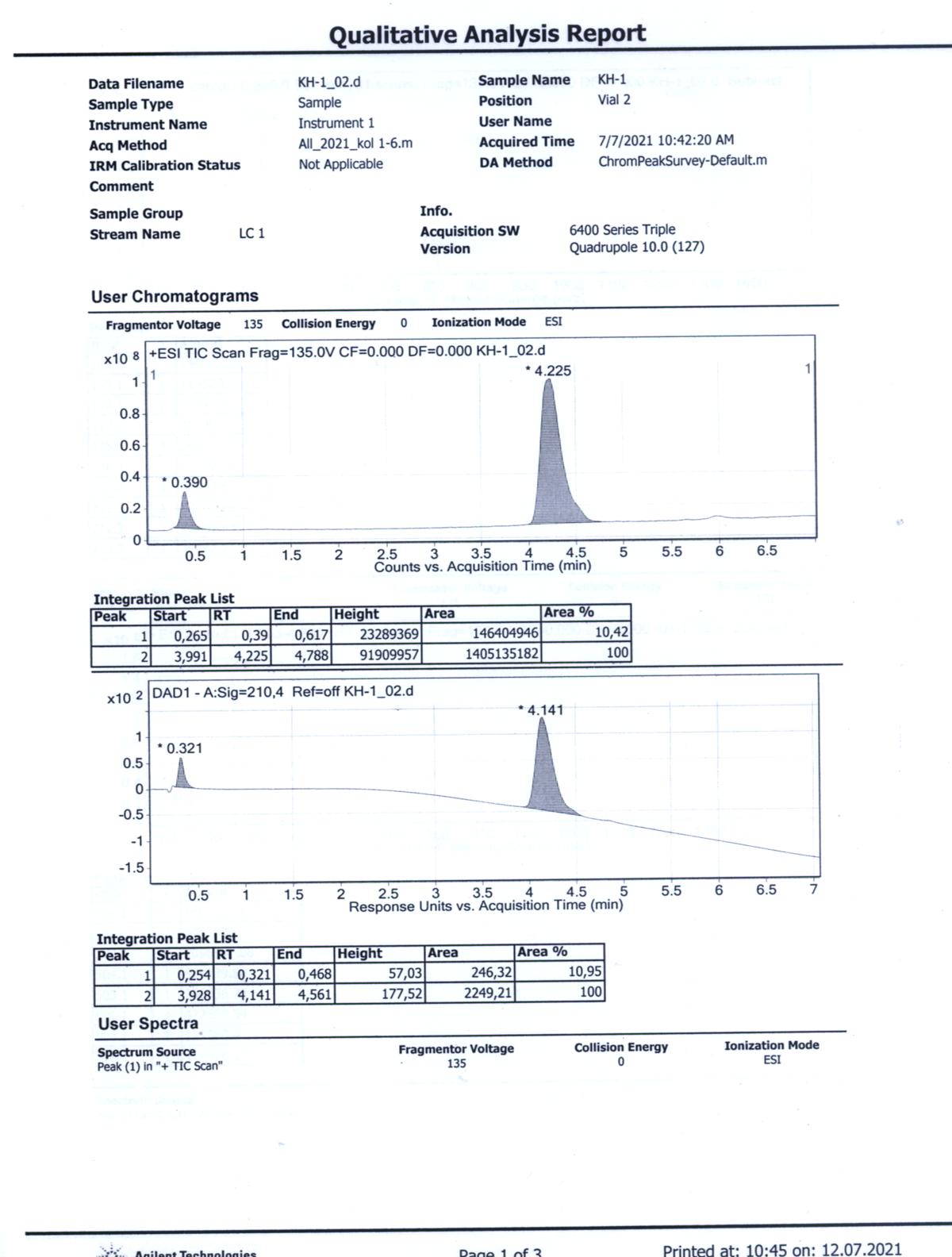

Supplement: Supplementary file 1 [file molecules-27-03476-s001.zip › MS/2a (mass-1).jpg]

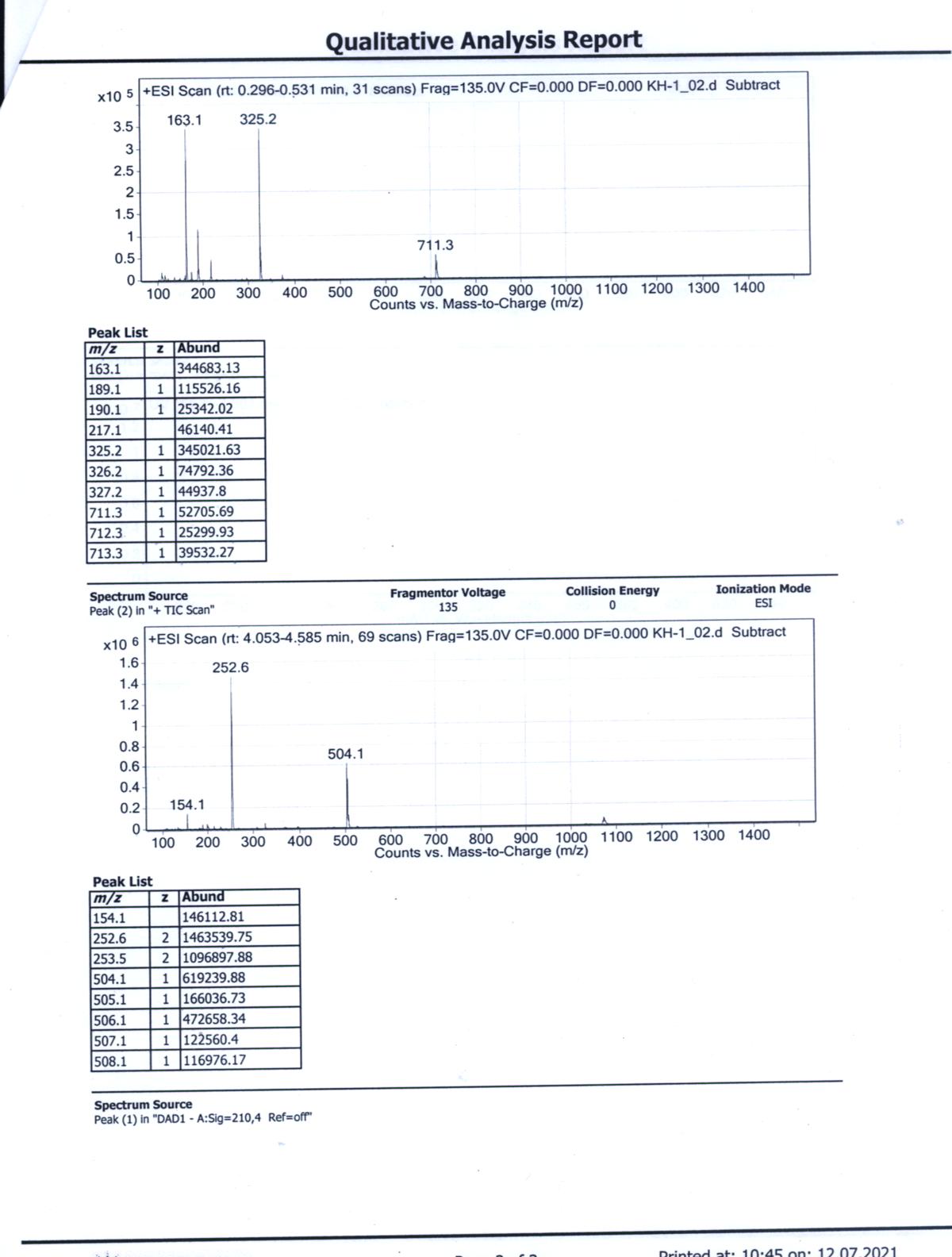

Supplement: Supplementary file 1 [file molecules-27-03476-s001.zip › MS/2a (mass-2).jpg]

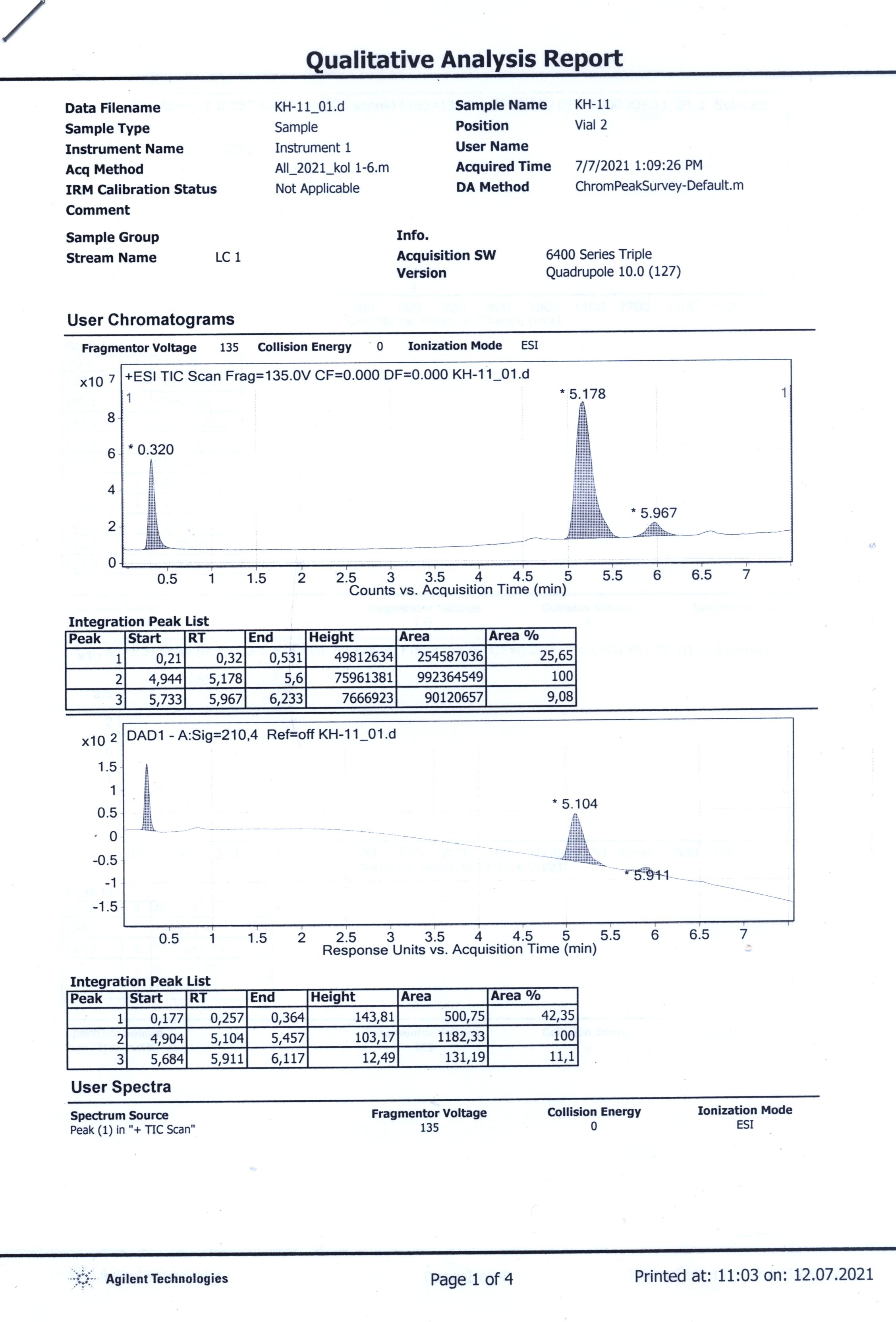

Supplement: Supplementary file 1 [file molecules-27-03476-s001.zip › MS/2c (mass1).jpg]

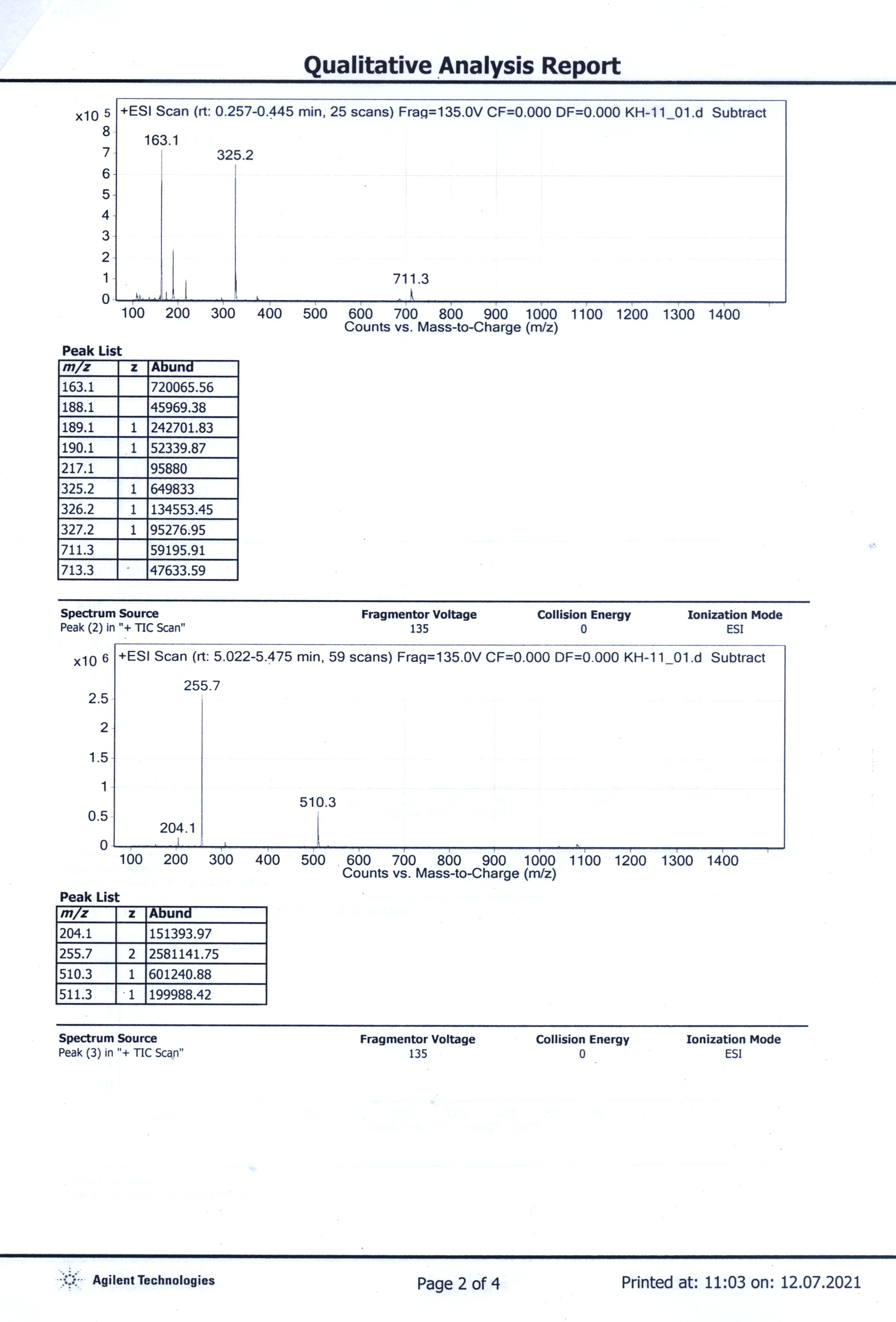

Supplement: Supplementary file 1 [file molecules-27-03476-s001.zip › MS/2c (mass2).jpg]

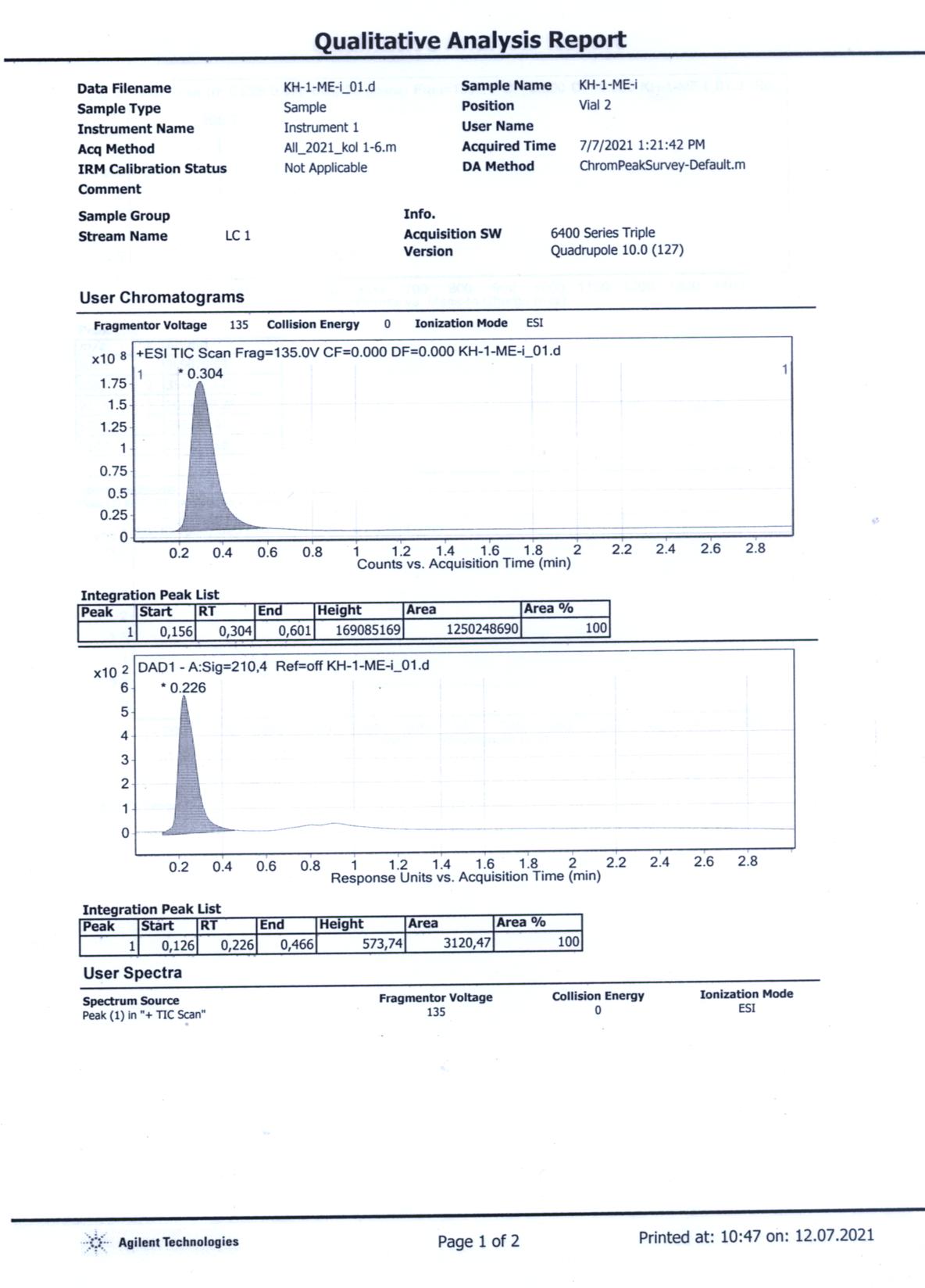

Supplement: Supplementary file 1 [file molecules-27-03476-s001.zip › MS/3a (mass-1).jpg]

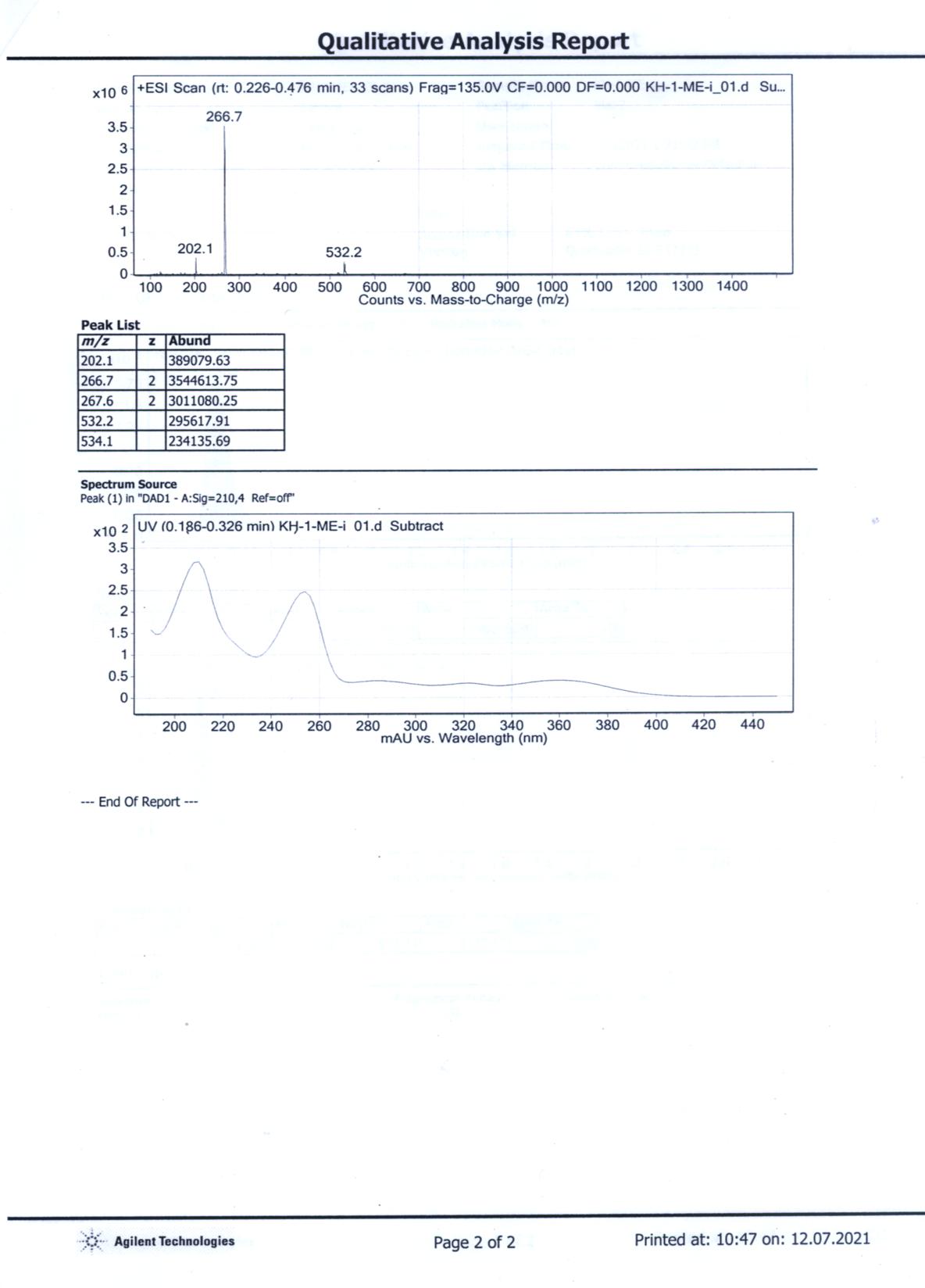

Supplement: Supplementary file 1 [file molecules-27-03476-s001.zip › MS/3a (mass-2).jpg]

**2a <sup>1</sup>H**  
**in DMSO-d6**

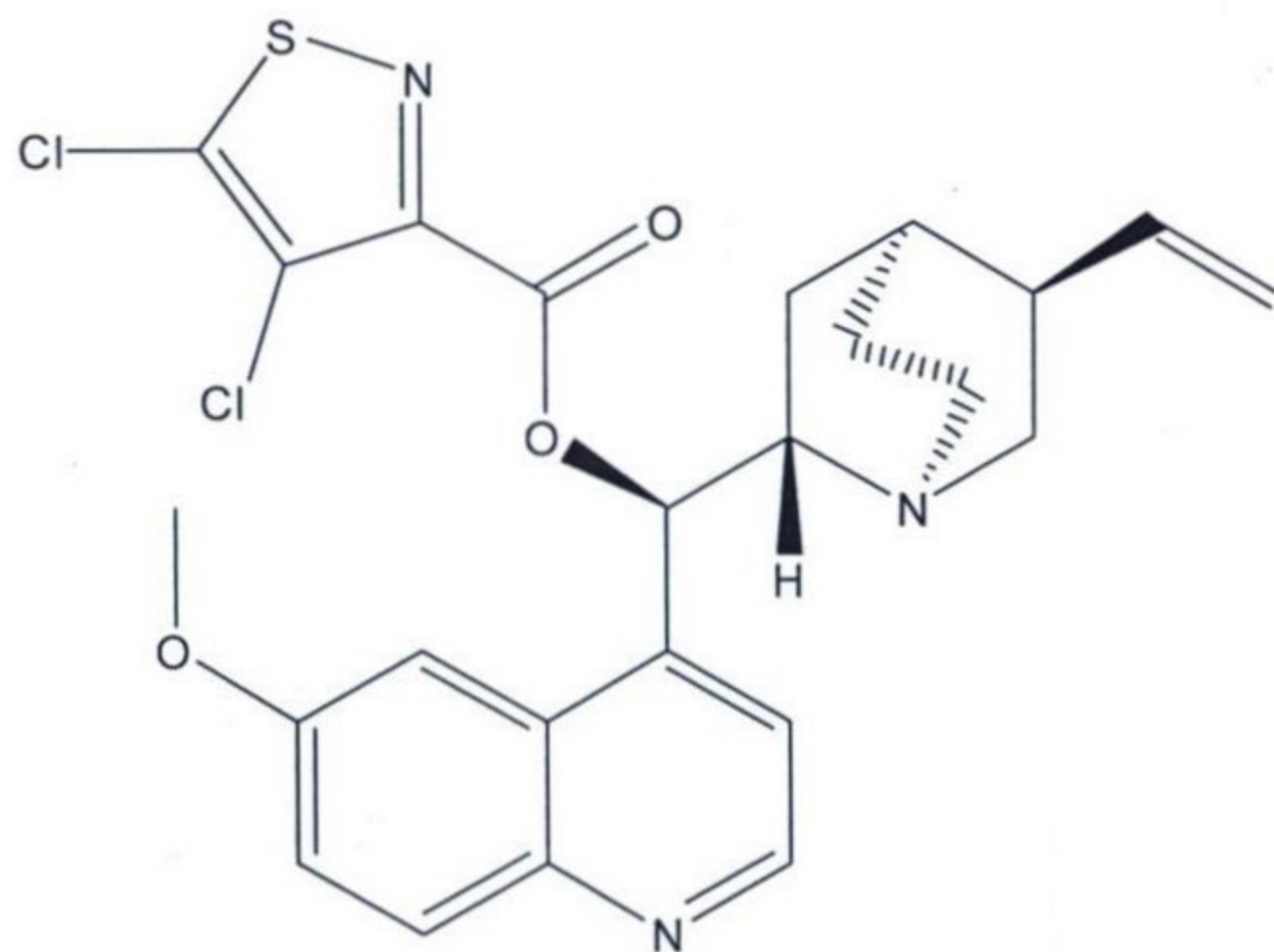

Molecular Weight: 504,43

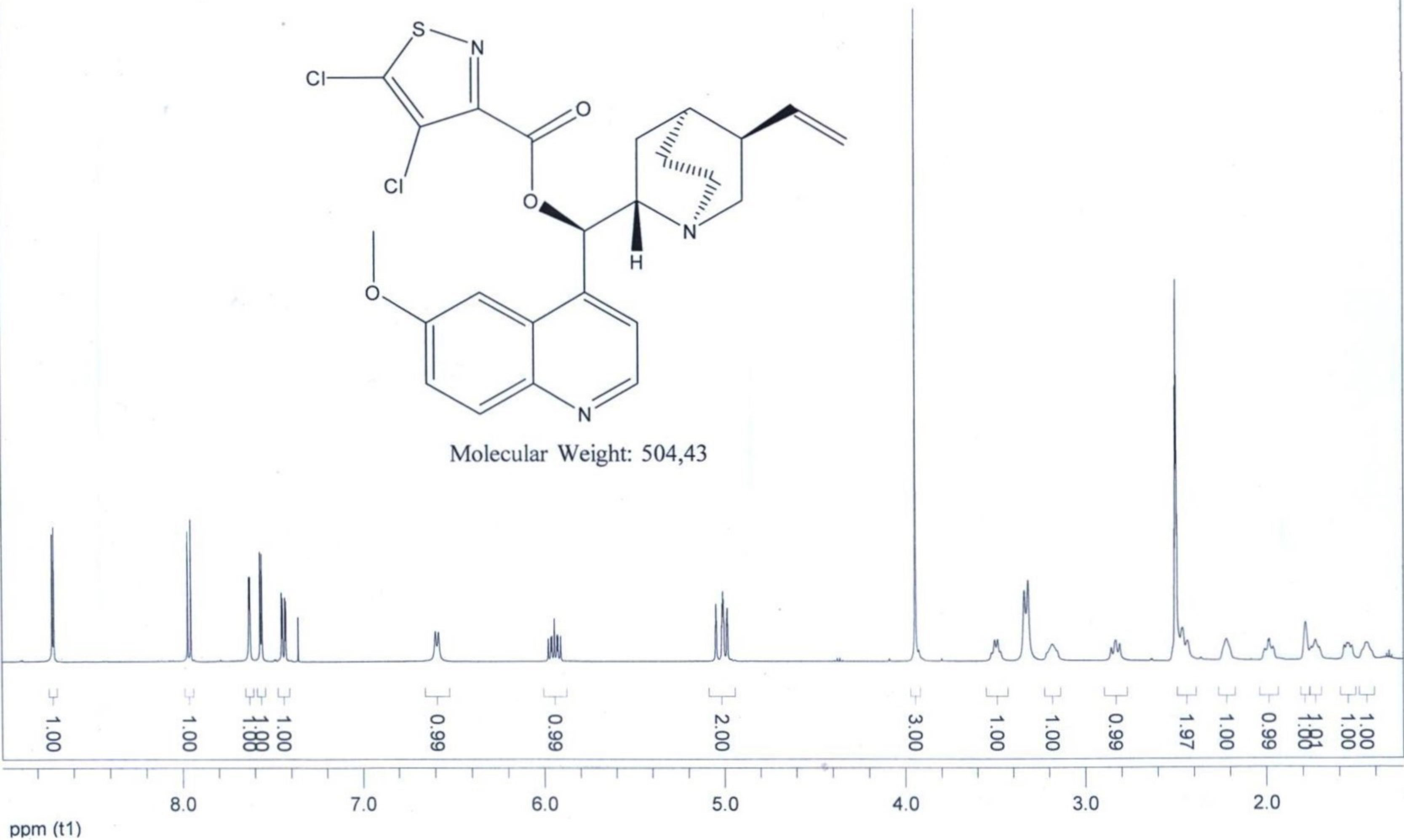

Supplement: Supplementary file 1 [file molecules-27-03476-s001.zip › NMR/2a ( H1, DMSO).pdf]

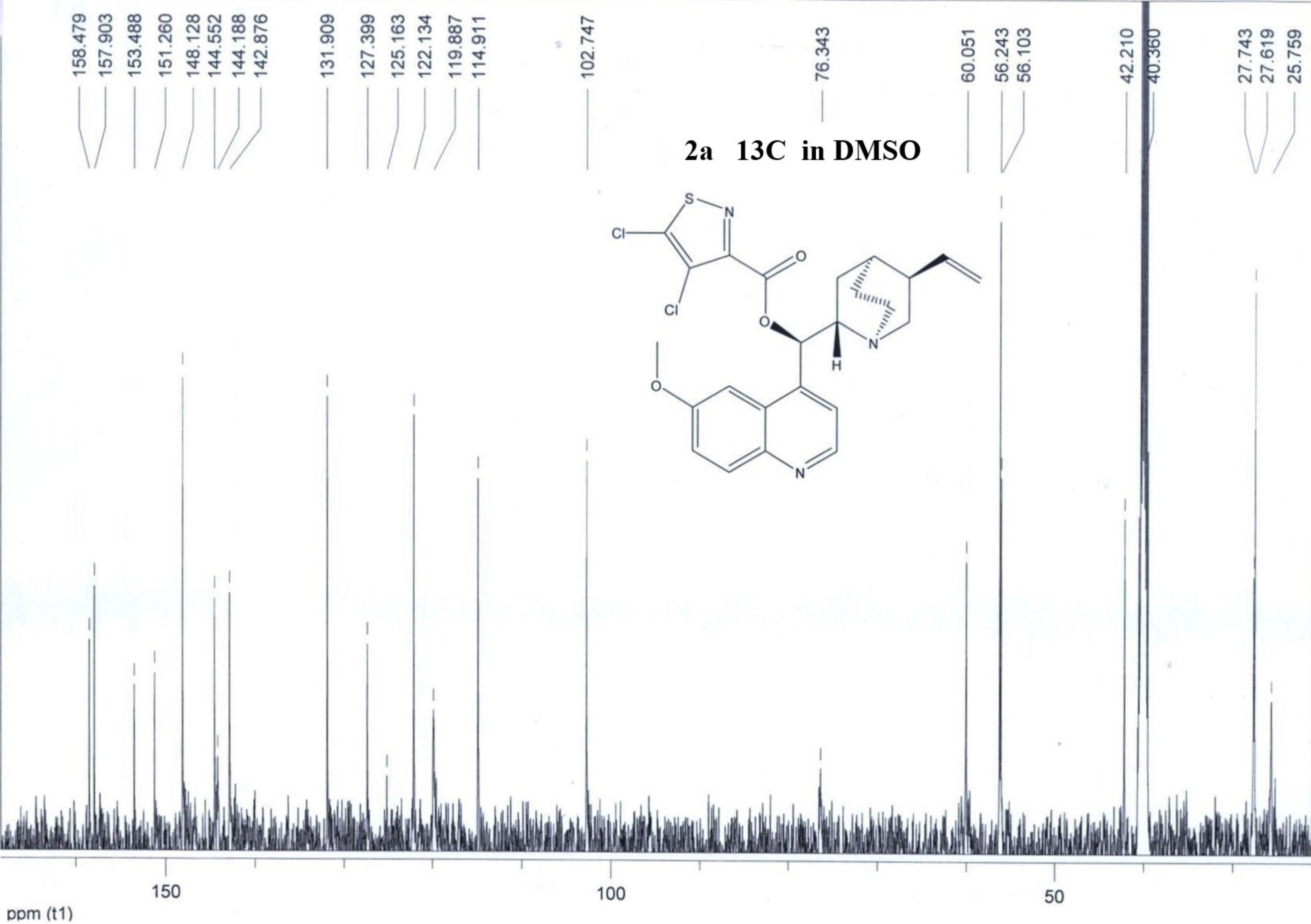

Supplement: Supplementary file 1 [file molecules-27-03476-s001.zip › NMR/2a (C13, DMSO).pdf]

# 2b <sup>13</sup>C DMSO-d<sub>6</sub>

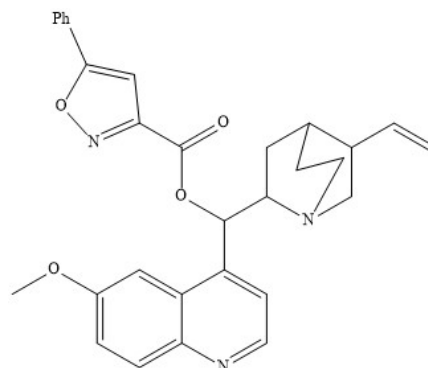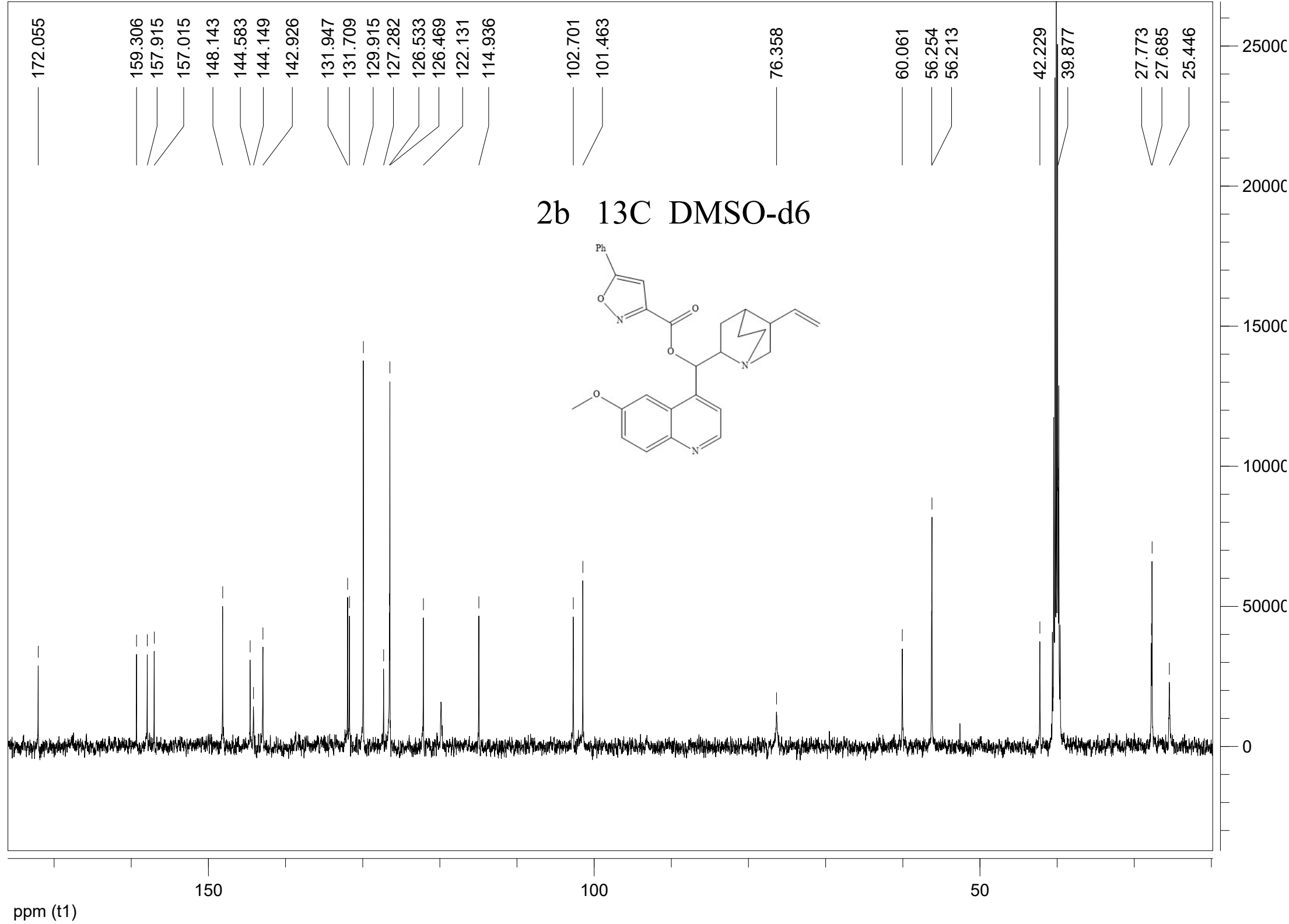

Supplement: Supplementary file 1 [file molecules-27-03476-s001.zip › NMR/2b (13C).pdf]

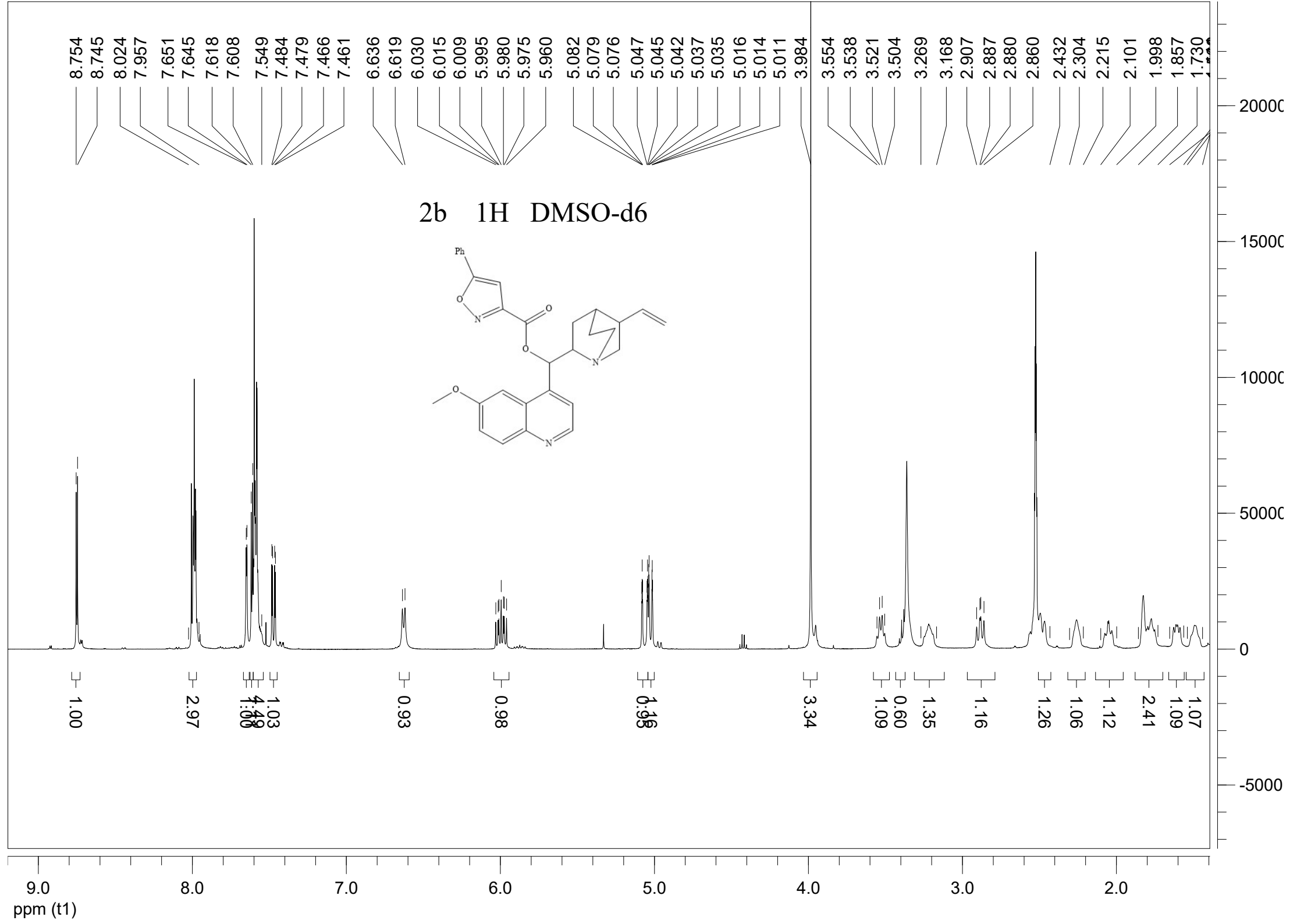

Supplement: Supplementary file 1 [file molecules-27-03476-s001.zip › NMR/2b (1H).pdf]

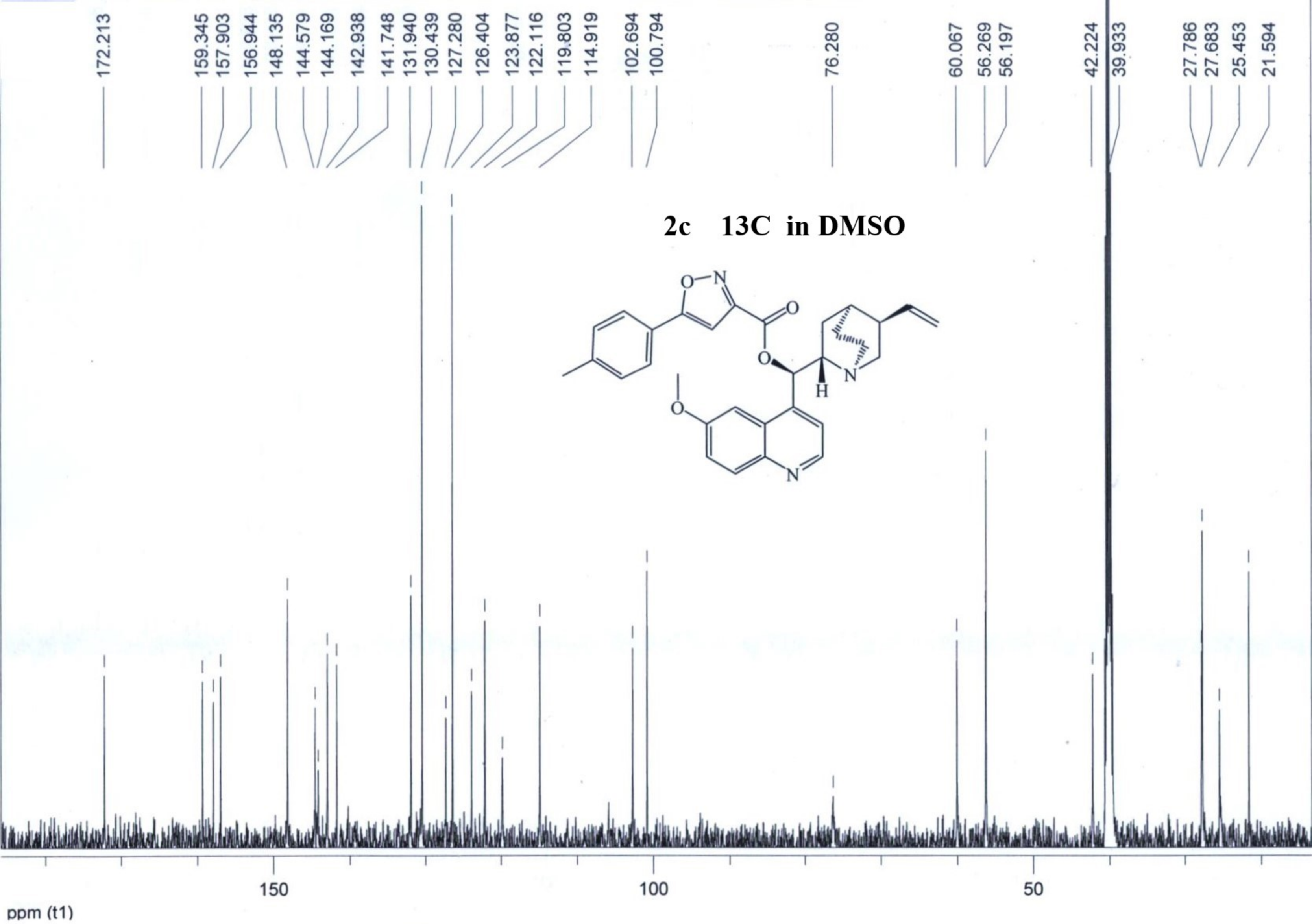

Supplement: Supplementary file 1 [file molecules-27-03476-s001.zip › NMR/2c (13C, DMSO).pdf]

**2c 1H**  
**in DMSO-d6**

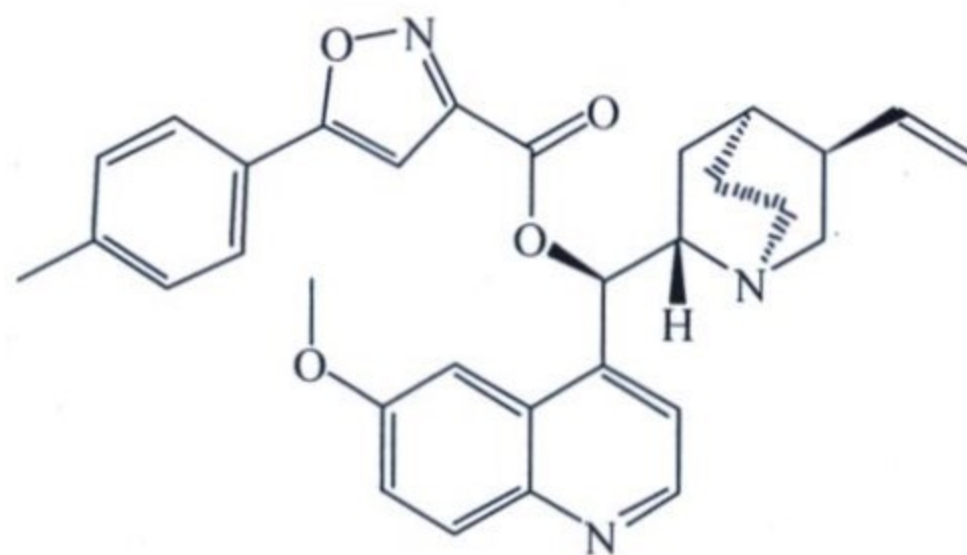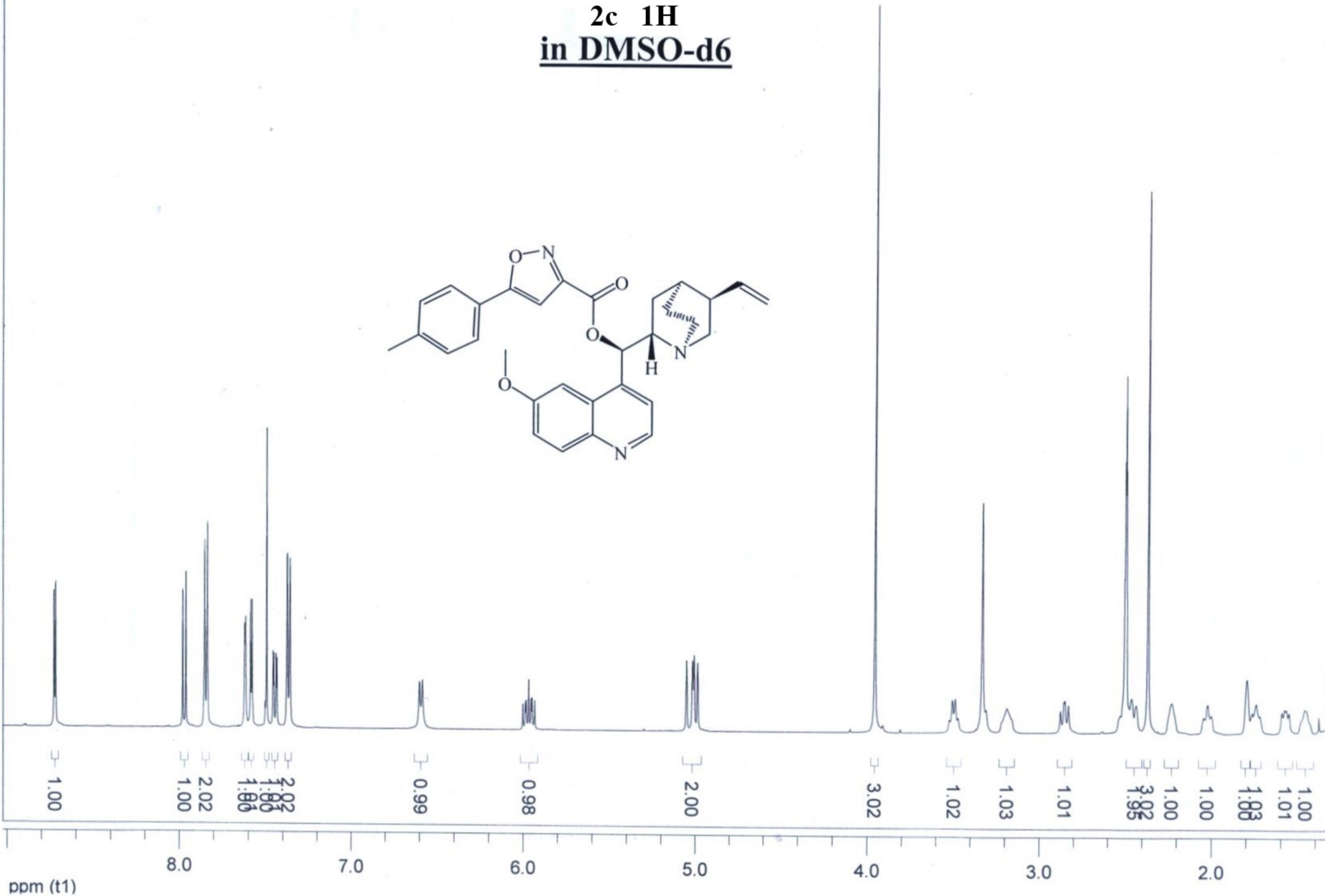

Supplement: Supplementary file 1 [file molecules-27-03476-s001.zip › NMR/2c (1H, DMSO).pdf]

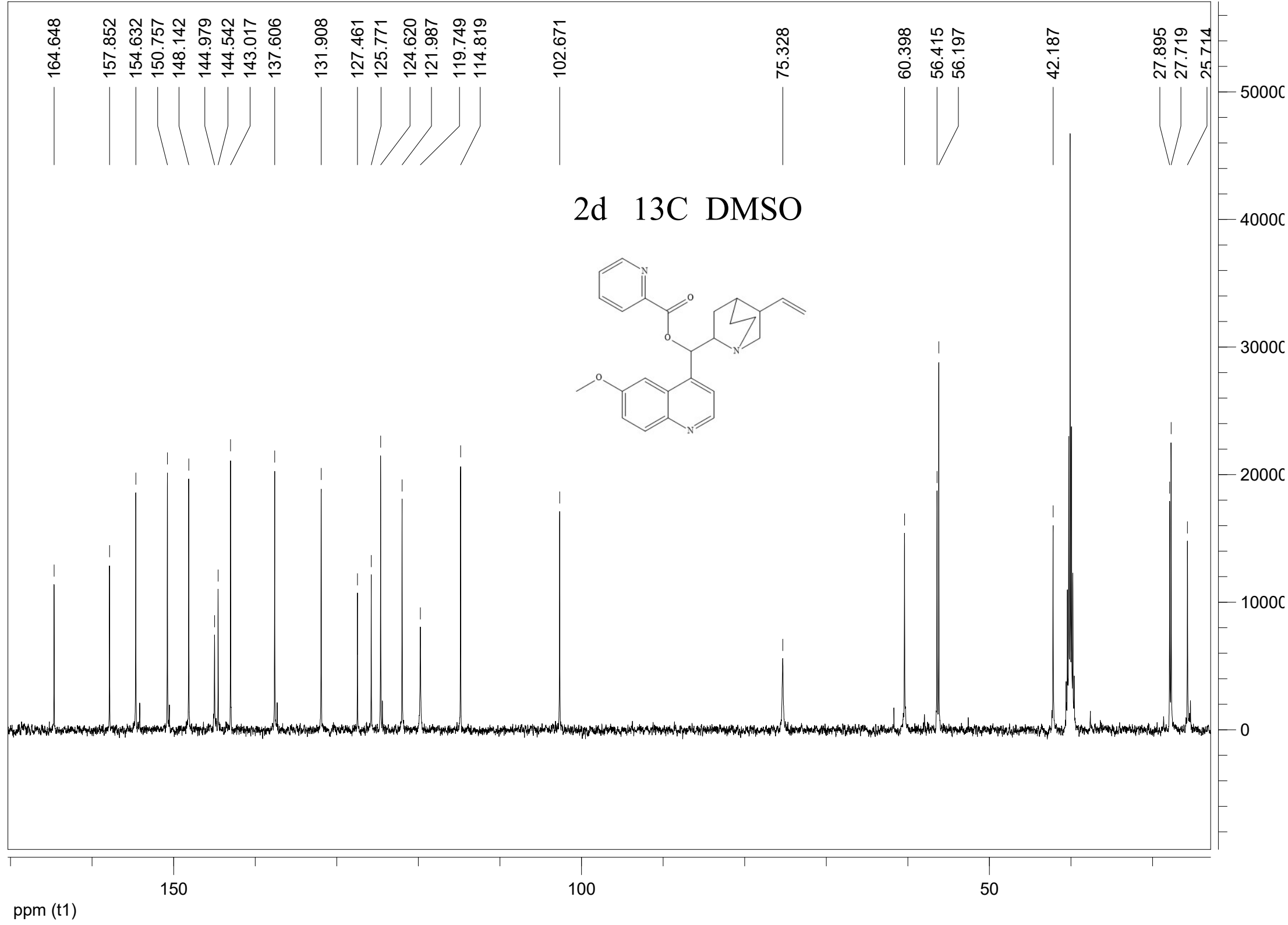

Supplement: Supplementary file 1 [file molecules-27-03476-s001.zip › NMR/2d (13C).pdf]

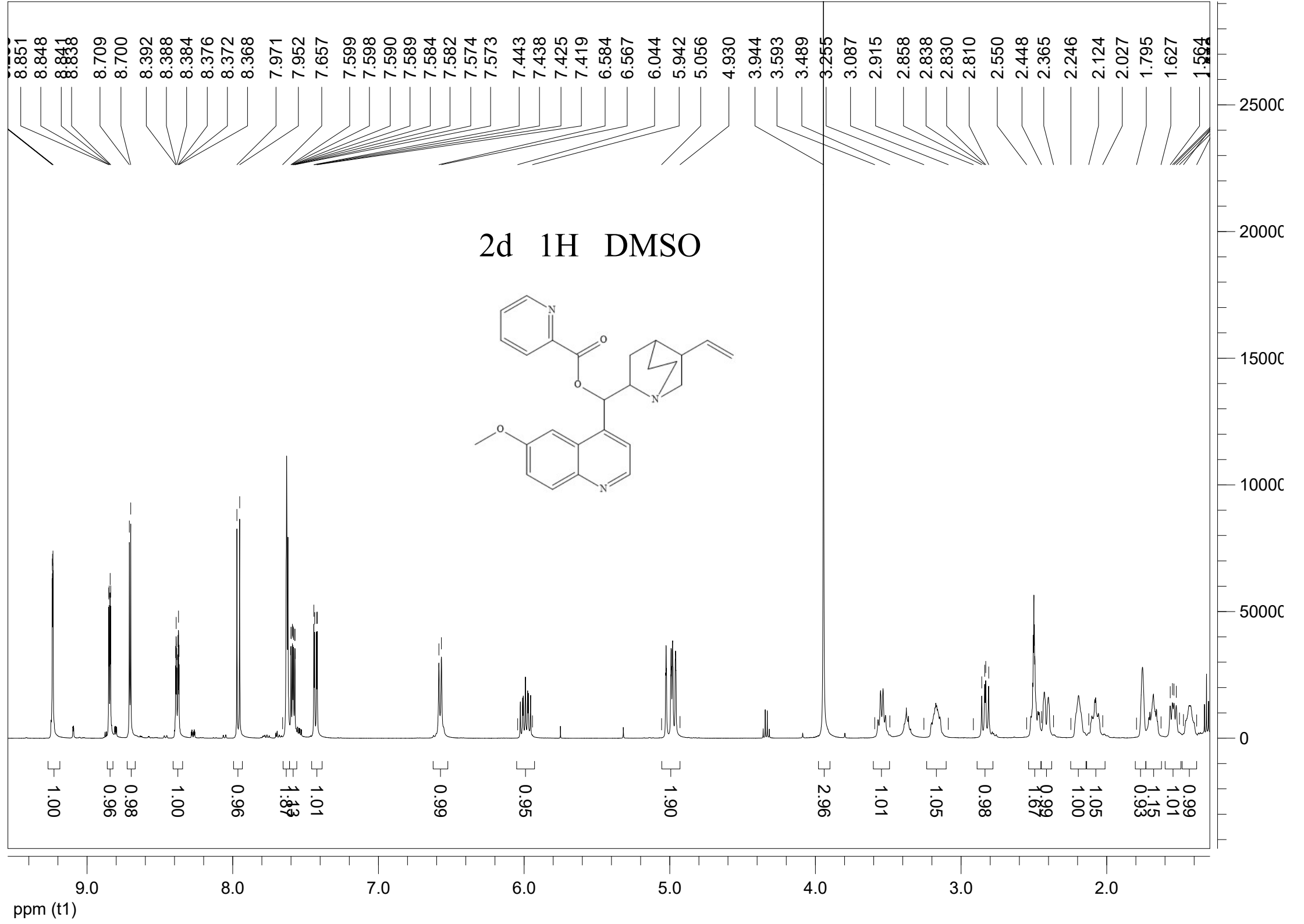

Supplement: Supplementary file 1 [file molecules-27-03476-s001.zip › NMR/2d (1H).pdf]

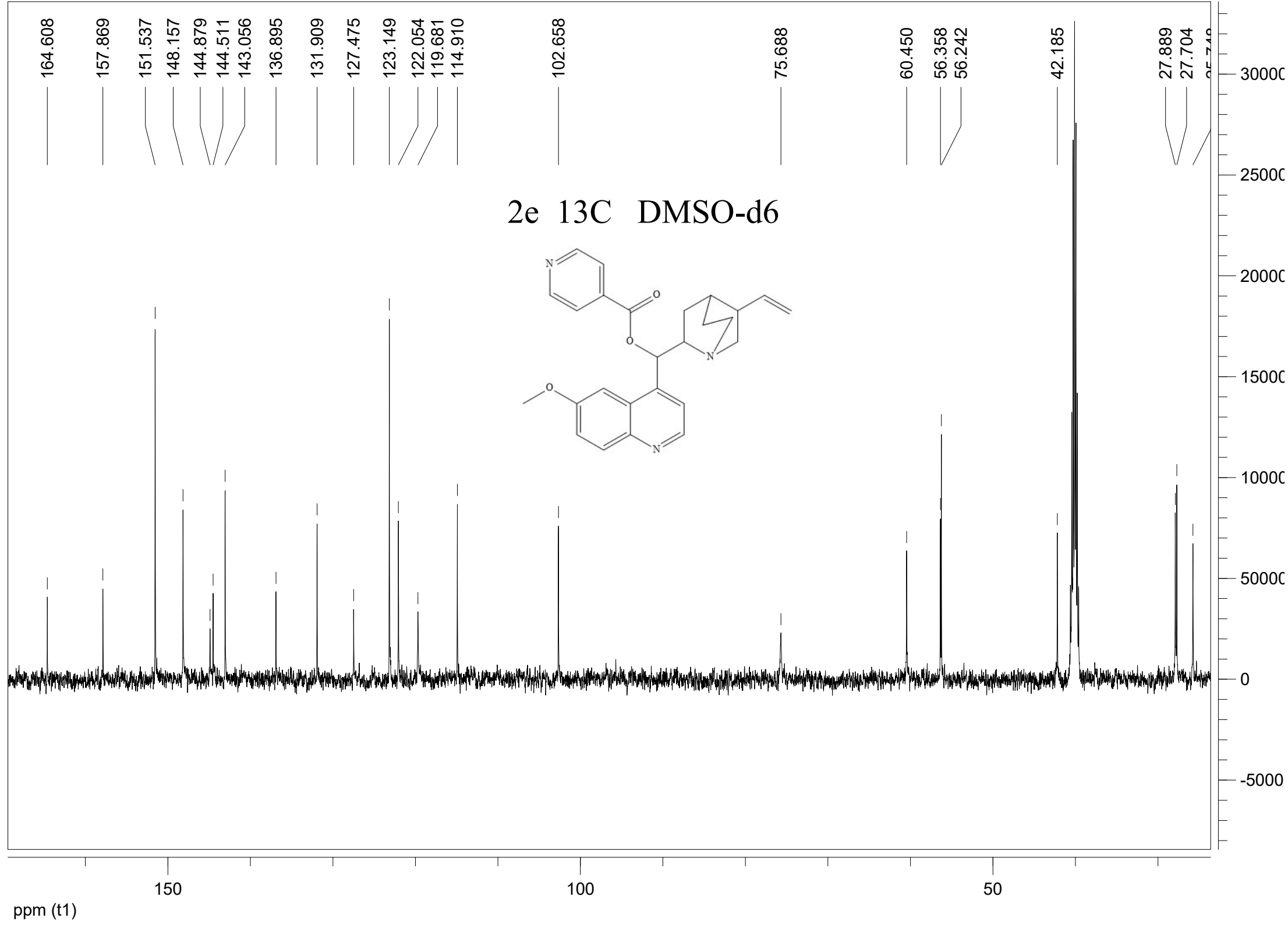

Supplement: Supplementary file 1 [file molecules-27-03476-s001.zip › NMR/2e (13H).pdf]

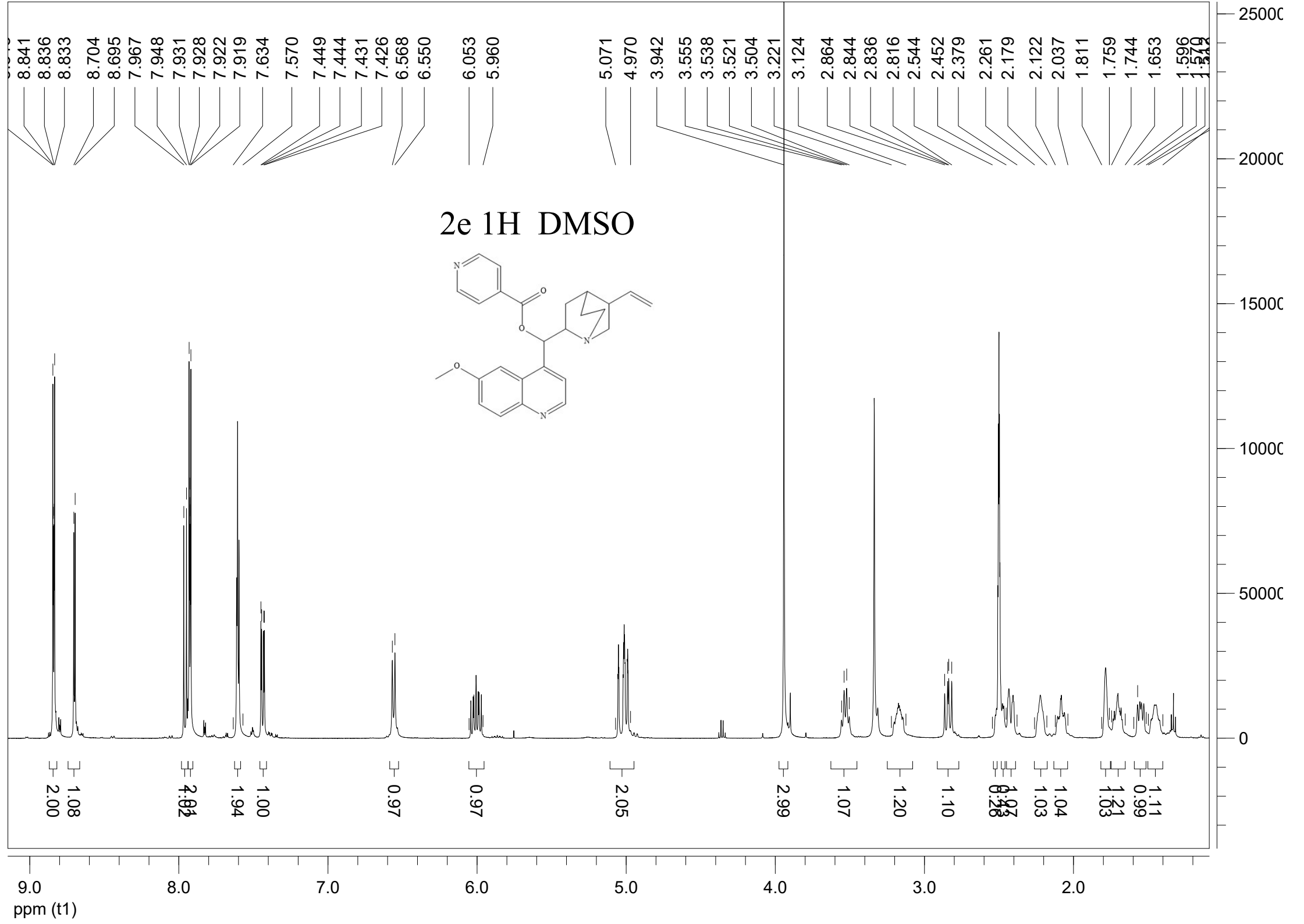

Supplement: Supplementary file 1 [file molecules-27-03476-s001.zip › NMR/2e (1H).pdf]

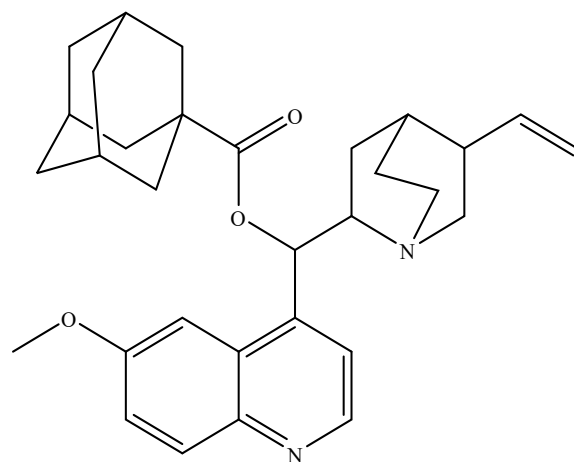

2f  $^{13}\text{C}$  in  $\text{CDCl}_3$

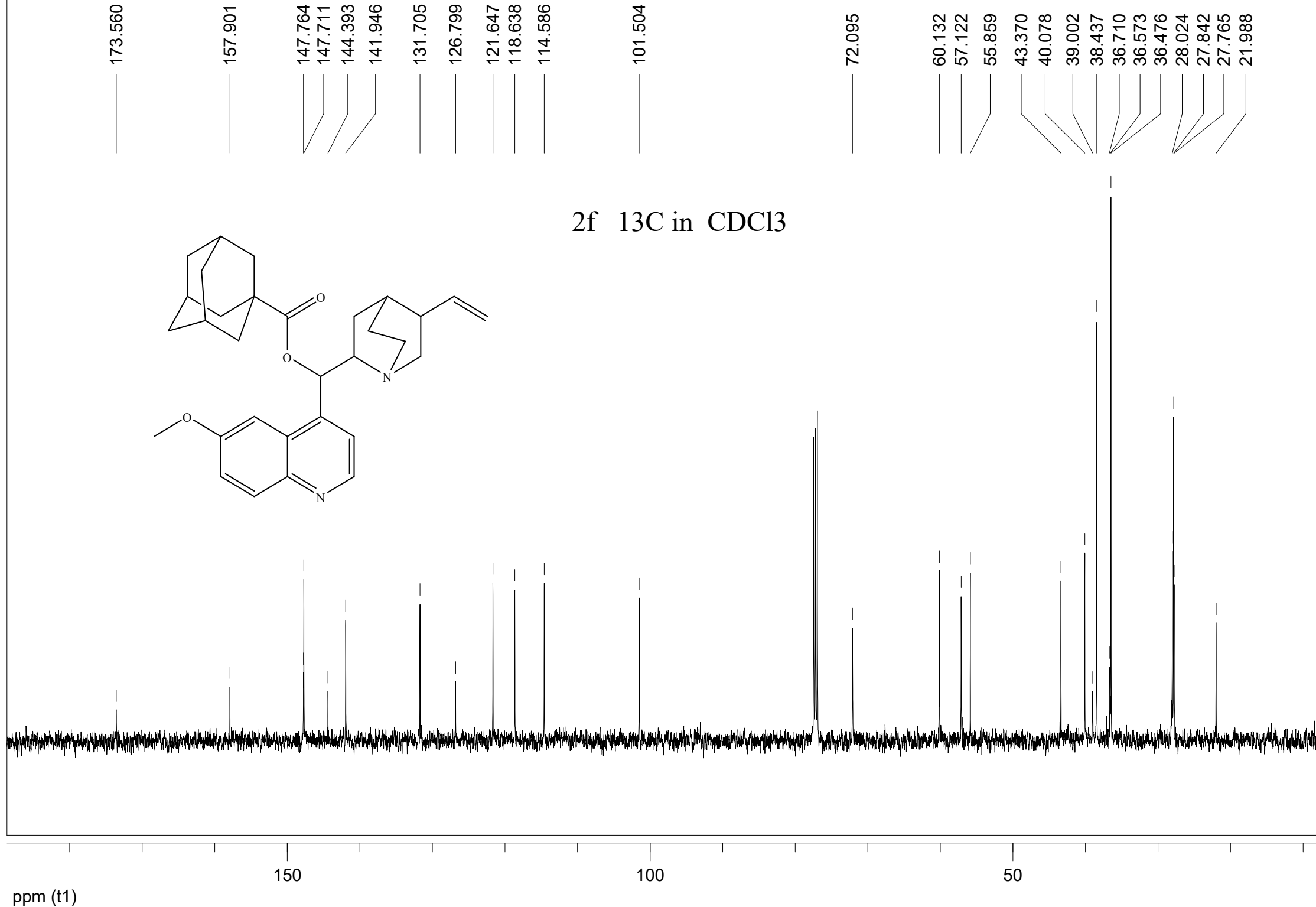

Supplement: Supplementary file 1 [file molecules-27-03476-s001.zip › NMR/2f (13C,CDCl3).pdf]

2f <sup>1</sup>H in CDCl<sub>3</sub>

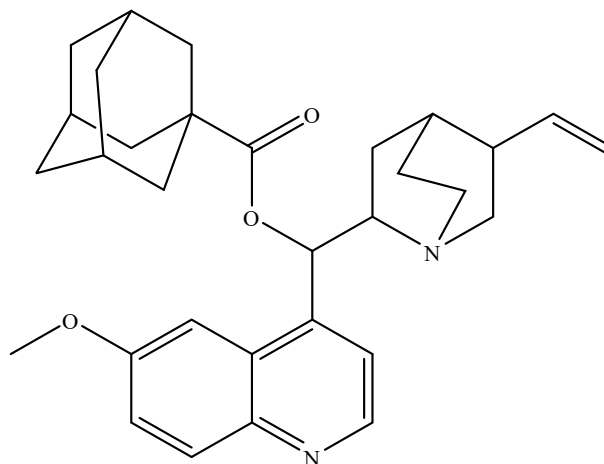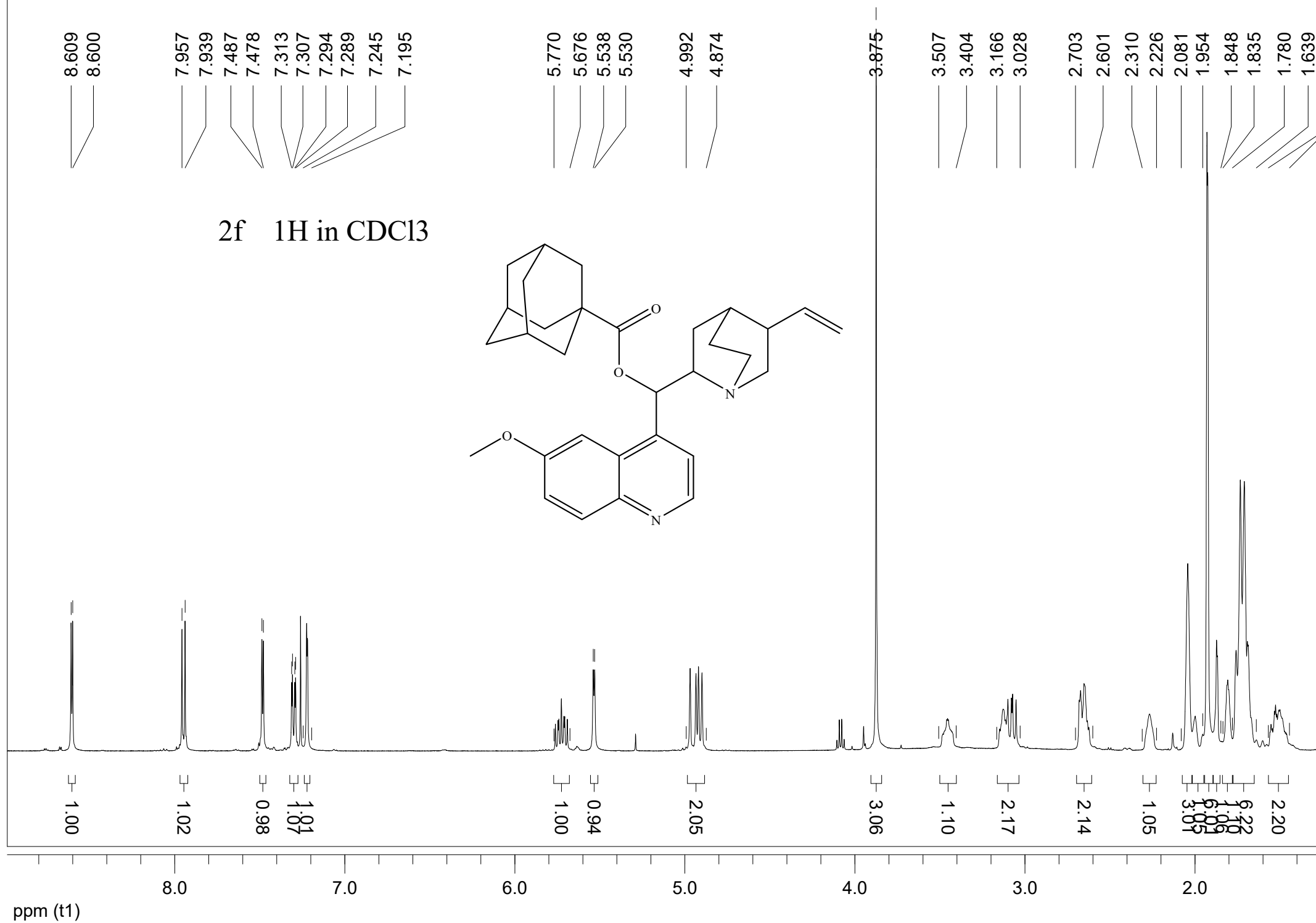

Supplement: Supplementary file 1 [file molecules-27-03476-s001.zip › NMR/2f (1H,CDCl3).pdf]

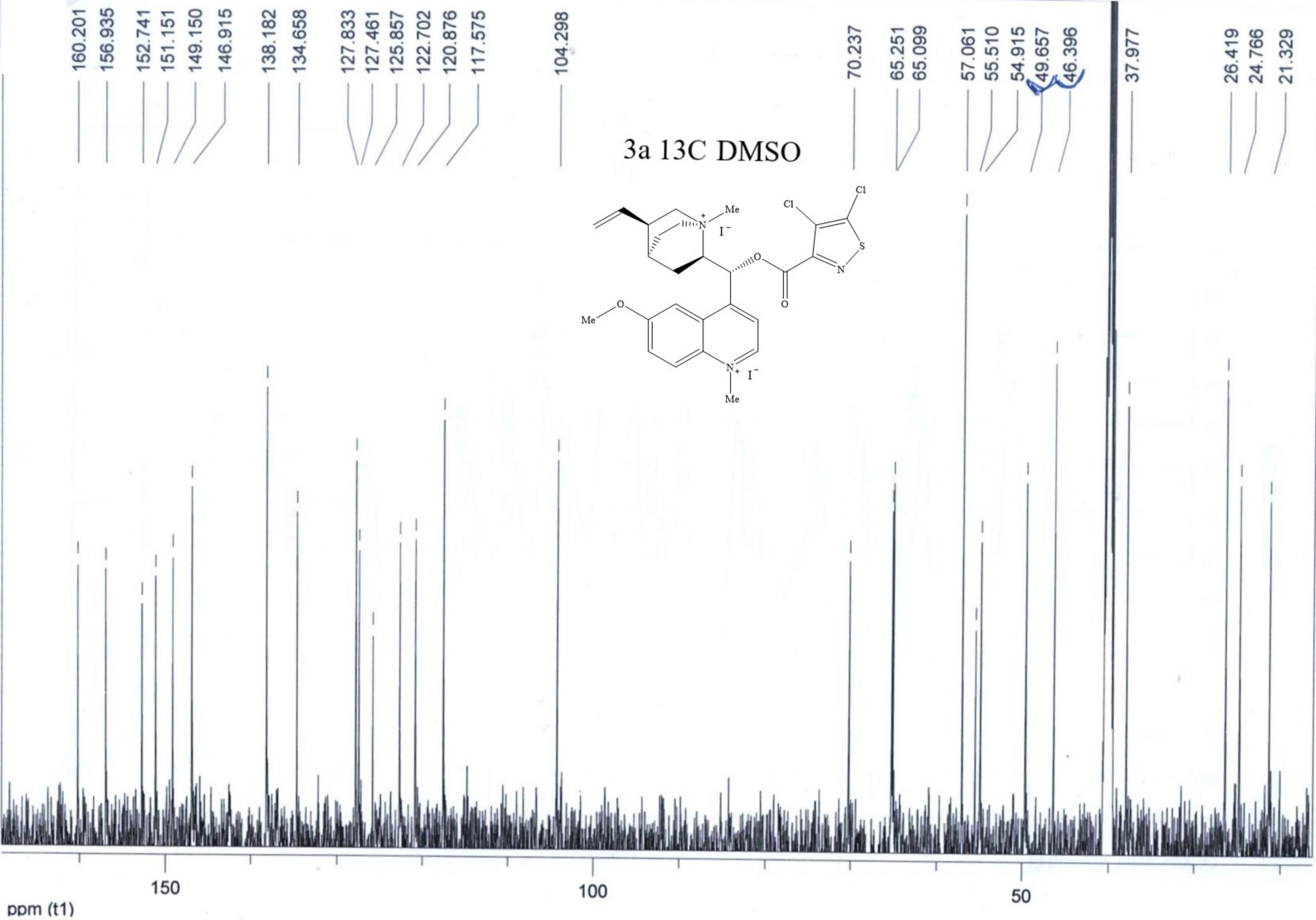

Supplement: Supplementary file 1 [file molecules-27-03476-s001.zip › NMR/3a (13C).pdf]

# 3a 1H DMSO

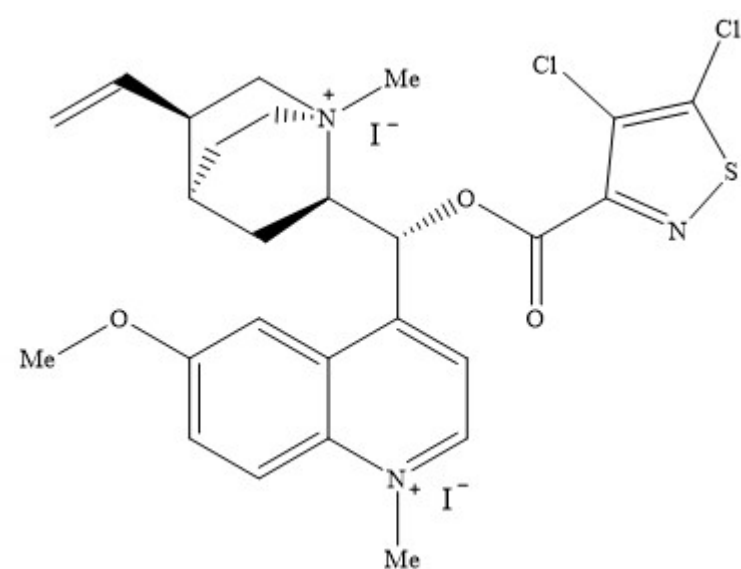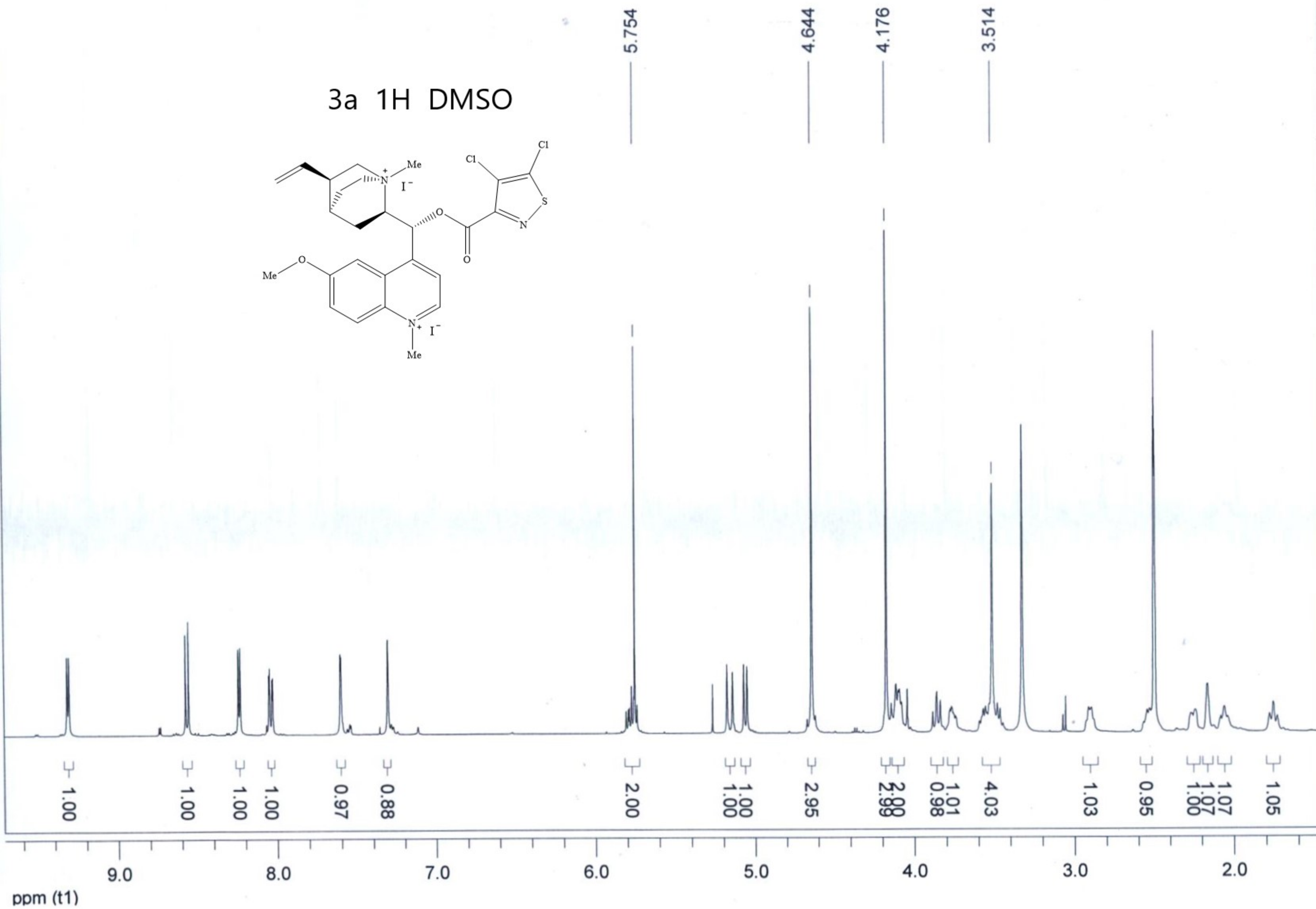

Supplement: Supplementary file 1 [file molecules-27-03476-s001.zip › NMR/3a (H1).pdf]

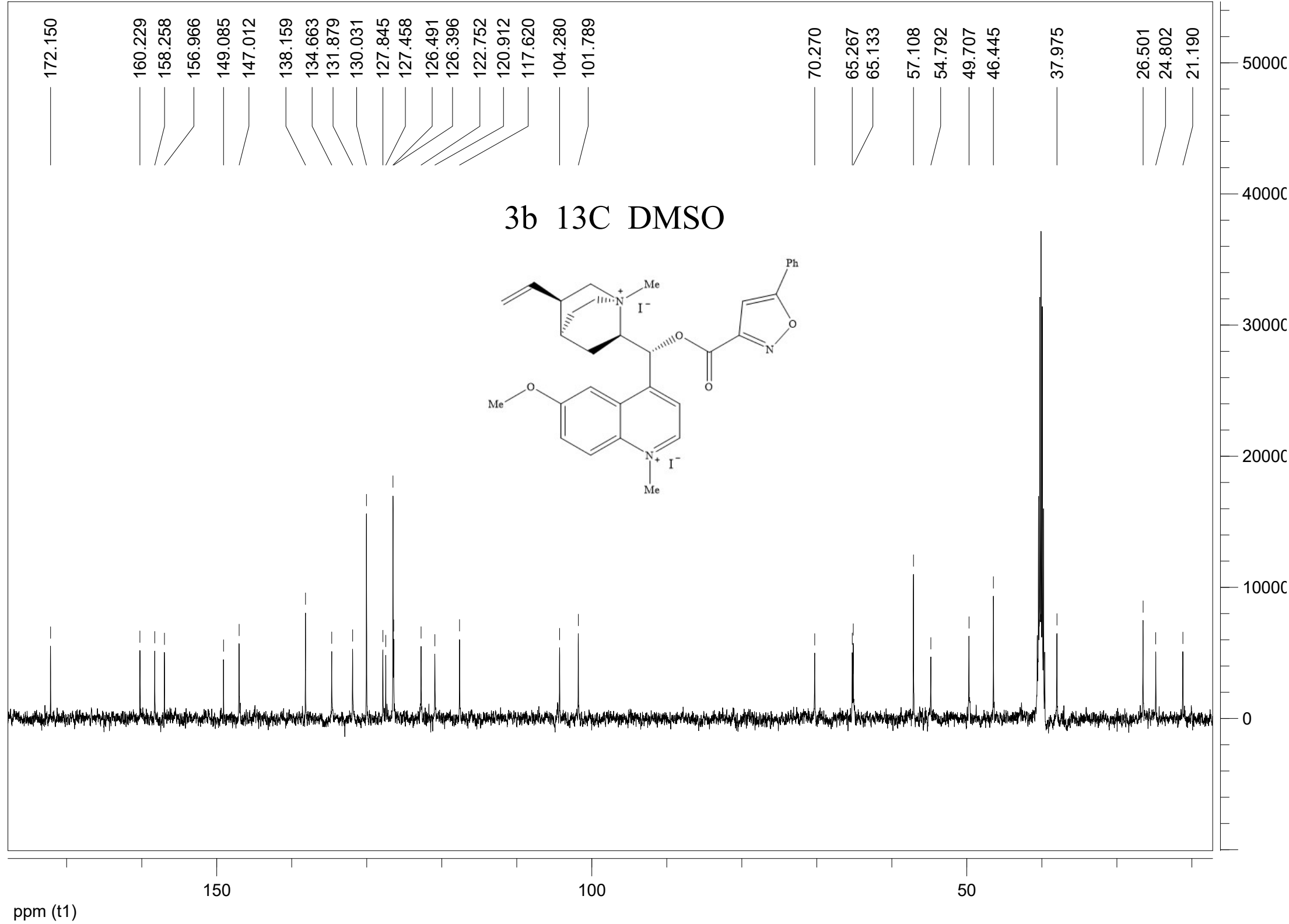

Supplement: Supplementary file 1 [file molecules-27-03476-s001.zip › NMR/3b (13C).pdf]

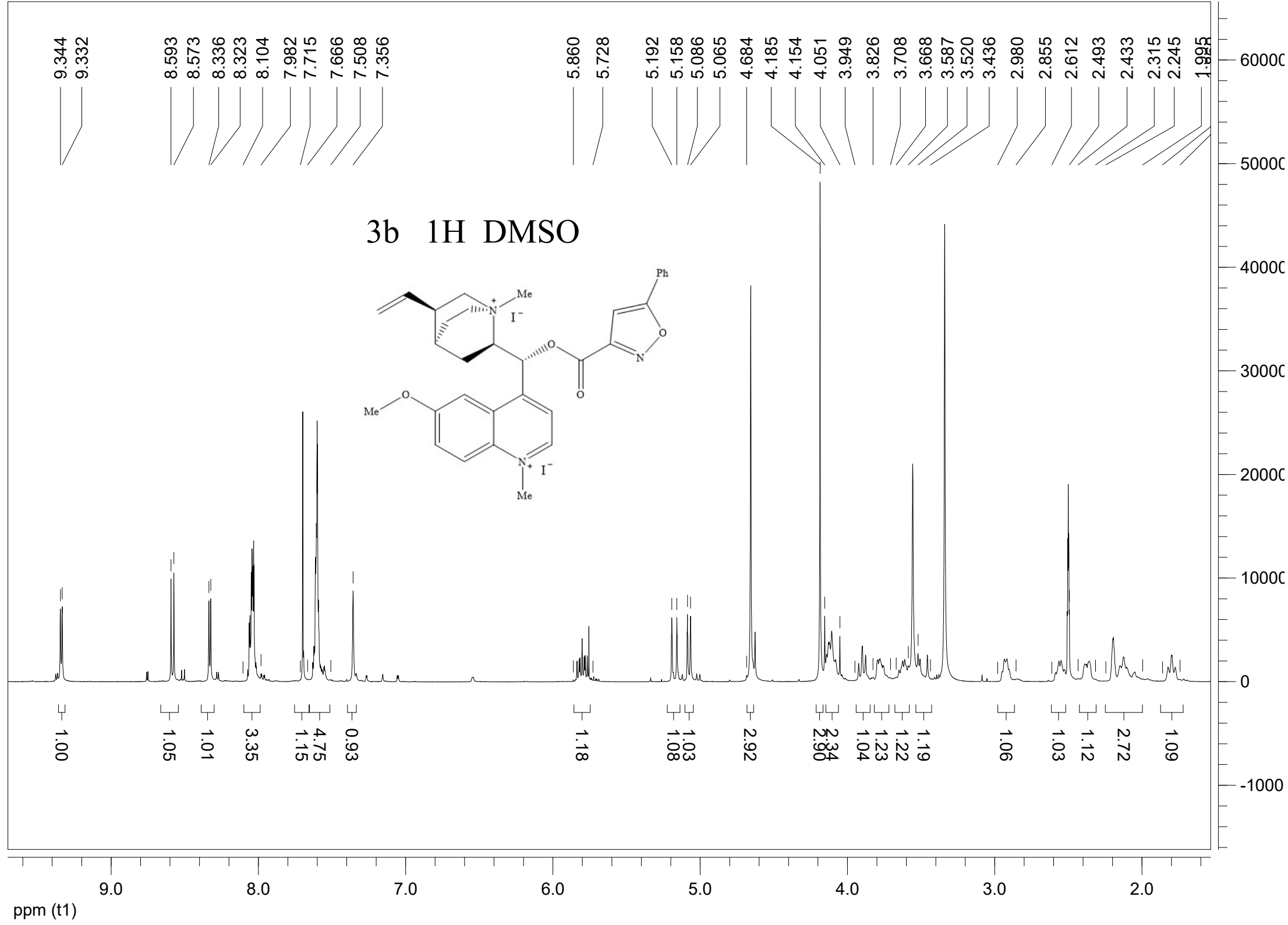

Supplement: Supplementary file 1 [file molecules-27-03476-s001.zip › NMR/3b (1H).pdf]

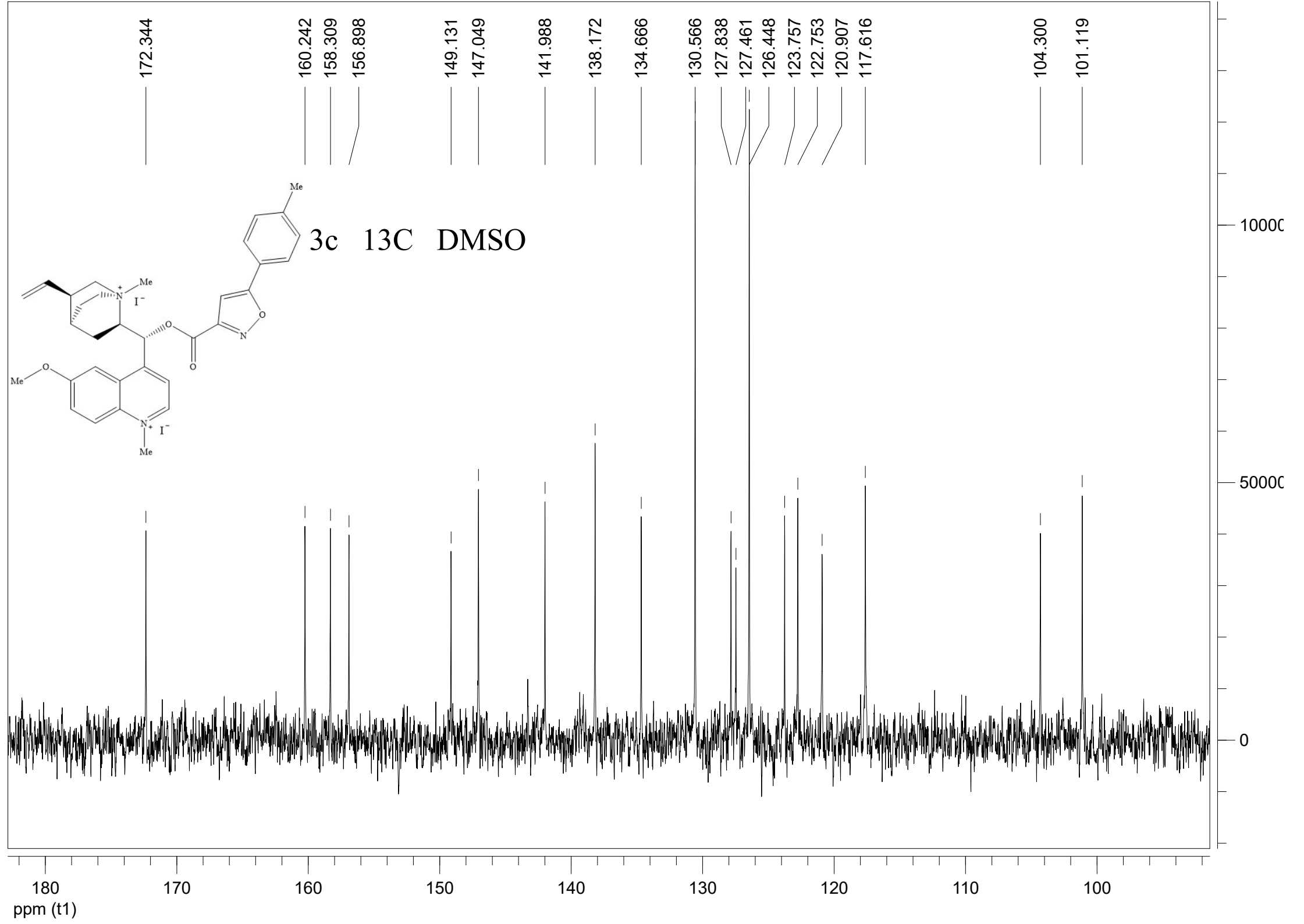

Supplement: Supplementary file 1 [file molecules-27-03476-s001.zip › NMR/3c (13C, DMSO).pdf]

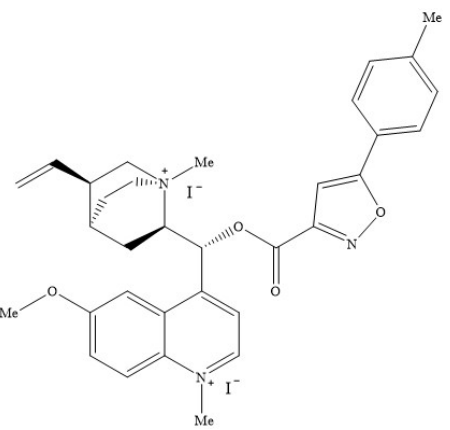

3c 1H DMSO

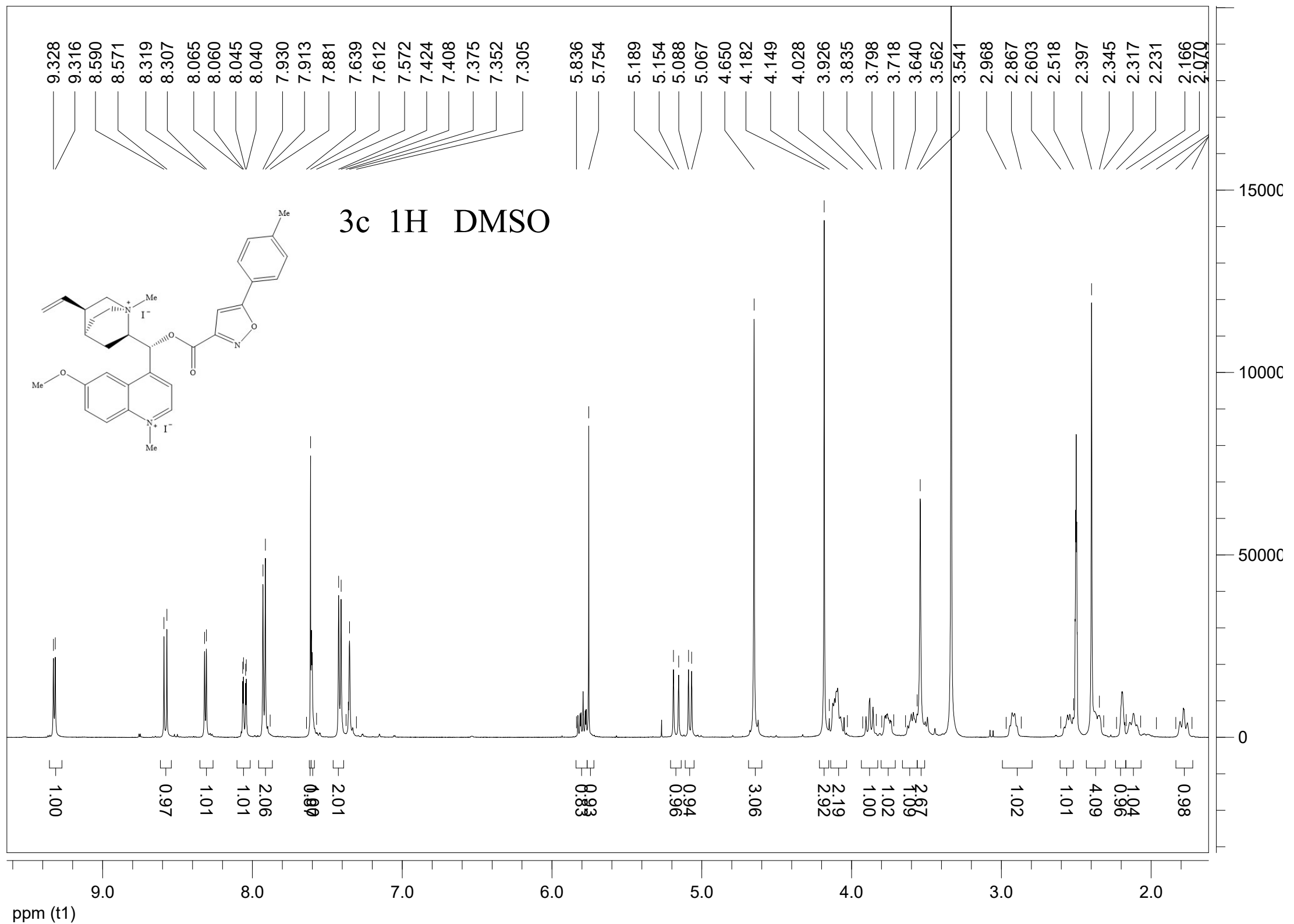

Supplement: Supplementary file 1 [file molecules-27-03476-s001.zip › NMR/3c (1H, DMSO).pdf]

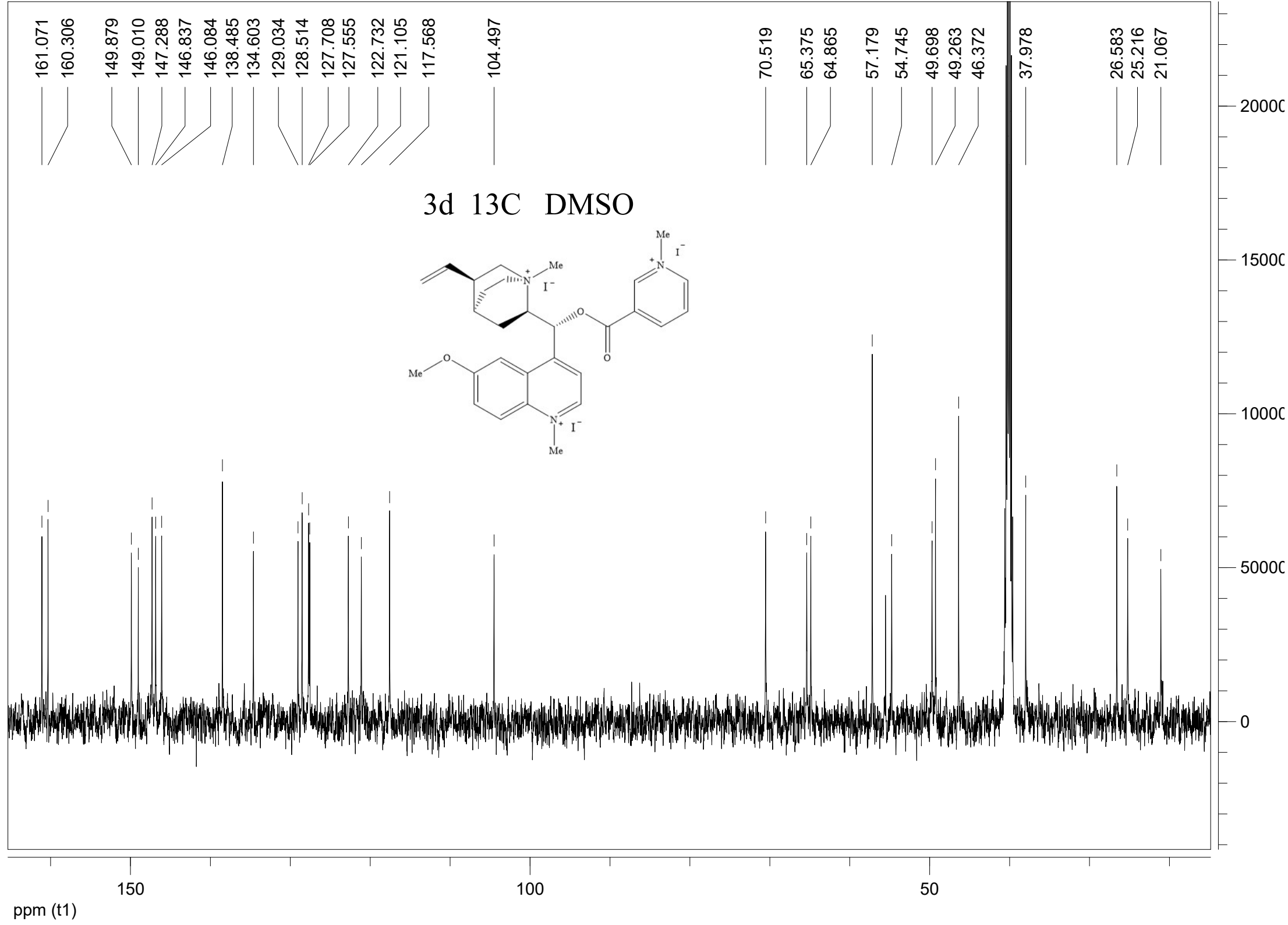

Supplement: Supplementary file 1 [file molecules-27-03476-s001.zip › NMR/3d (13C).pdf]

# 3d <sup>1</sup>H DMSO

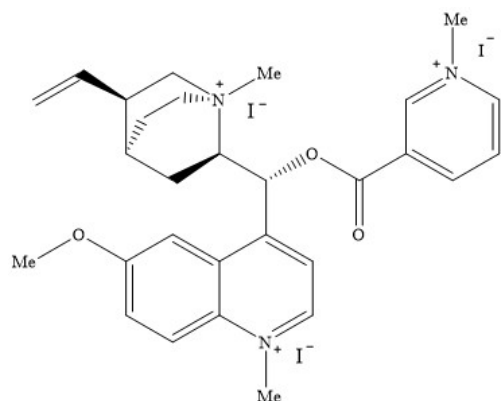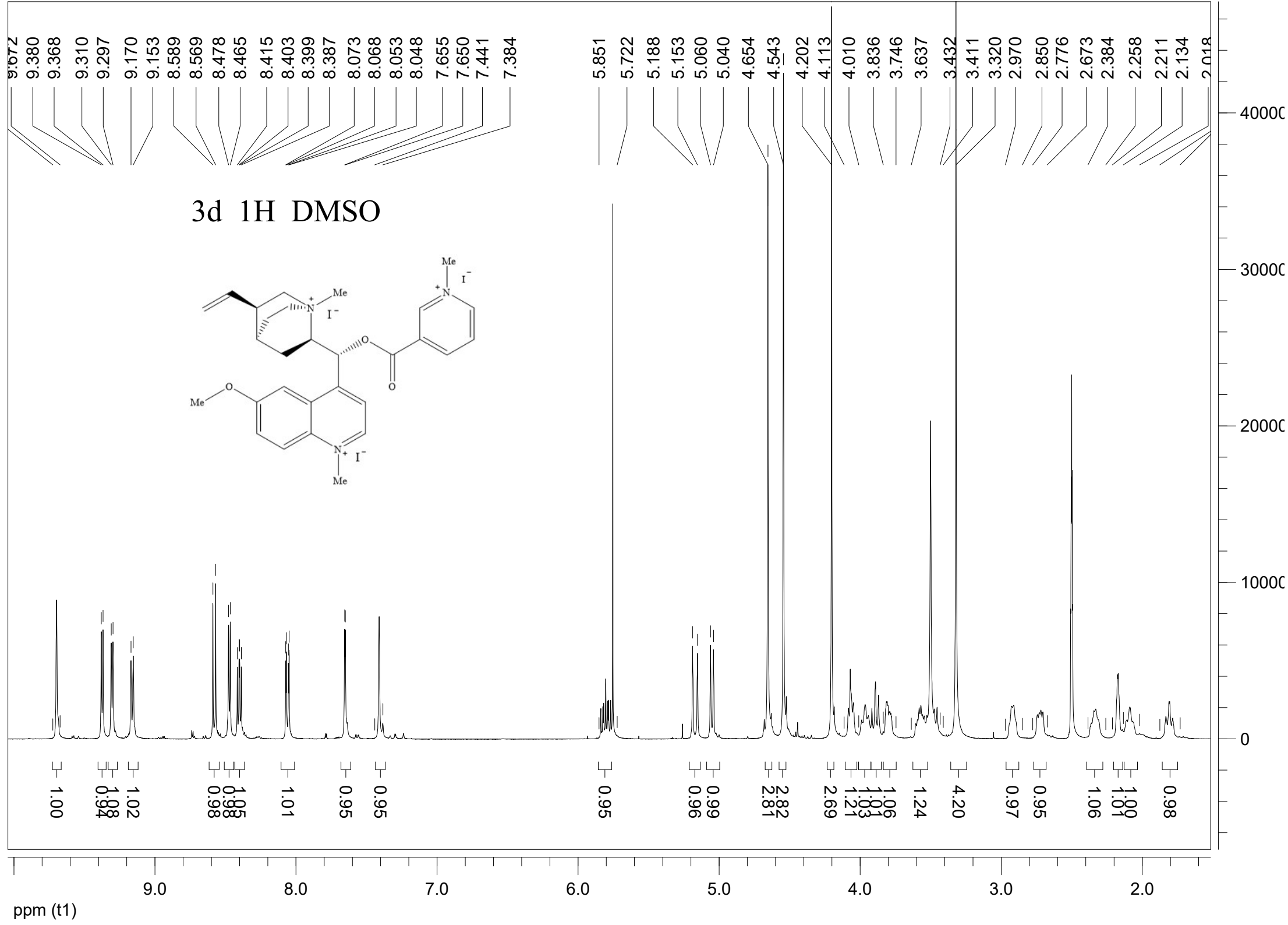

Supplement: Supplementary file 1 [file molecules-27-03476-s001.zip › NMR/3d (1H).pdf]

# 3e <sup>13</sup>C DMSO

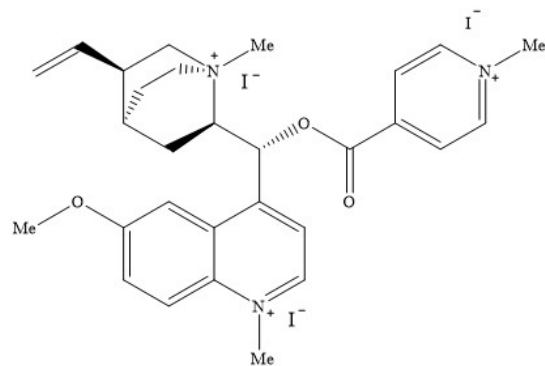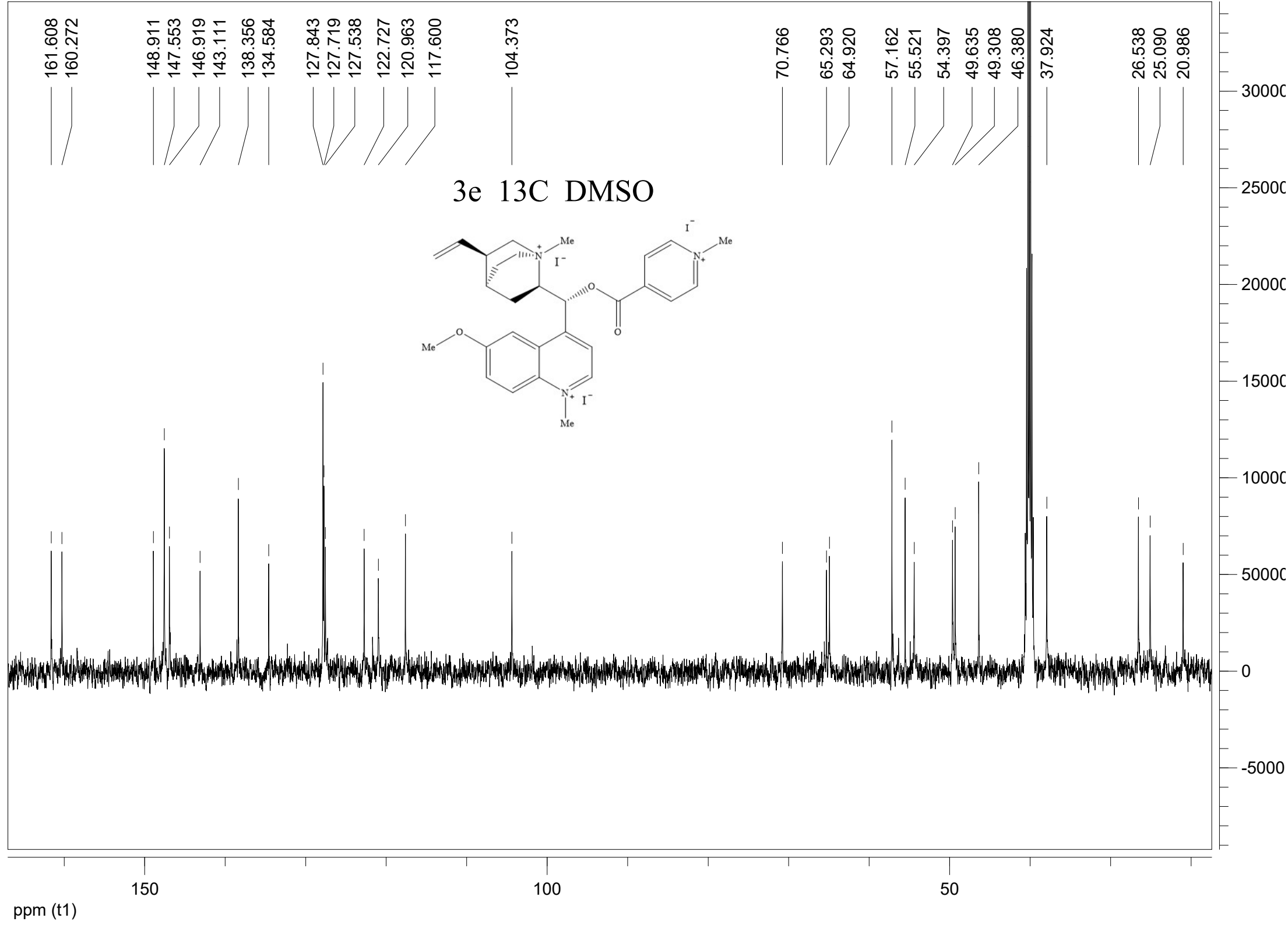

Supplement: Supplementary file 1 [file molecules-27-03476-s001.zip › NMR/3e (13C).pdf]

3e <sup>1</sup>H DMSO-d<sub>6</sub>

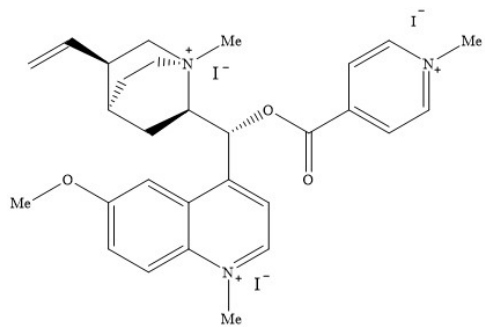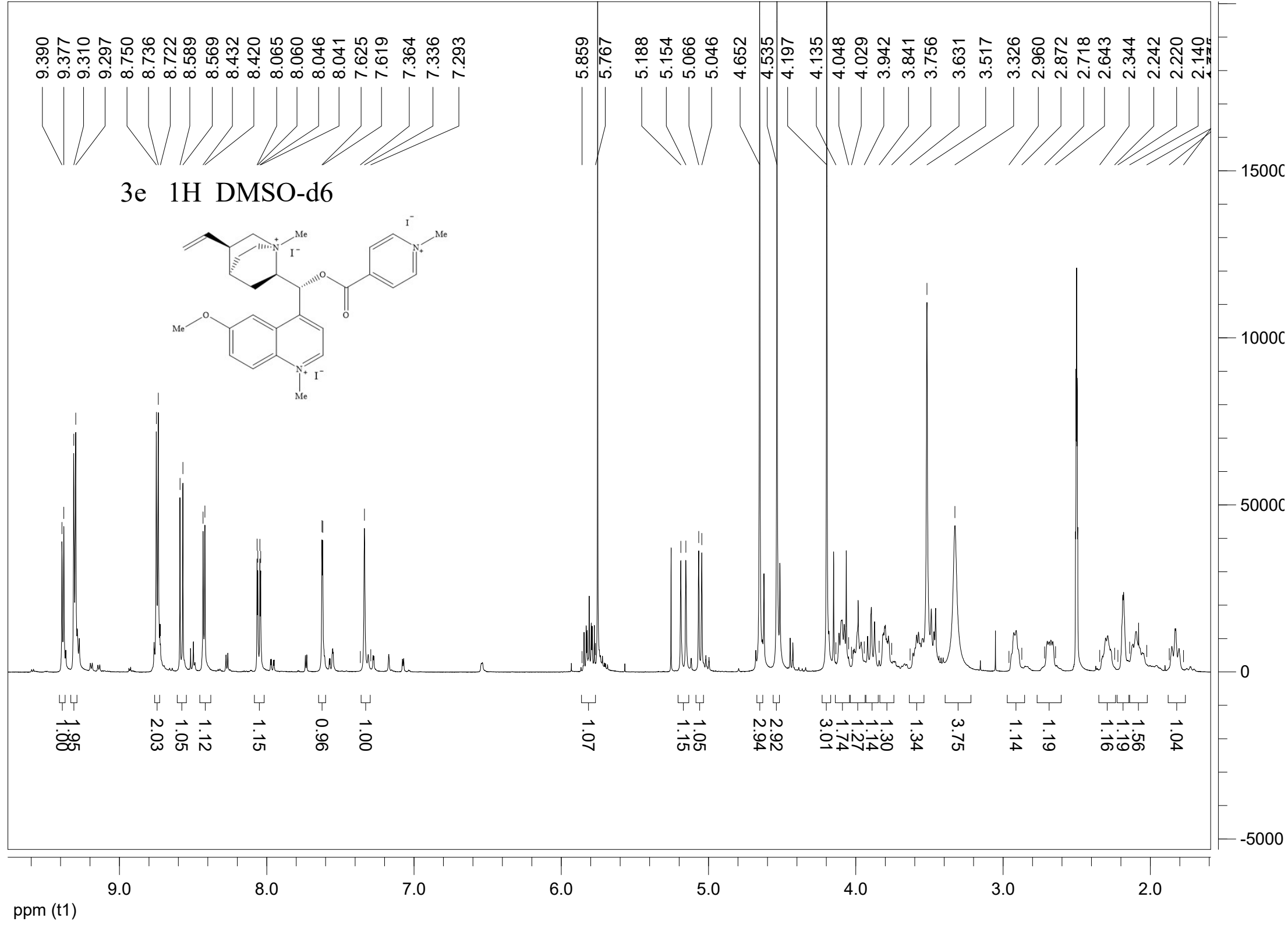

Supplement: Supplementary file 1 [file molecules-27-03476-s001.zip › NMR/3e (1H).pdf]

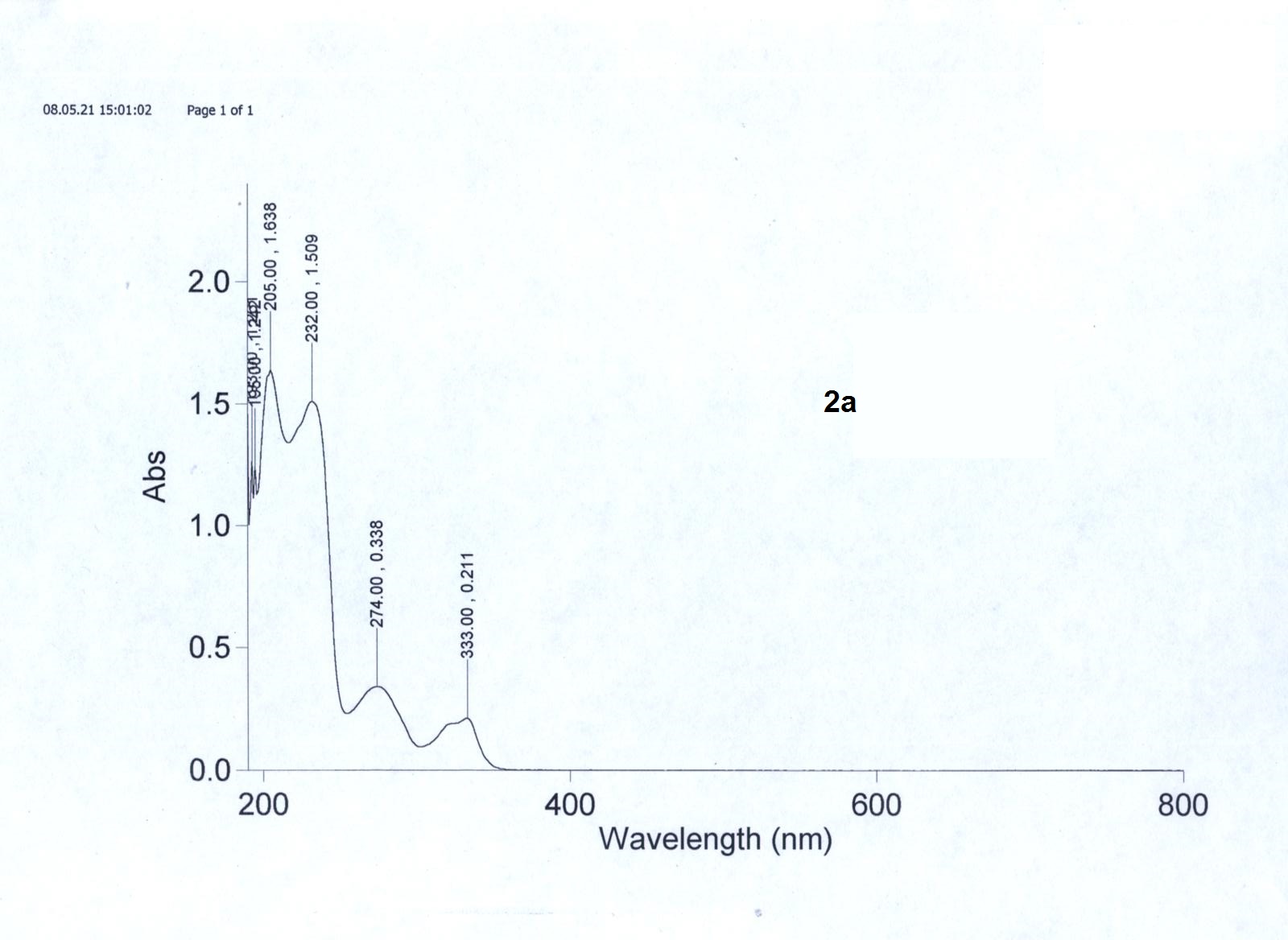

Supplement: Supplementary file 1 [file molecules-27-03476-s001.zip › UV/2a UV.jpg]

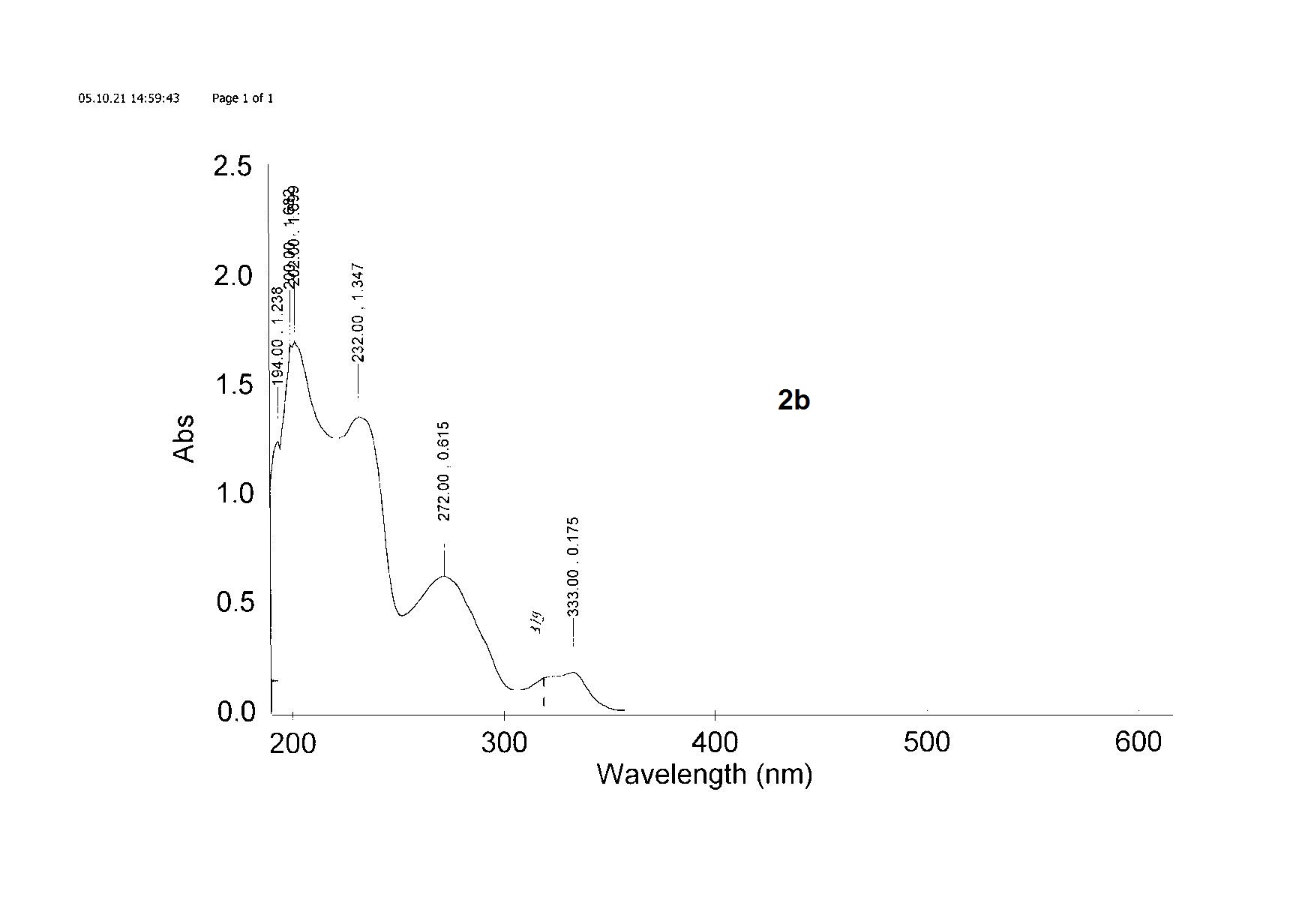

Supplement: Supplementary file 1 [file molecules-27-03476-s001.zip › UV/2b UV.jpg]

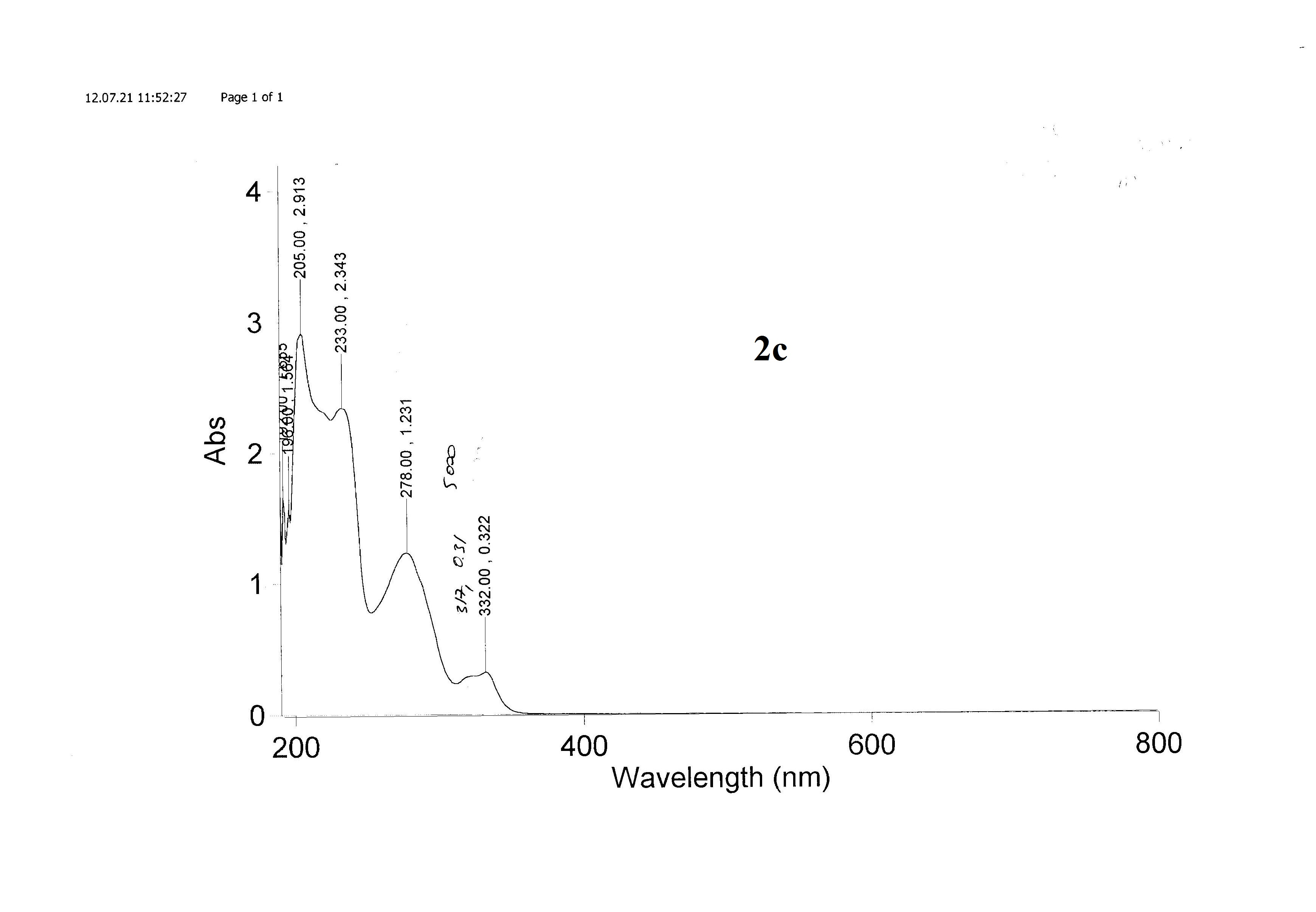

Supplement: Supplementary file 1 [file molecules-27-03476-s001.zip › UV/2c uv.jpg]

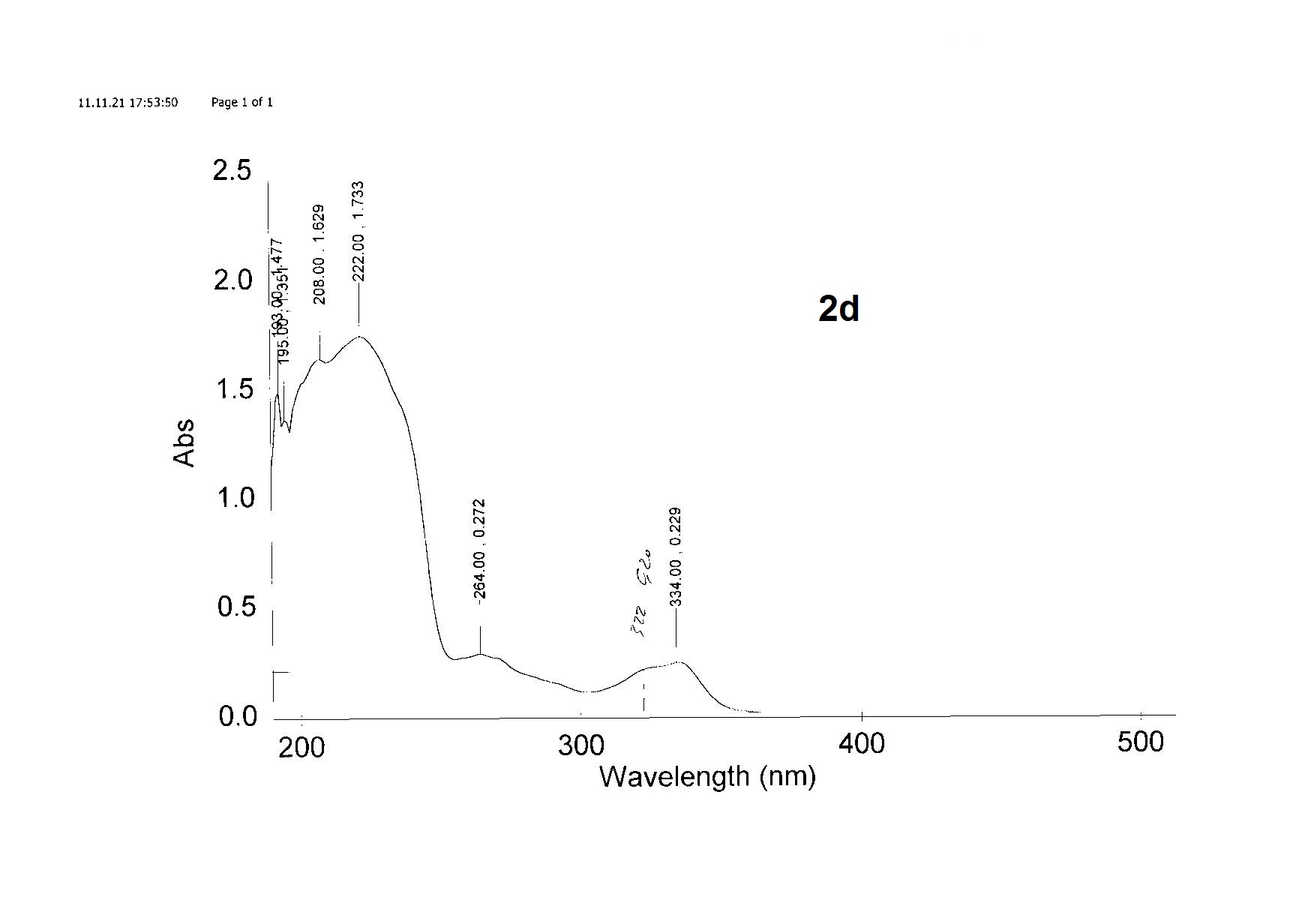

Supplement: Supplementary file 1 [file molecules-27-03476-s001.zip › UV/2d UV.jpg]

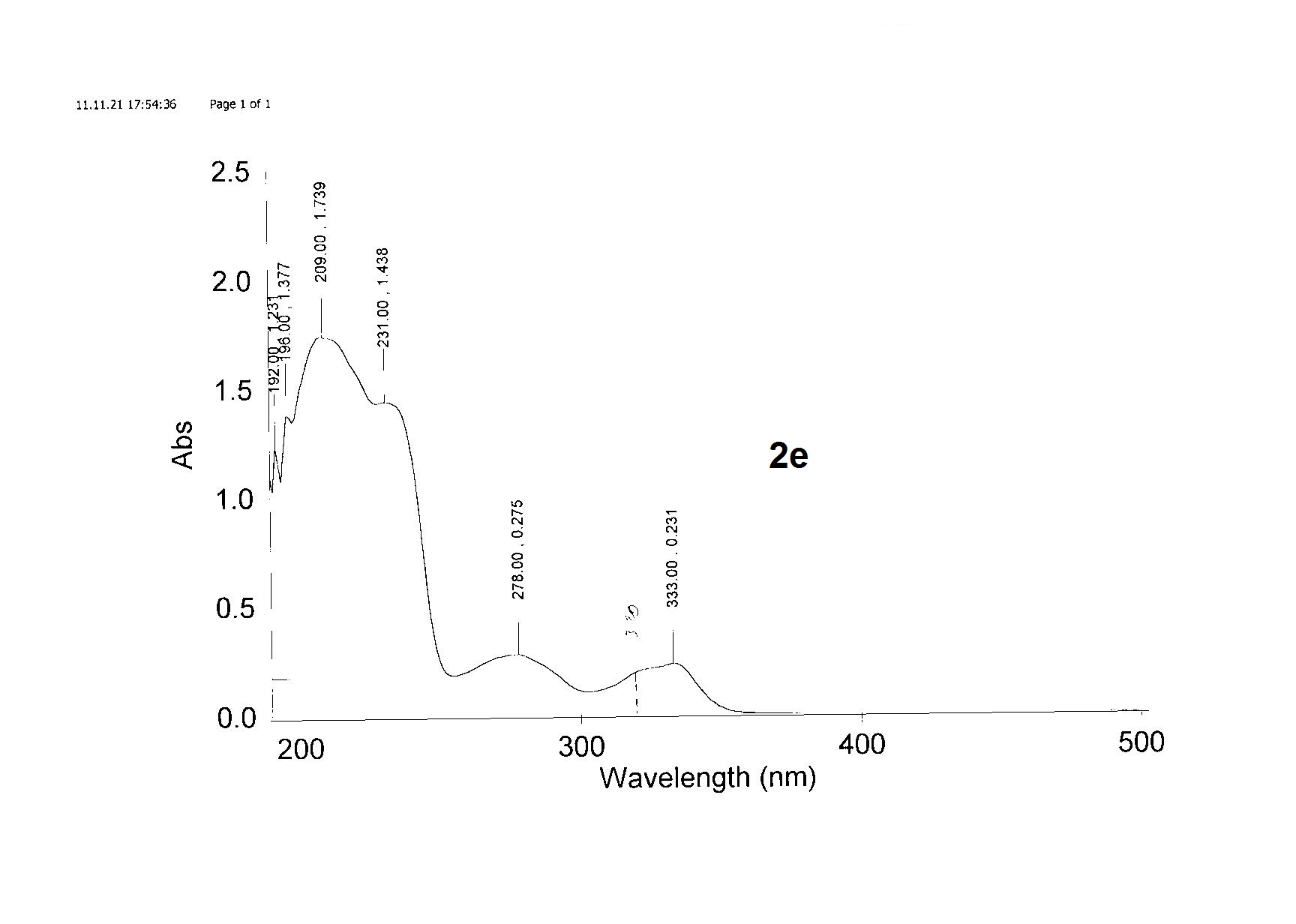

Supplement: Supplementary file 1 [file molecules-27-03476-s001.zip › UV/2e uv.jpg]

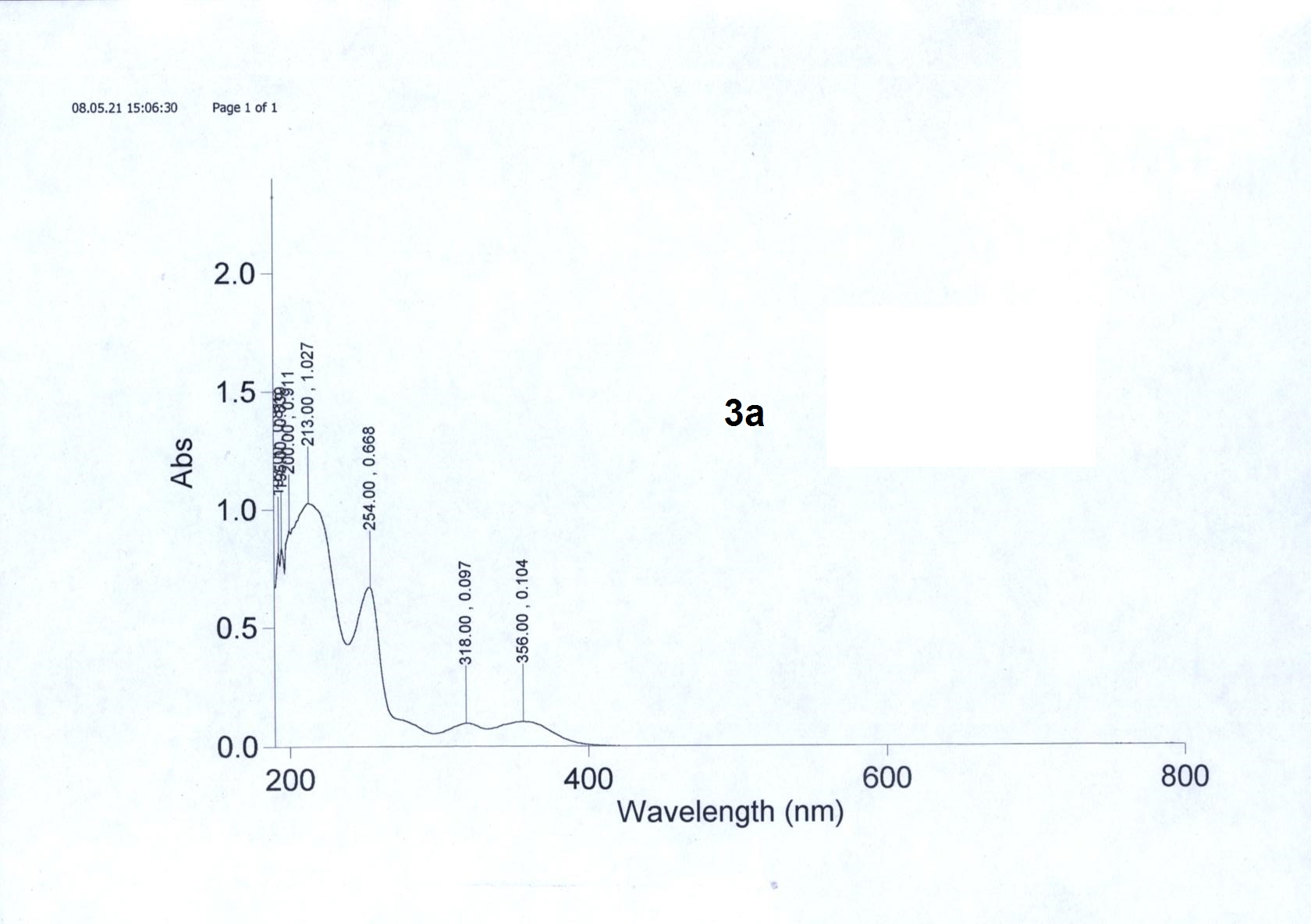

Supplement: Supplementary file 1 [file molecules-27-03476-s001.zip › UV/3a UV.jpg]

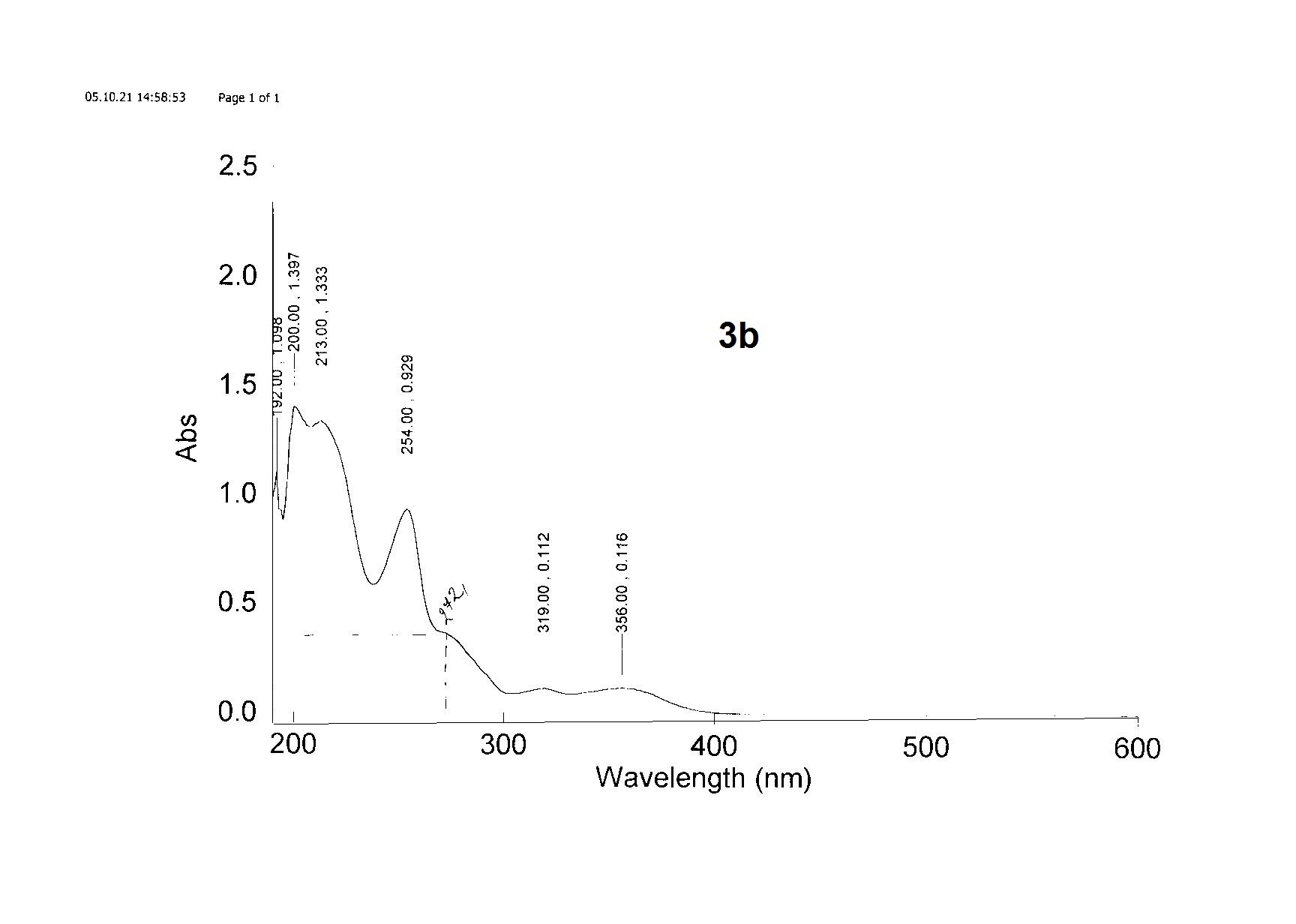

Supplement: Supplementary file 1 [file molecules-27-03476-s001.zip › UV/3b UV.jpg]

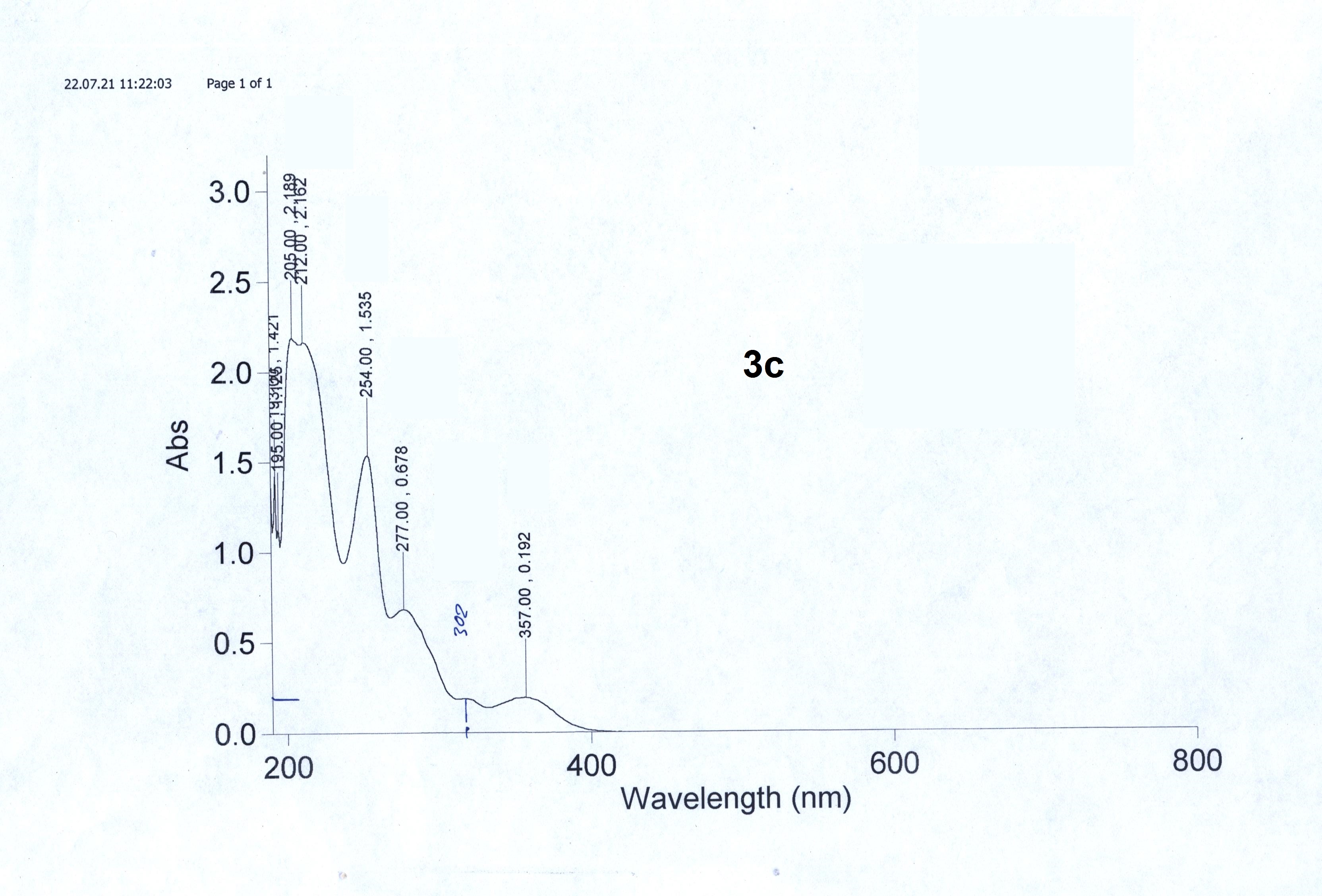

Supplement: Supplementary file 1 [file molecules-27-03476-s001.zip › UV/3c UV.jpg]

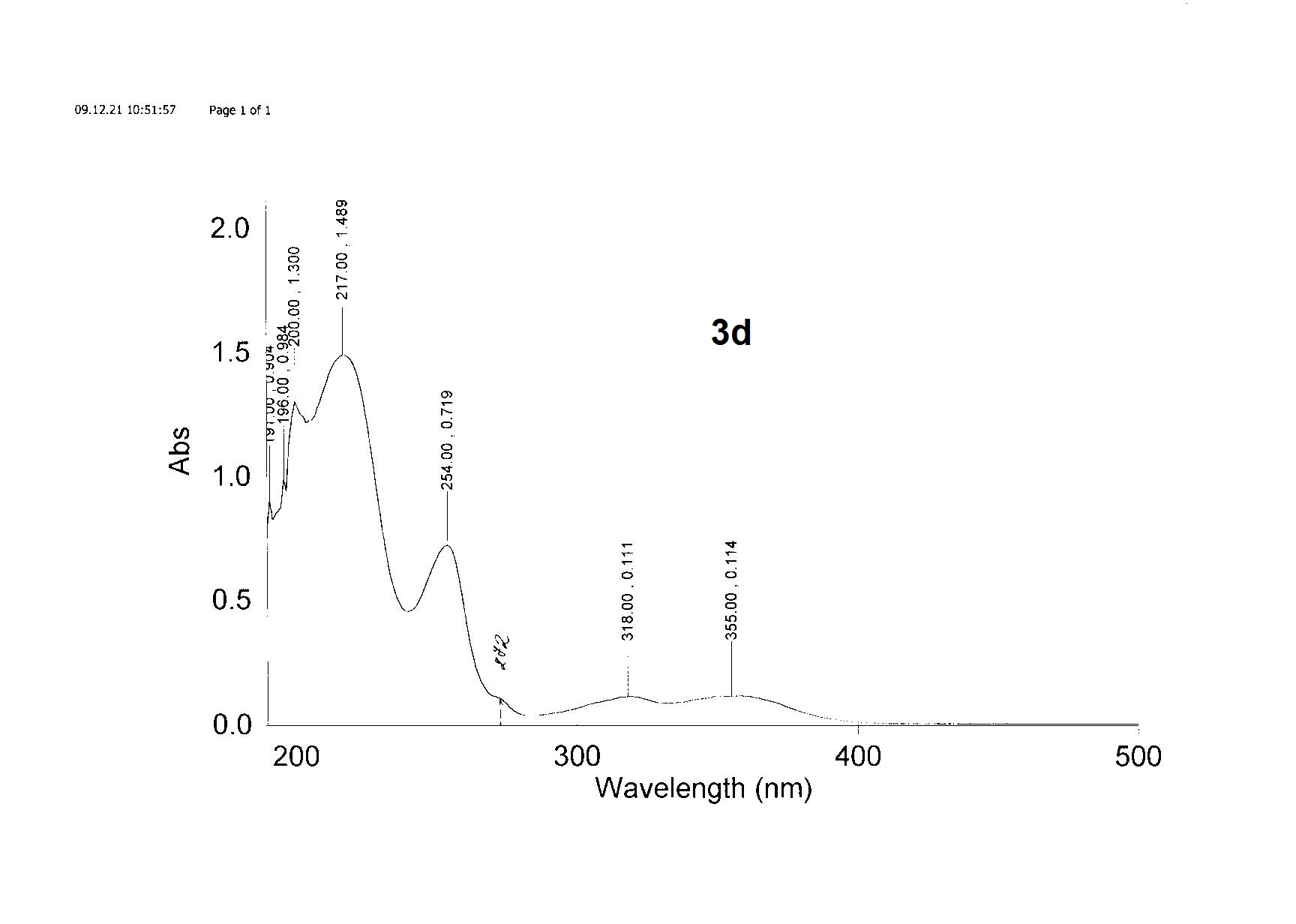

Supplement: Supplementary file 1 [file molecules-27-03476-s001.zip › UV/3d uv.jpg]

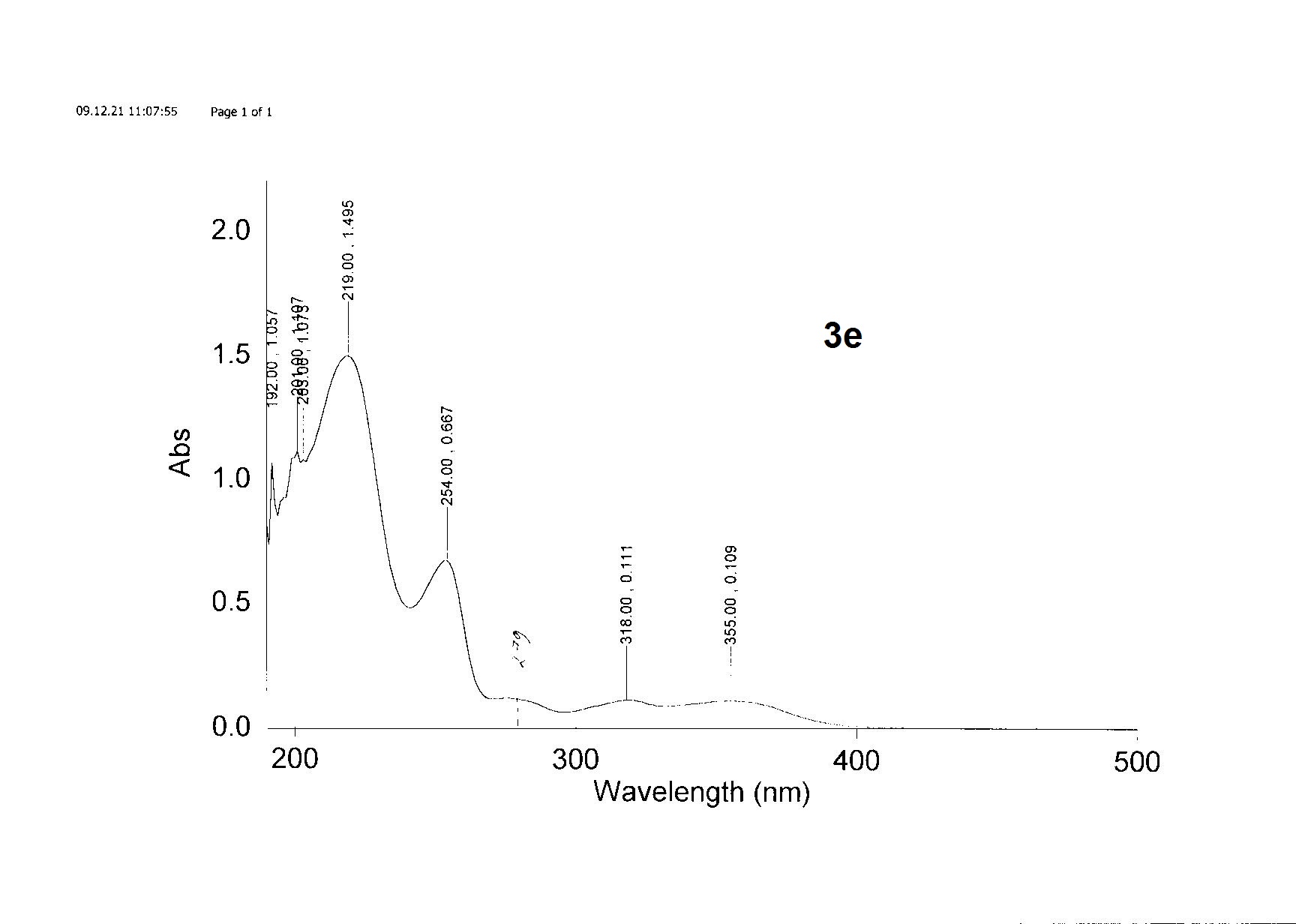

Supplement: Supplementary file 1 [file molecules-27-03476-s001.zip › UV/3e uv.jpg]
